# Supplementary material for: Convenient Synthesis of Pyrazolo[4′,3′:5,6]pyrano[4,3-c][1,2]oxazoles via Intramolecular Nitrile Oxide Cycloaddition
Source: Molecules. 2021 Sep 15;26(18):5604. doi: 10.3390/molecules26185604 (PMC8469150; doi:10.3390/molecules26185604)
Supplement: Supplementary file 1 [file molecules-26-05604-s001.zip › molecules-1377369-supplementary.pdf]

Supplementary Materials

Convenient Synthesis of Pyrazolo[4',3':5,6]pyrano[4,3-*c*][1,2]oxazoles via Intramolecular Nitrile Oxide Cycloaddition

Vaida Milišiūnaitė[1], Elena Plytninkienė[1,2], Roberta Bakšienė[2], Aurimas Bieliauskas[1], Sonata Krikštolaitytė[2], Greta Račkauskienė[1], Eglė Arbačiauskienė[2,*], Algirdas Šačkus[1,*]

[1] Institute of Synthetic Chemistry, Kaunas University of Technology,
K. Baršausko g. 59, Kaunas LT-51423, Lithuania;
[2] Department of Organic Chemistry, Kaunas University of Technology,
Radvilėnų pl. 19, Kaunas LT-50254, Lithuania;

* Corresponding authors
E-mail address: algirdas.sackus@ktu.lt (A. Šačkus), egle.arbaciauskiene@ktu.lt (E. Arbačiauskienė)

Comp. No.	Ratio*	OH, δ_H ppm	Pyrazole H-5, δ_H ppm	Iminyl-H, δ_H ppm	Iminyl-C, δ_C ppm	Iminyl-C $^1J_{CH}$, Hz	Iminyl-N, δ_N ppm**
syn-**4a** (*Z*)	97	11.63	8.85	7.28	134.90	174.4	-19.2
anti-**4a** (*E*)	3	10.97	8.60	7.93	138.93	160.0	-16.5
syn-**4b** (*Z*)	91	11.64	8.84	7.28	134.88	176.5	-19.2
anti-**4b** (*E*)	9	10.99	8.58	7.93	138.87	161.8	-16.5
syn-**4c** (*Z*)	99	11.67	8.88	7.27	134.74	175.8	-18.2
anti-**4c** (*E*)	1	11.01	8.63	7.91	138.77	165.7	-15.6
syn-**4d** (*Z*)	100	11.21	8.20	7.15	135.56	174.5	-25.7
anti-**4d** (*E*)	–	–	–	–	–	–	–
syn-**9** (*Z*)	97	11.63	8.86	7.32	134.97	177.9	-19.2
anti-**9** (*E*)	3	10.97	8.62	7.95	138.97	159.3	-16.5
syn-**14a** (*Z*)	100	11.65	8.87	7.27	134.70	179.2	-18.4
anti-**14a**(*E*)	–	–	–	–	–	–	–

* Ratio was determined by ^{1}H NMR spectral data after purification by column chromatography. ** External nitromethane (0.0 ppm) was used as a ^{15}N reference compound.

Table S1. Relevant NMR data of aldoximes 4a-d, 9 and 14 (DMSO-*d*$_6$).

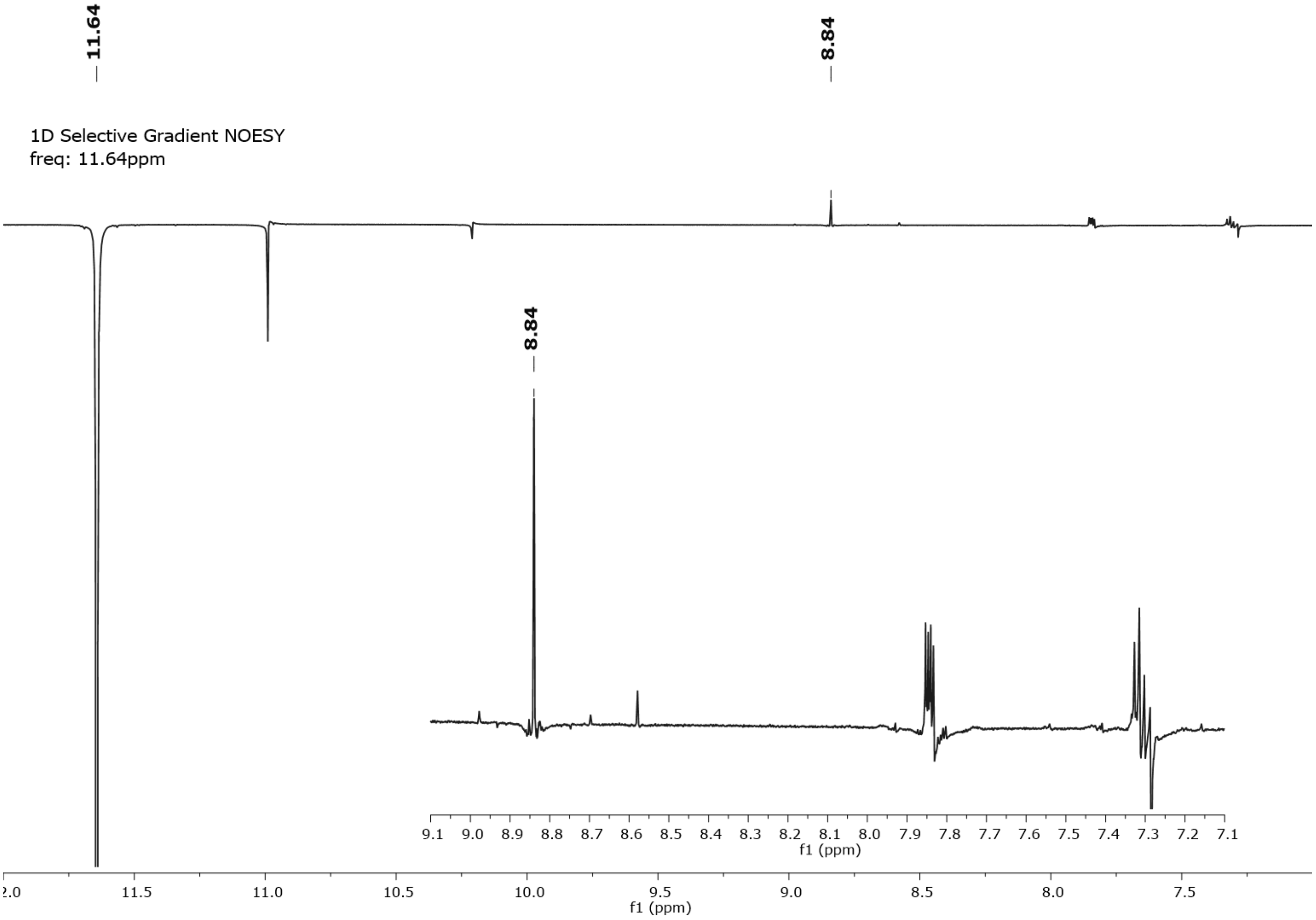

Figure S2. 1D Selective Gradient NOESY spectrum (4b) (700 MHz, DMSO-*d*$_6$).

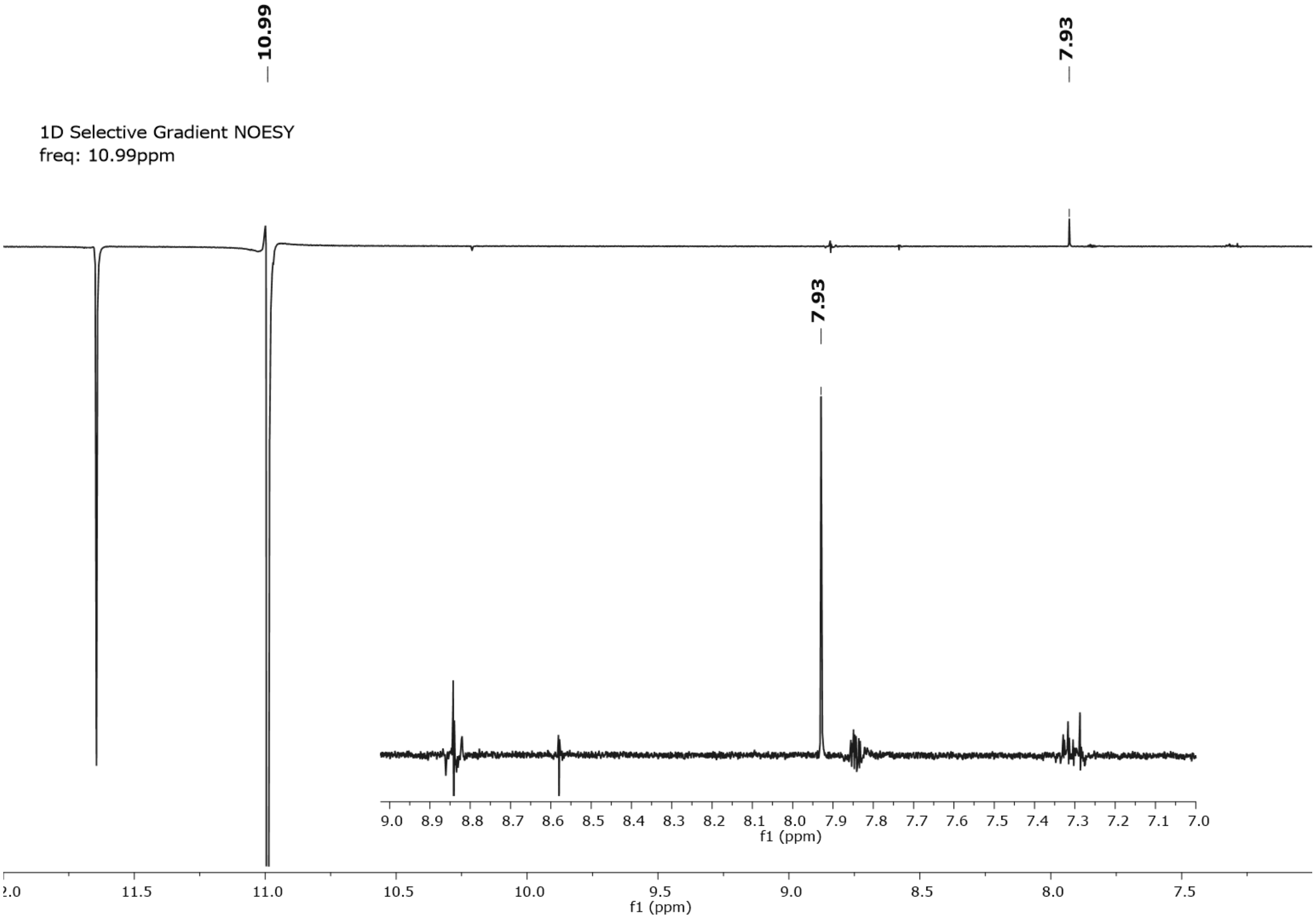

Figure S3. 1D Selective Gradient NOESY spectrum (4b) (700 MHz, DMSO-d_6).

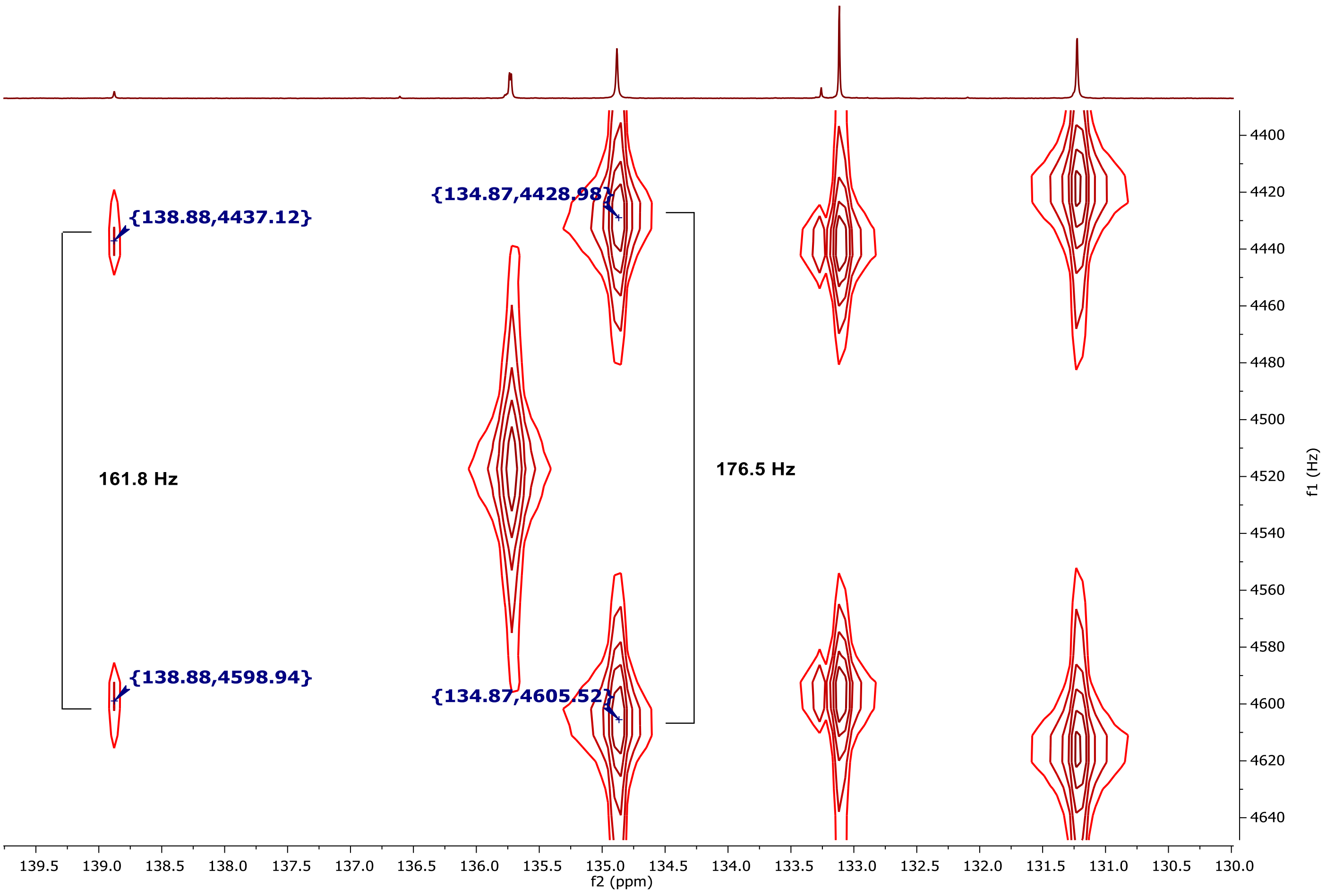

Figure S4. Heteronuclear 2D *J*-resolved NMR spectrum (4b) (700 MHz, DMSO-d_6).

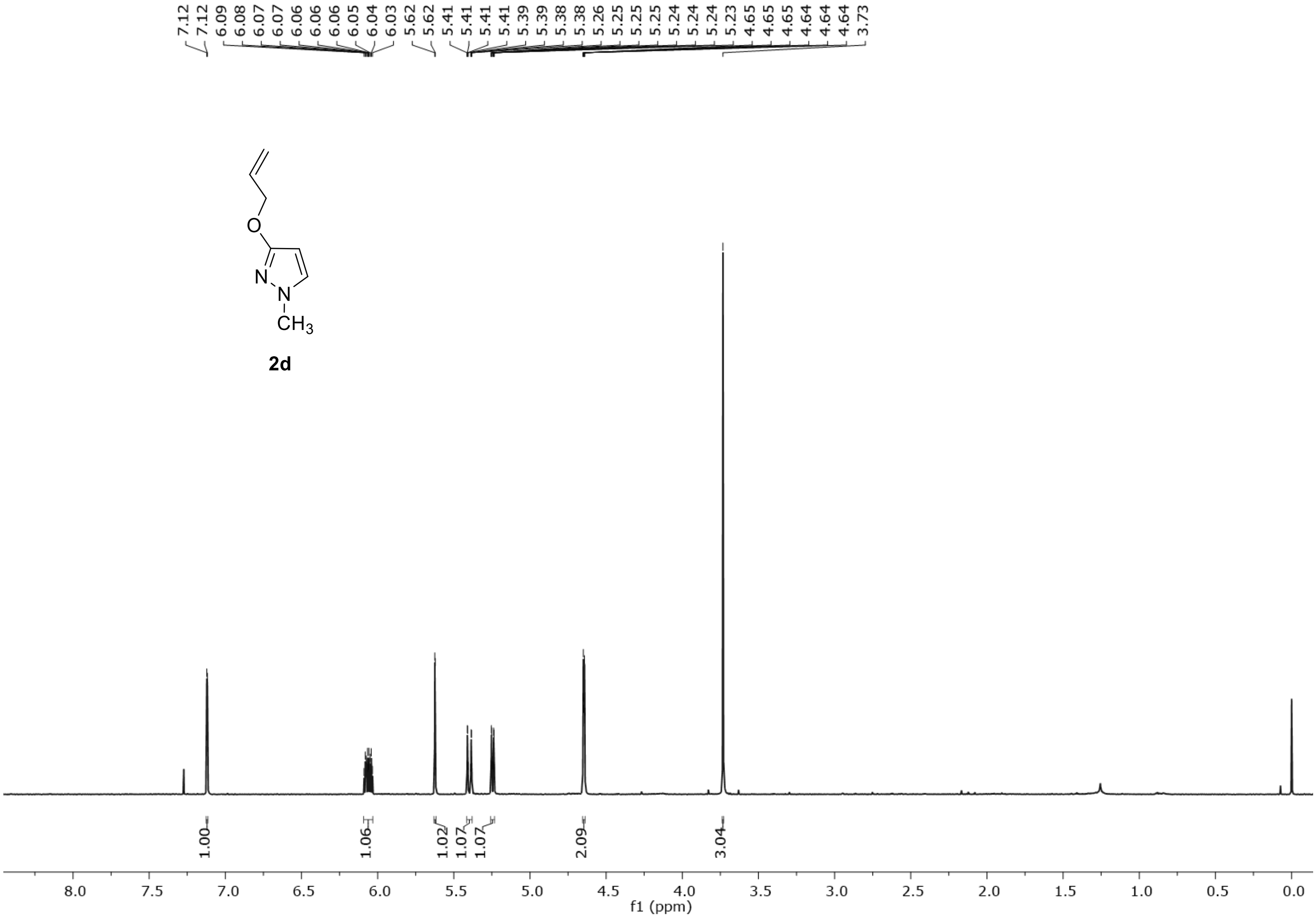

Figure S5. 1-Methyl-3-[(prop-2-en-1-yl)oxy]-1*H*-pyrazole (2d). **^{1}H NMR spectrum (700 MHz, $CDCl_3$).**

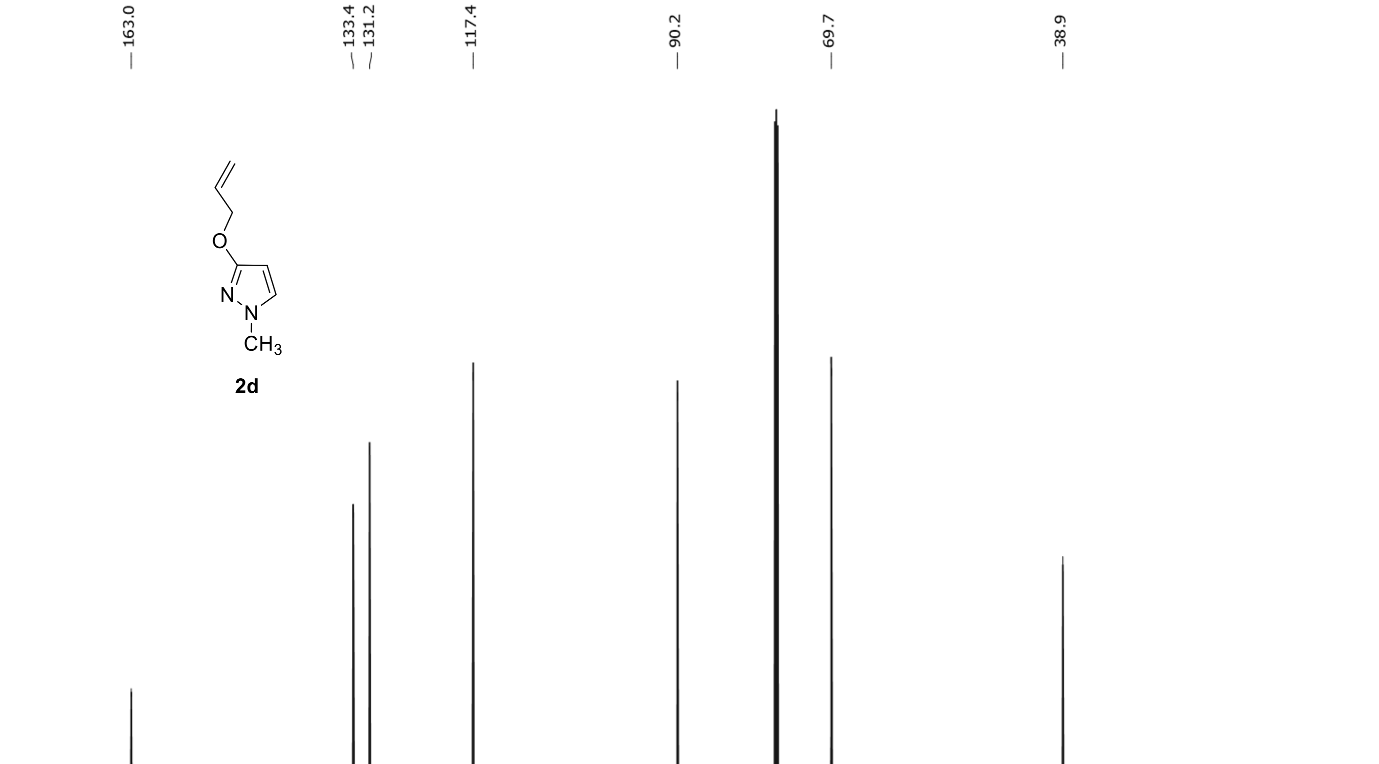

Figure S6. 1-Methyl-3-[(prop-2-en-1-yl)oxy]-1*H*-pyrazole (2d). **^{13}C NMR spectrum (176 MHz, $CDCl_3$).**

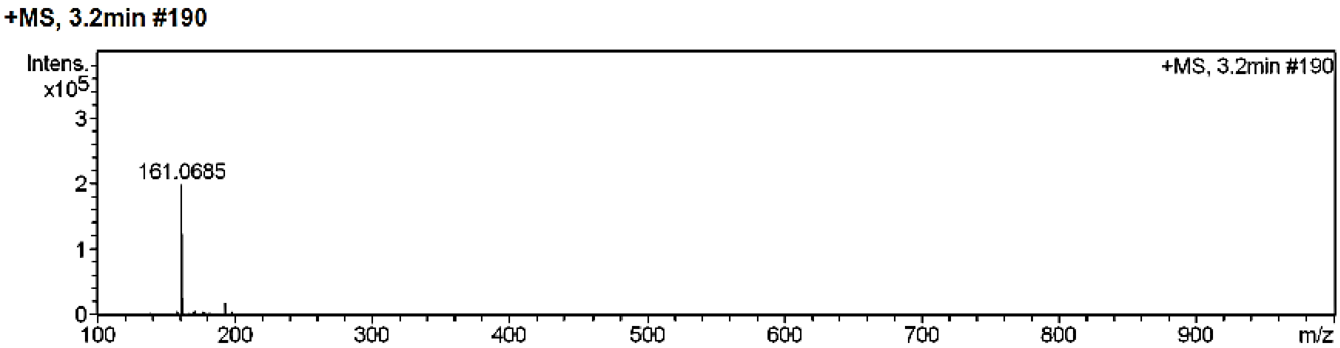

Meas. m/z	#	Ion Formula	m/z	err [ppm]	mSigma	# Sigma	Score	rdb	e¯ Conf	N-Rule
139.0887	1	C7H11N2O	139.0866	-15.1	528.5	1	-1.#J	3.5	even	ok
161.0685	1	C7H10N2NaO	161.0685	-0.1	2.4	1	100.00	3.5	even	ok

Figure S7. 1-Methyl-3-[(prop-2-en-1-yl)oxy]-1*H*-pyrazole (2d). HRMS (ESI-TOF).

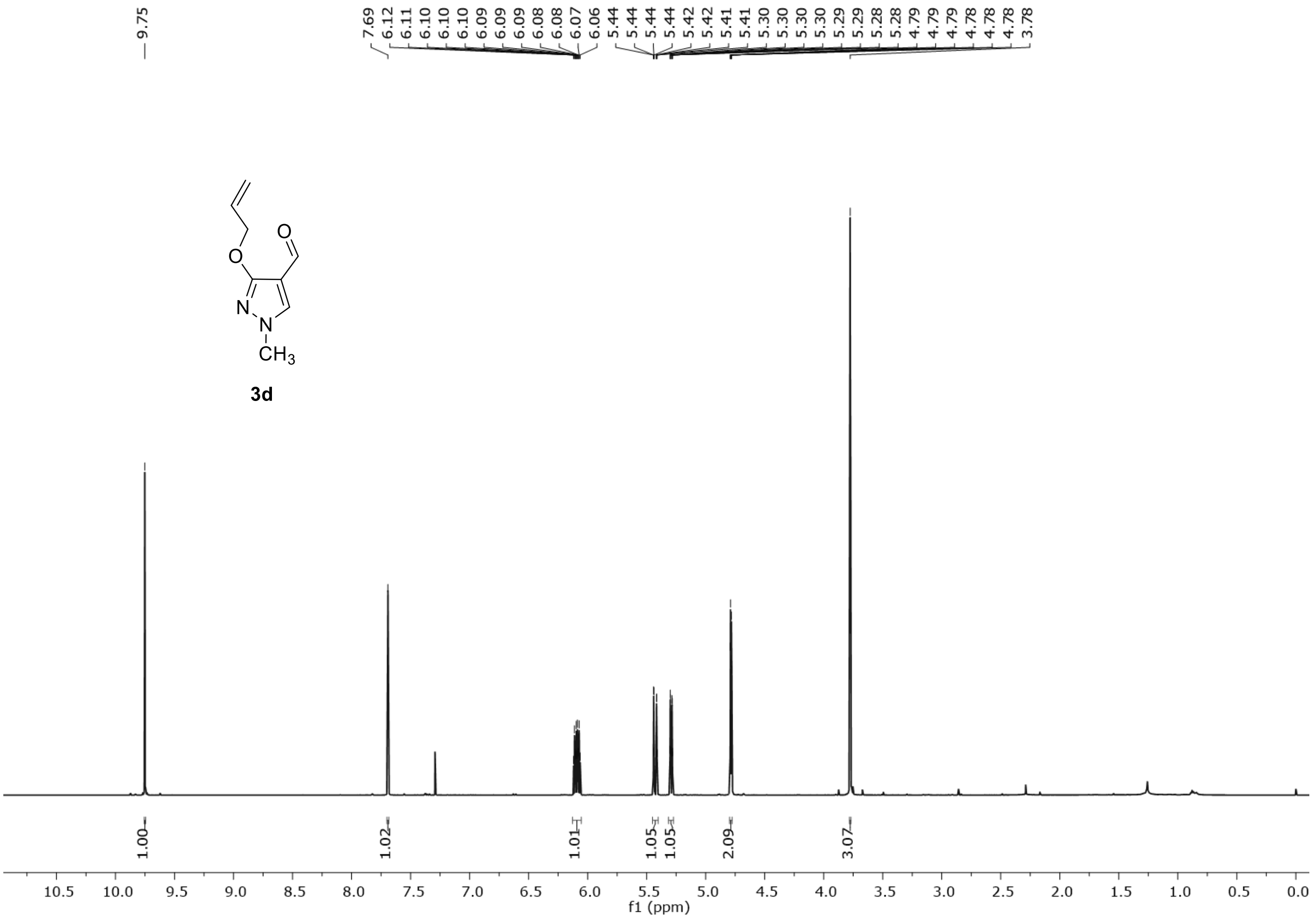

Figure S8. 1-Methyl-3-[(prop-2-en-1-yl)oxy]-1*H*-pyrazole-4-carbaldehyde (3d). ^{1}H NMR spectrum (700 MHz, $CDCl_3$).

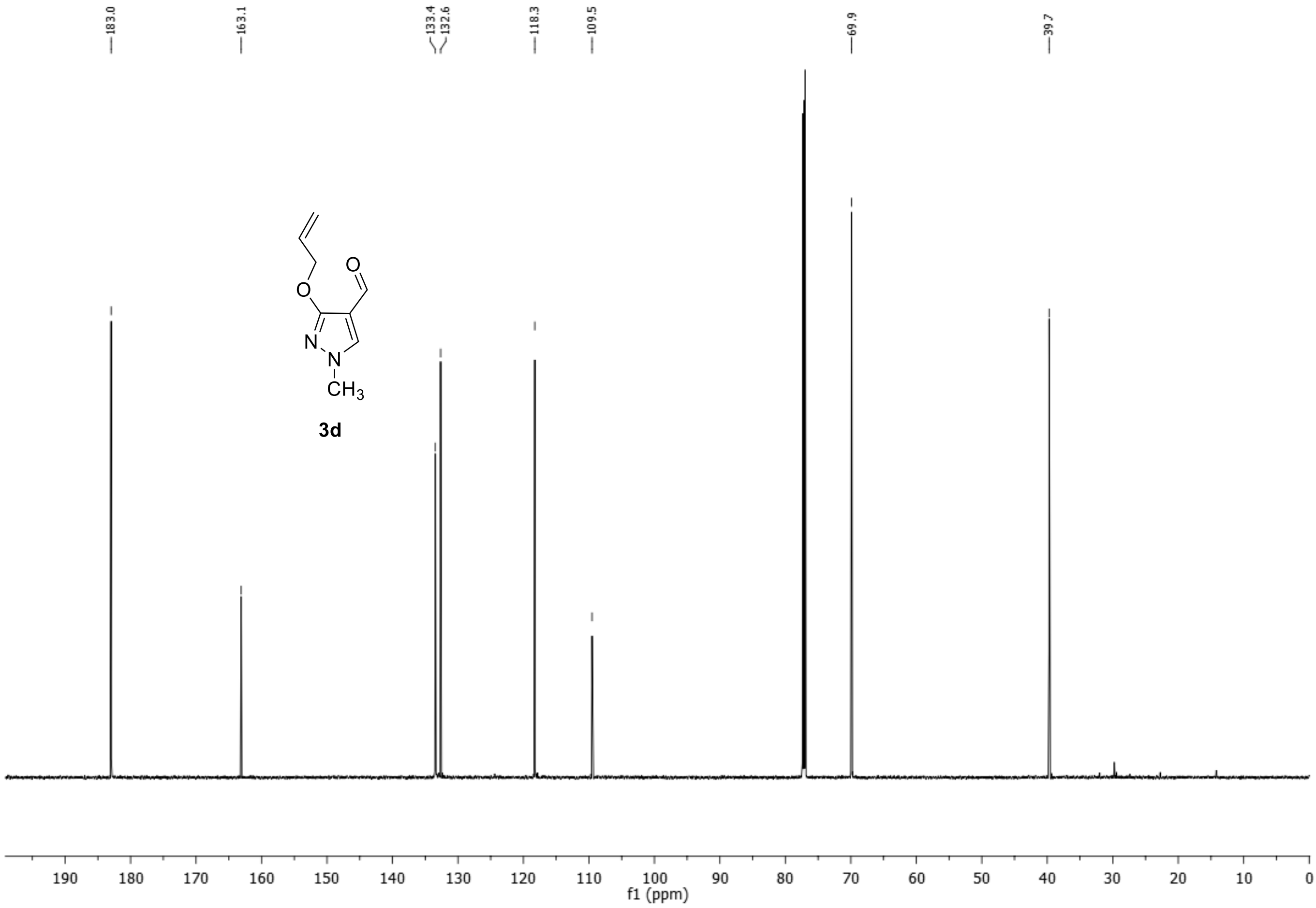

Figure S9. 1-Methyl-3-[(prop-2-en-1-yl)oxy]-1*H*-pyrazole-4-carbaldehyde (3d). ^{13}C NMR spectrum (176 MHz, $CDCl_3$).

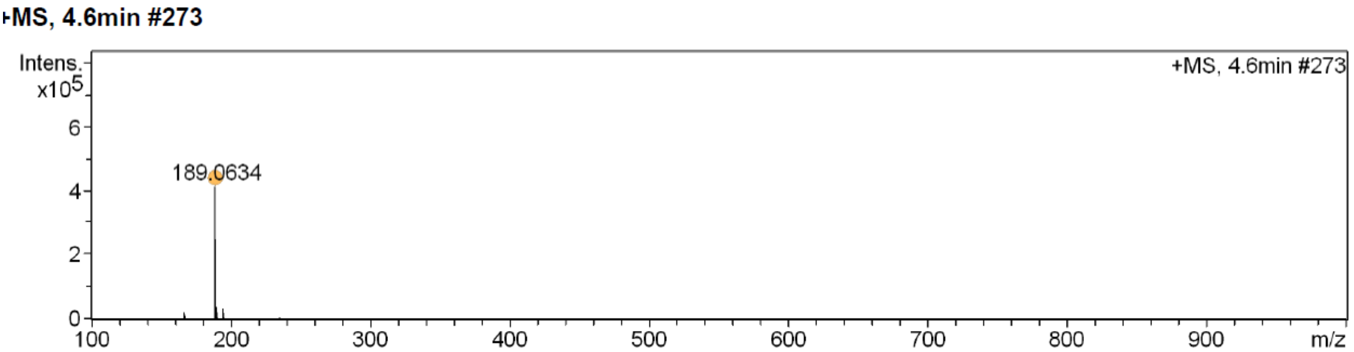

Meas. m/z	#	Ion Formula	m/z	err [ppm]	mSigma	# Sigma	Score	rdb	e⁻ Conf	N-Rule
189.0634	1	C8H10N2NaO2	189.0634	-0.2	1.5	1	100.00	4.5	even	ok

Figure S10. 1-Methyl-3-[(prop-2-en-1-yl)oxy]-1*H*-pyrazole-4-carbaldehyde (3d). HRMS (ESI-TOF).

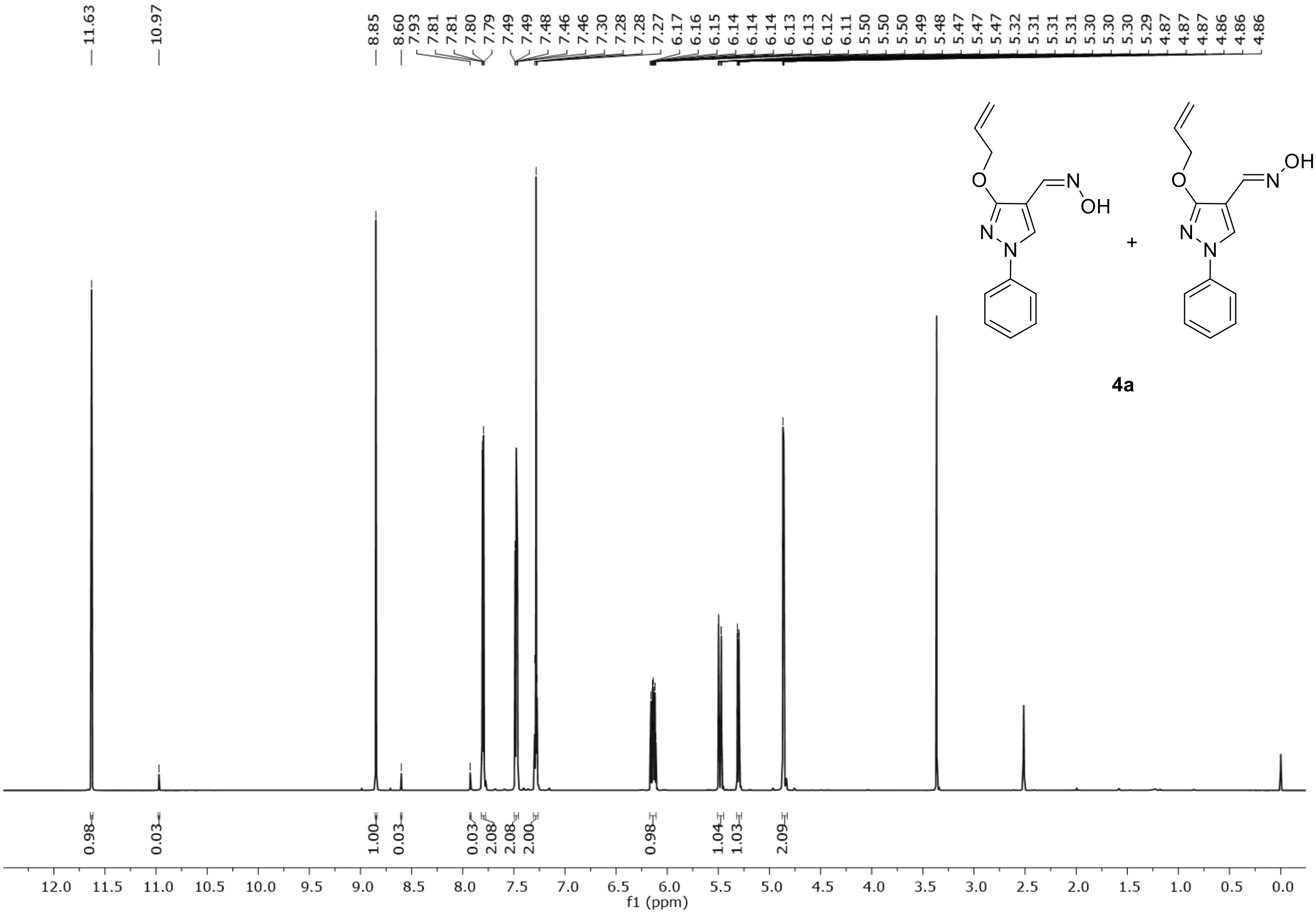

Figure S11. ***N*-[(*Z/E*)-{1-Phenyl-3-[(prop-2-en-1-yl)oxy]-1*H*-pyrazol-4-yl}methylidene]hydroxylamine (4a). ^{1}H NMR spectrum (700 MHz, DMSO-d_6).**

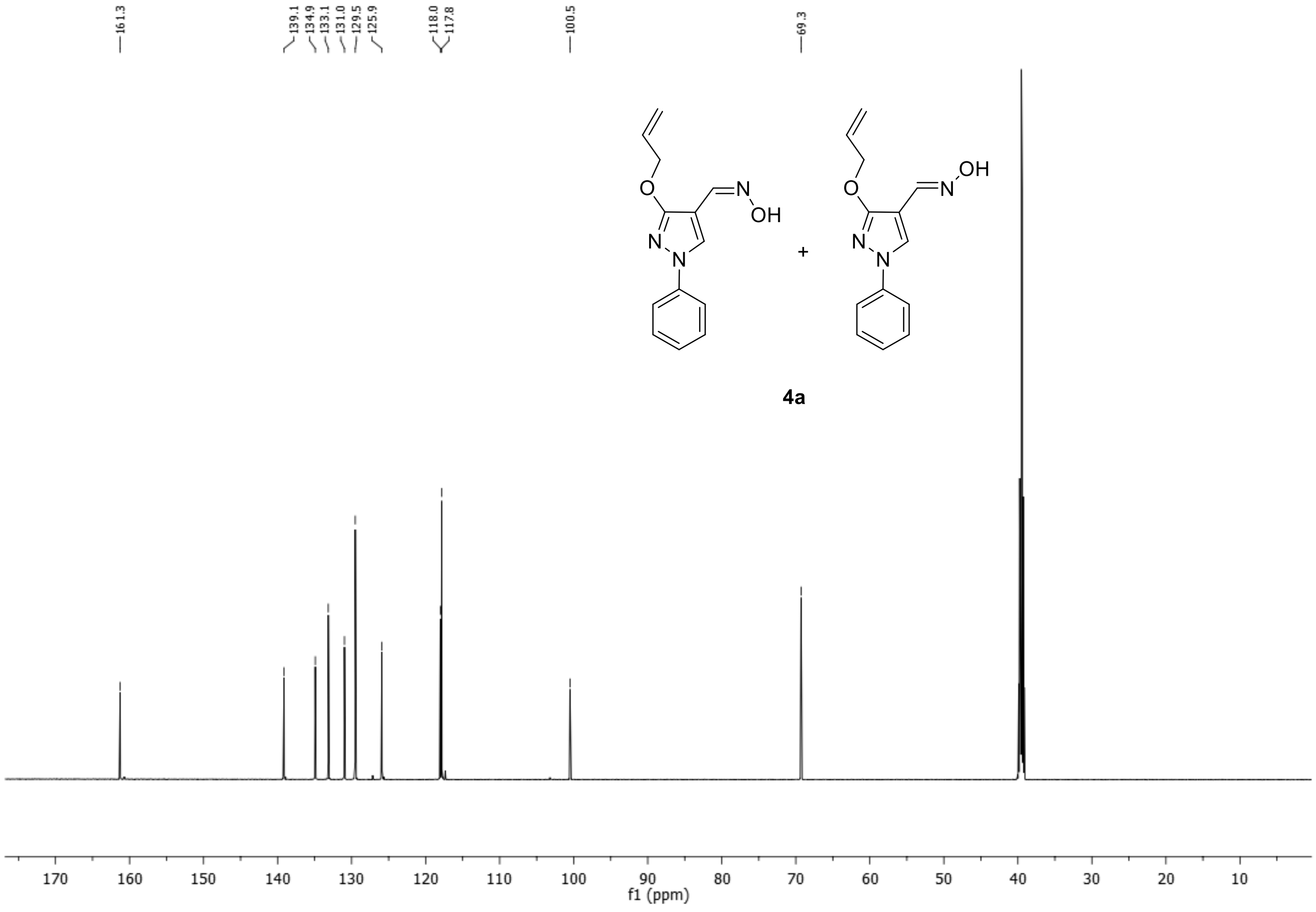

Figure S12. ***N*-[(*Z/E*)-{1-Phenyl-3-[(prop-2-en-1-yl)oxy]-1*H*-pyrazol-4-yl}methylidene]hydroxylamine (4a). ^{13}C NMR spectrum (176 MHz, DMSO-d_6).**

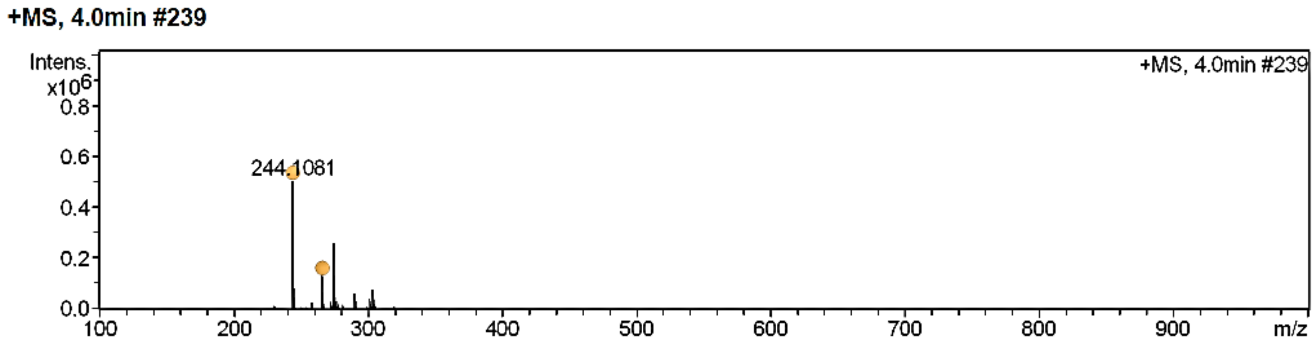

Meas. m/z	#	Ion Formula	m/z	err [ppm]	mSigma	# Sigma	Score	rdb	e¯ Conf	N-Rule
244.1081	1	C13H14N3O2	244.1081	-0.0	4.3	1	100.00	8.5	even	ok
266.0904	1	C13H13N3NaO2	266.0900	-1.6	1.9	1	100.00	8.5	even	ok

Figure S13. ***N*-[(*Z*/*E*)-{1-Phenyl-3-[(prop-2-en-1-yl)oxy]-1*H*-pyrazol-4-yl}methylidene]hydroxylamine (4a). HRMS (ESI-TOF).**

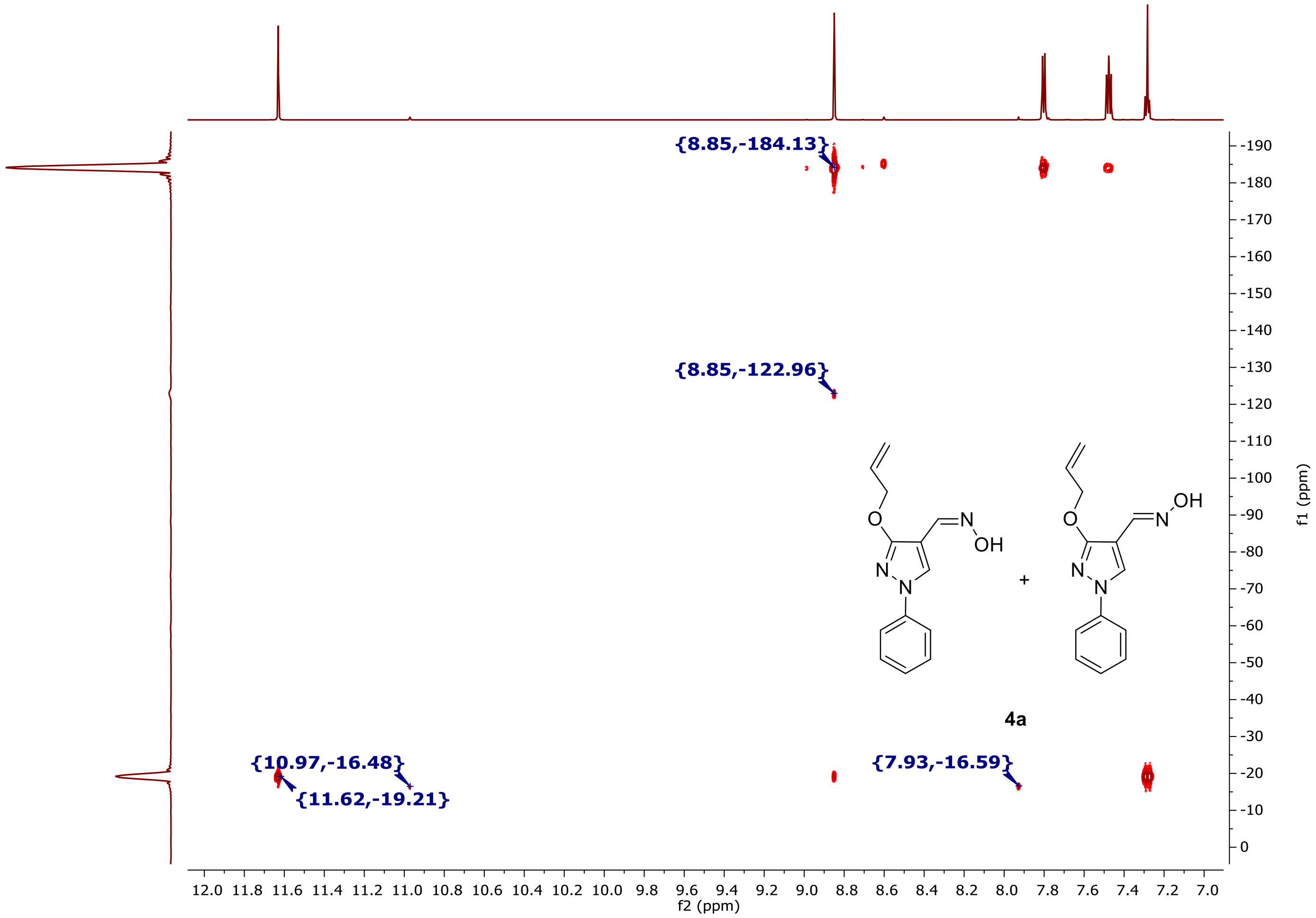

Figure S14. ***N*-[(*Z*/*E*)-{1-Phenyl-3-[(prop-2-en-1-yl)oxy]-1*H*-pyrazol-4-yl}methylidene]hydroxylamine (4a). ^{1}H-^{15}N HMBC spectrum (71 MHz, DMSO-*d*$_6$).**

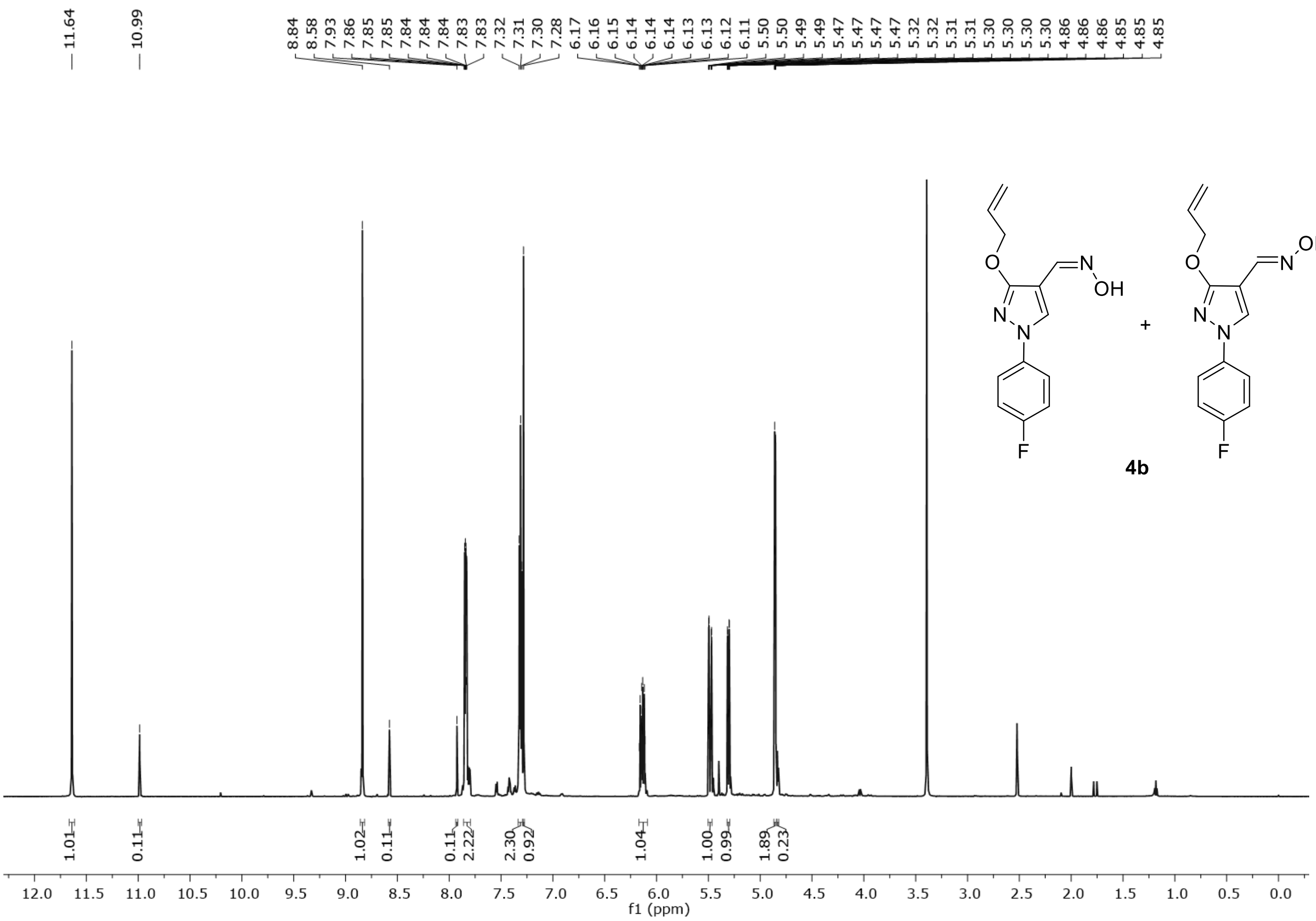

Figure S15. ***N*-[(*Z/E*)-[4-Fluorophenyl)-3-[(prop-2-en-1-yl)oxy}-1*H*-pyrazol-4-yl}methylidene] hydroxylamine (4b). ^{1}H NMR spectrum (700 MHz, DMSO-*d*$_6$).**

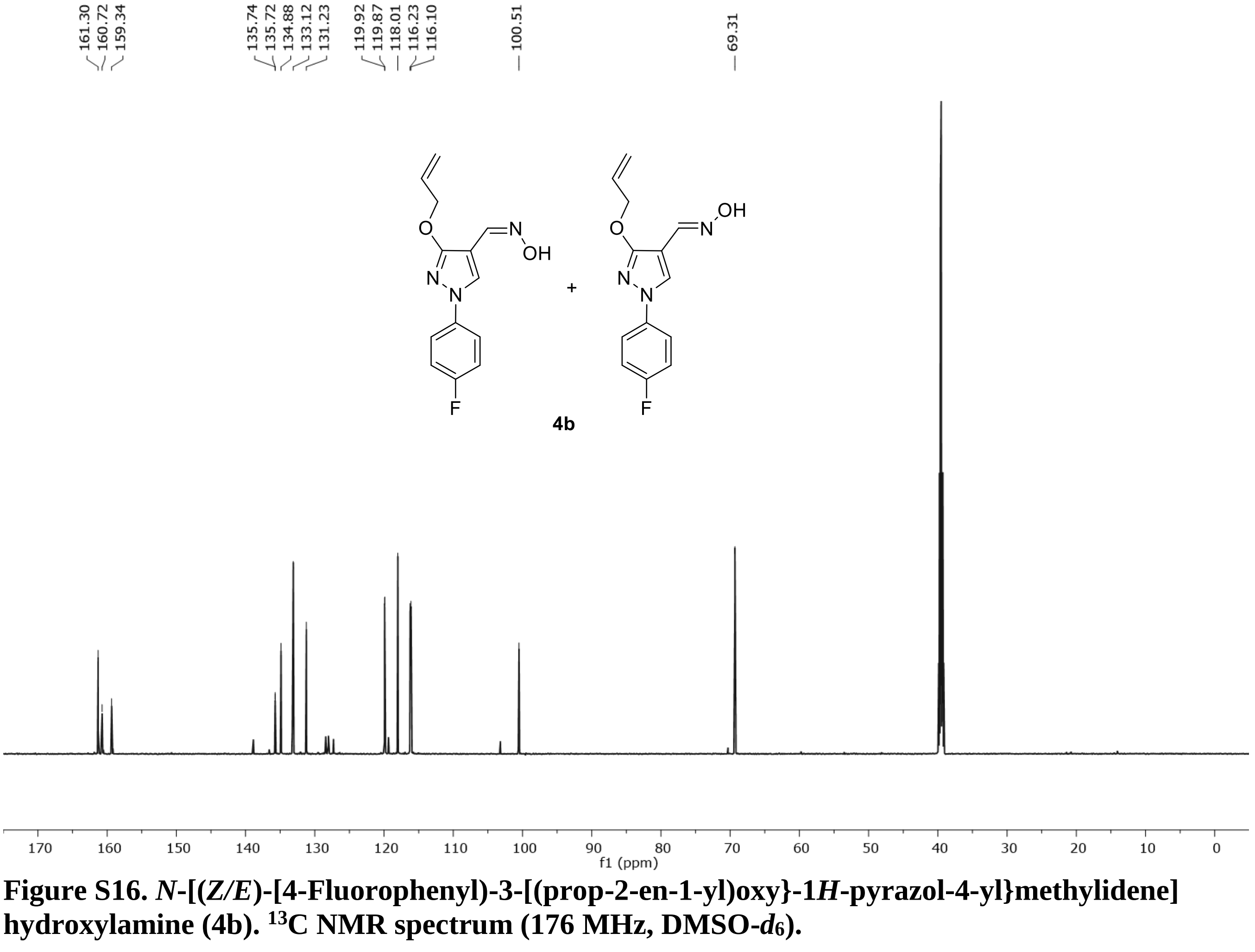

Figure S16. ***N*-[(*Z/E*)-[4-Fluorophenyl)-3-[(prop-2-en-1-yl)oxy}-1*H*-pyrazol-4-yl}methylidene] hydroxylamine (4b). ^{13}C NMR spectrum (176 MHz, DMSO-*d*$_6$).**

+MS, 5.8min #348

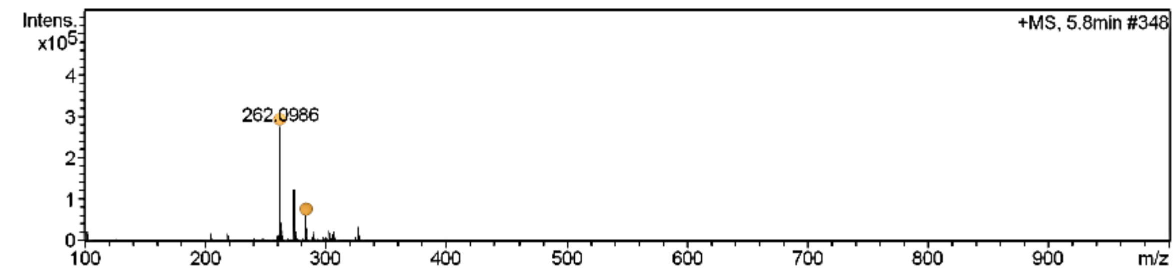

Meas. m/z	#	Ion Formula	m/z	err [ppm]	mSigma	# Sigma	Score	rdb	e⁻ Conf	N-Rule
262.0986	1	C13H13FN3O2	262.0986	0.2	11.1	1	100.00	8.5	even	ok
284.0811	1	C13H12FN3NaO2	284.0806	1.9	4.1	1	100.00	8.5	even	ok

Figure S17. ***N*-[(*Z*/*E*)-[4-Fluorophenyl)-3-[(prop-2-en-1-yl)oxy}-1*H*-pyrazol-4-yl}methylidene] hydroxylamine (4b). HRMS (ESI-TOF).**

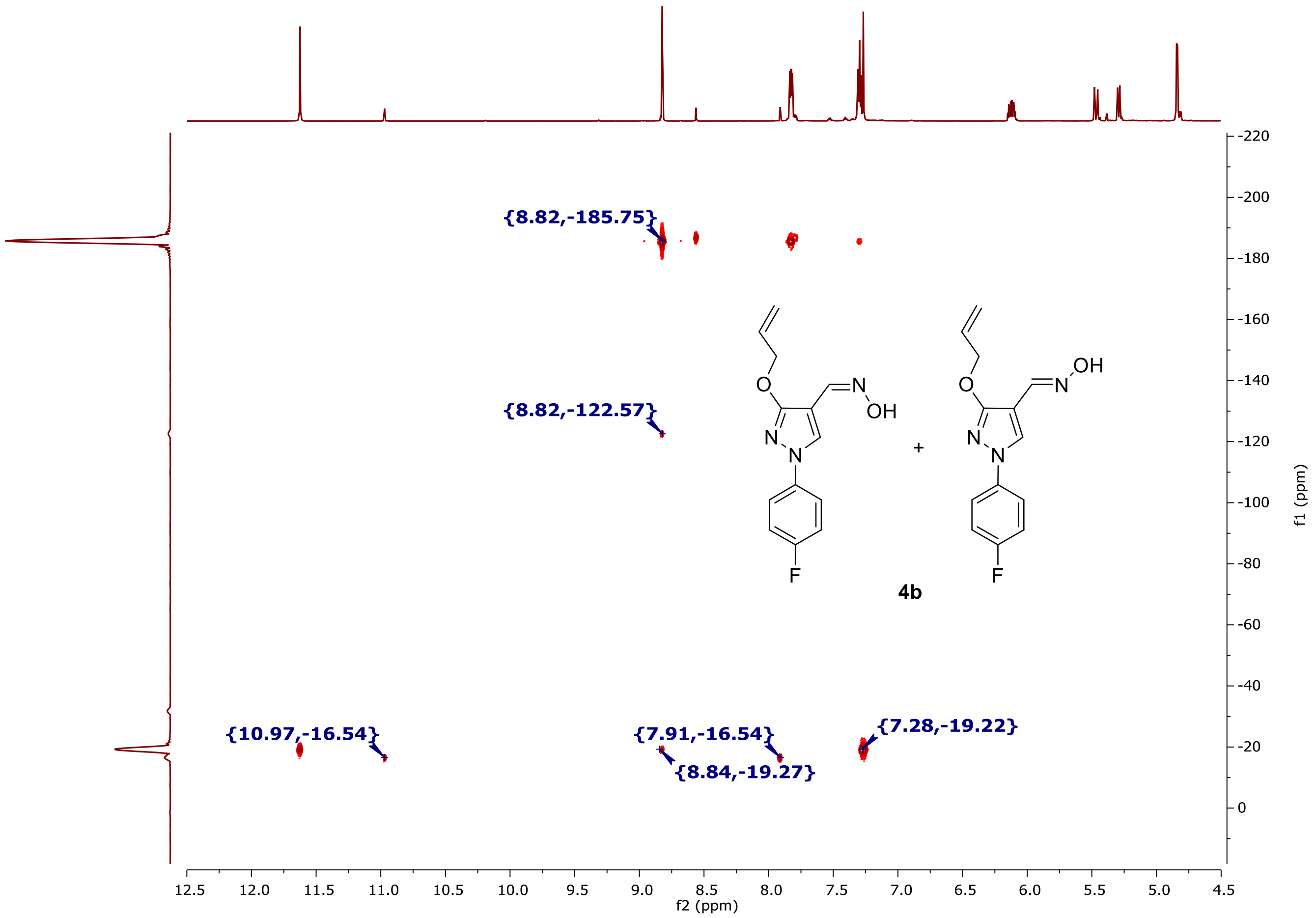

Figure S18. ***N*-[(*Z*/*E*)-[4-Fluorophenyl)-3-[(prop-2-en-1-yl)oxy}-1*H*-pyrazol-4-yl}methylidene] hydroxylamine (4b). ^{1}H-^{15}N HMBC spectrum (71 MHz, DMSO-*d*$_6$).**

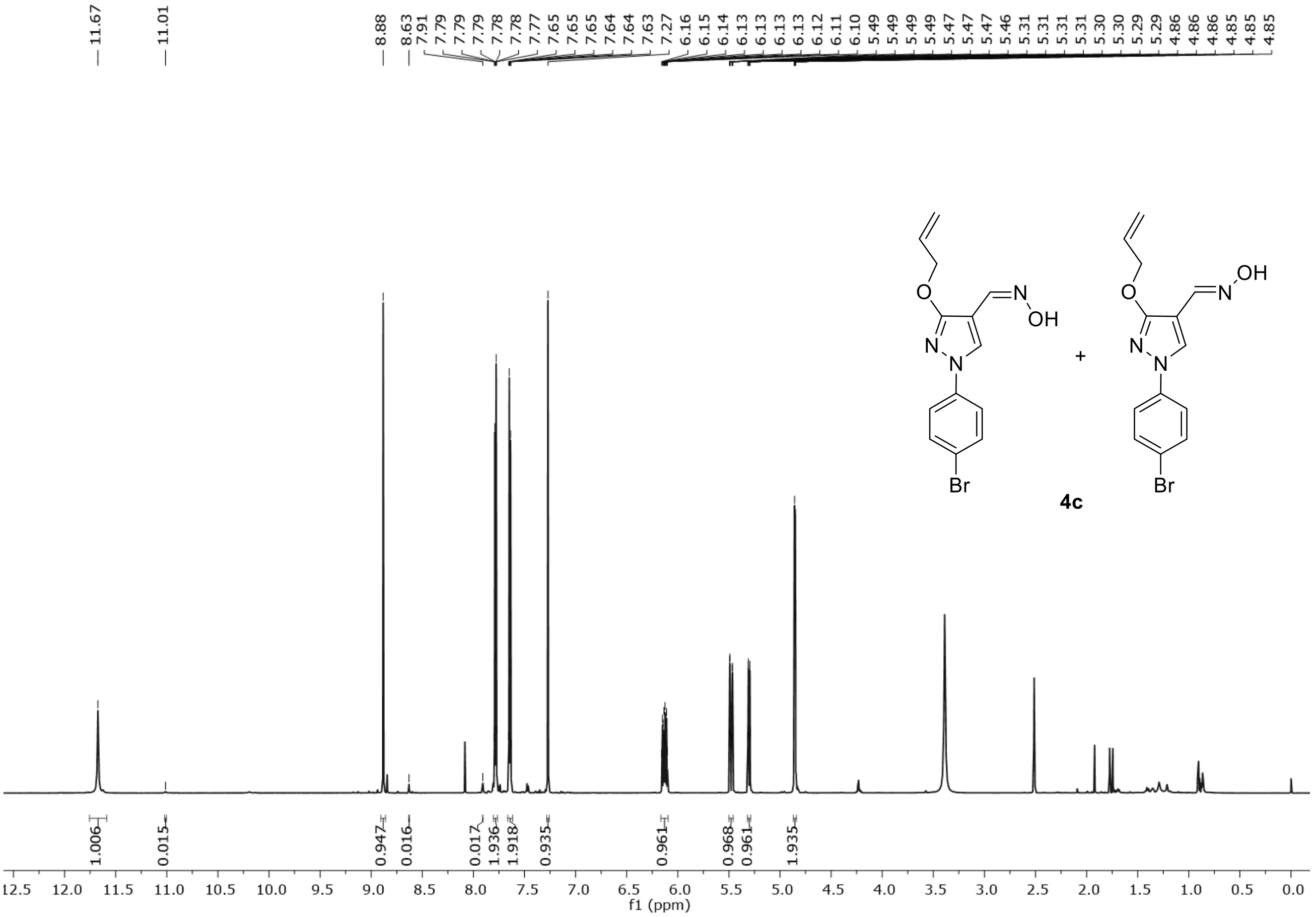

Figure S19. ***N*-[(*Z/E*)-[4-Bromophenyl)-3-[(prop-2-en-1-yl)oxy}-1*H*-pyrazol-4-yl}methylidene] hydroxylamine (4c). ^{1}H NMR spectrum (700 MHz, DMSO-*d*$_6$).**

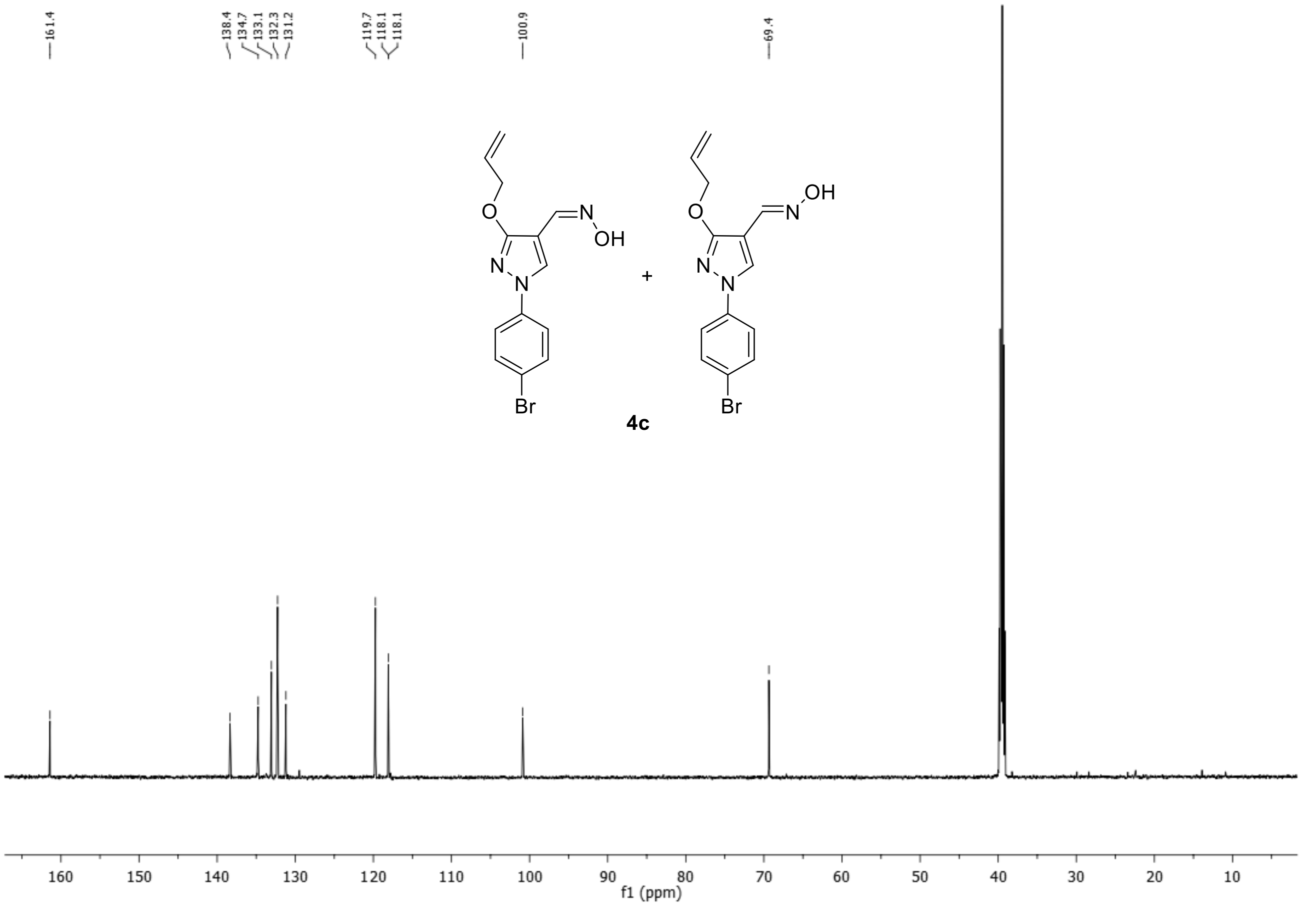

Figure S20. ***N*-[(*Z/E*)-[4-Bromophenyl)-3-[(prop-2-en-1-yl)oxy}-1*H*-pyrazol-4-yl}methylidene] hydroxylamine (4c). ^{13}C NMR spectrum (176 MHz, DMSO-*d*$_6$).**

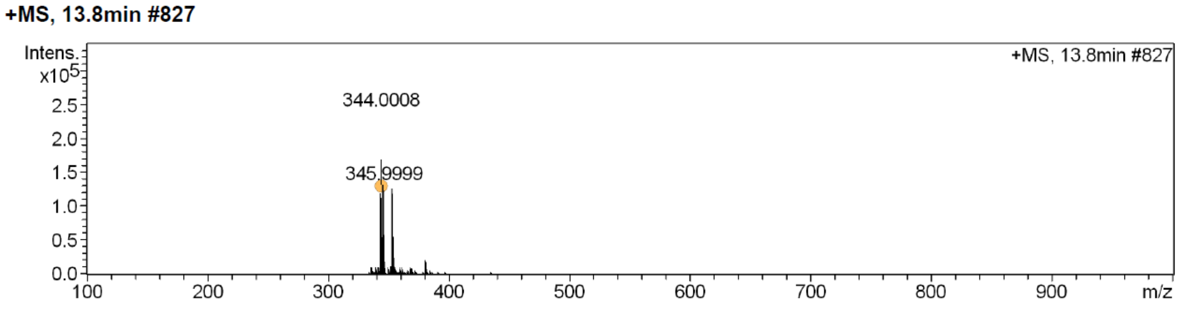

Meas. m/z	#	Ion Formula	m/z	err [ppm]	mSigma	# Sigma	Score	rdb	e⁻ Conf	N-Rule
344.0008	1	C13H12BrN3NaO2	344.0005	0.9	39.6	1	100.00	8.5	even	ok

Figure S21. ***N*-[(*Z*/*E*)-[4-Bromophenyl)-3-[(prop-2-en-1-yl)oxy}-1*H*-pyrazol-4-yl}methylidene] hydroxylamine (4c). HRMS (ESI-TOF).**

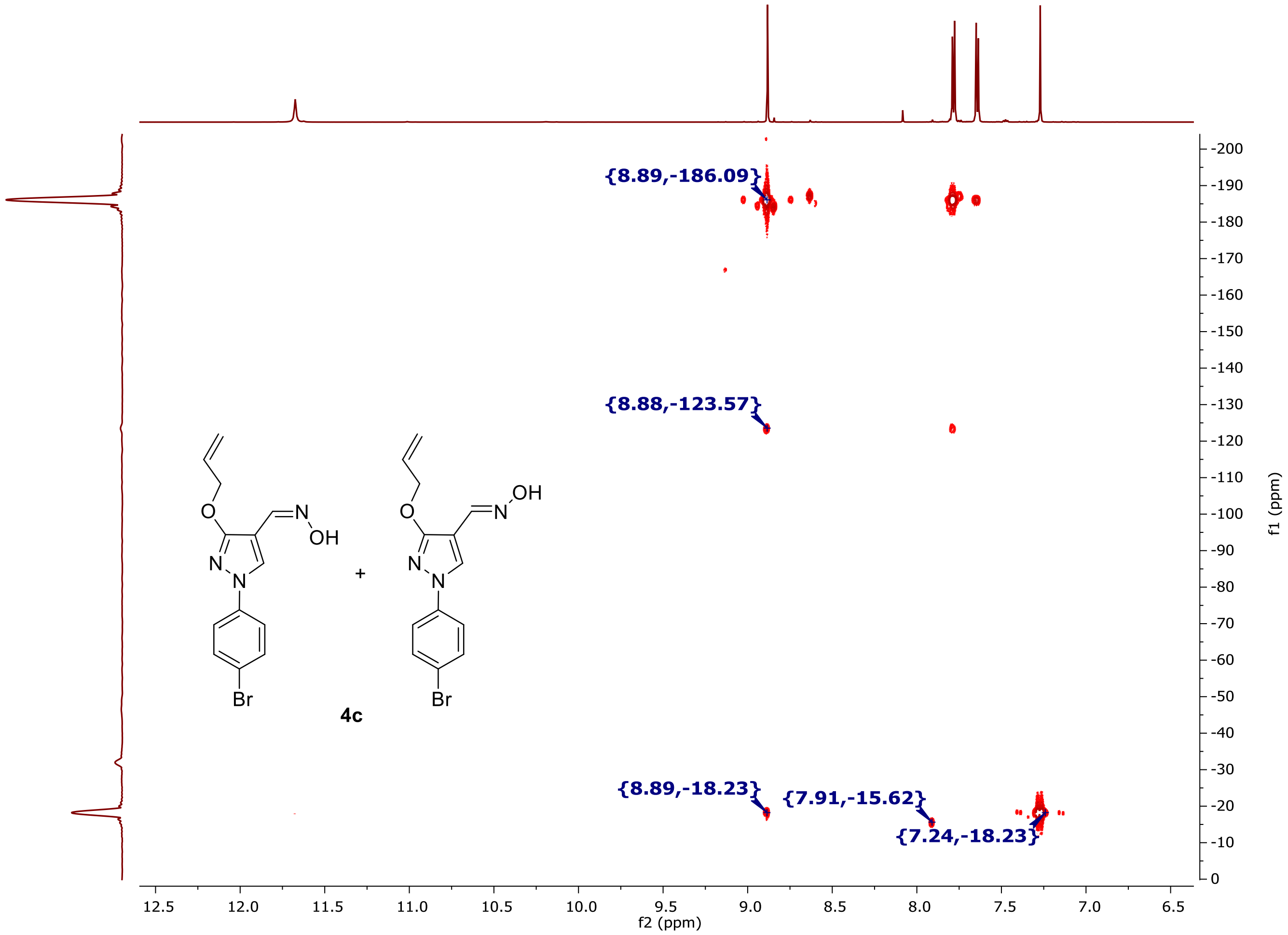

Figure S22. ***N*-[(*Z*/*E*)-[4-Bromophenyl)-3-[(prop-2-en-1-yl)oxy}-1*H*-pyrazol-4-yl}methylidene] hydroxylamine (4c). ^{1}H-^{15}N HMBC spectrum (71 MHz, DMSO-*d*6).**

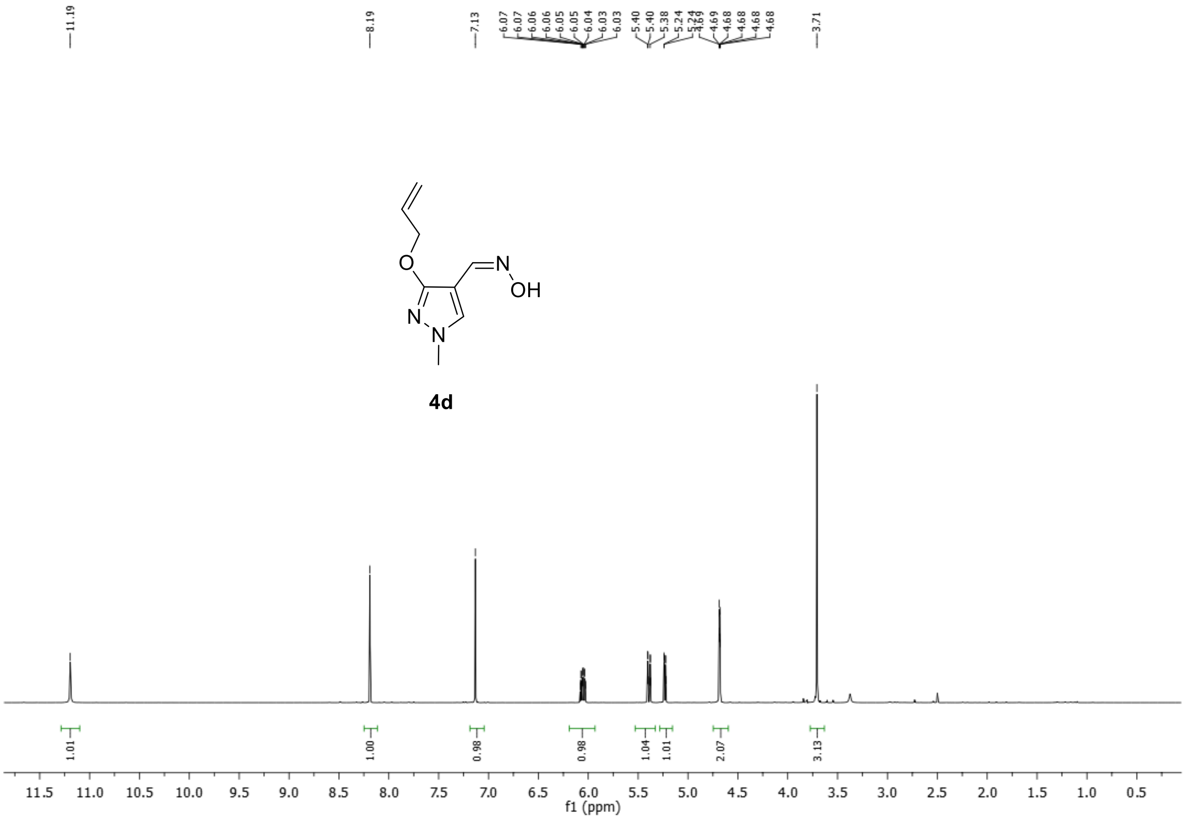

Figure S23. ***N*-[(*Z*)-{1-Methyl-3-[(prop-2-en-1-yl)oxy]-1*H*-pyrazol-4-yl}methylidene]hydroxylamine (4d). ^{1}H NMR spectrum (700 MHz, DMSO-*d*$_6$).**

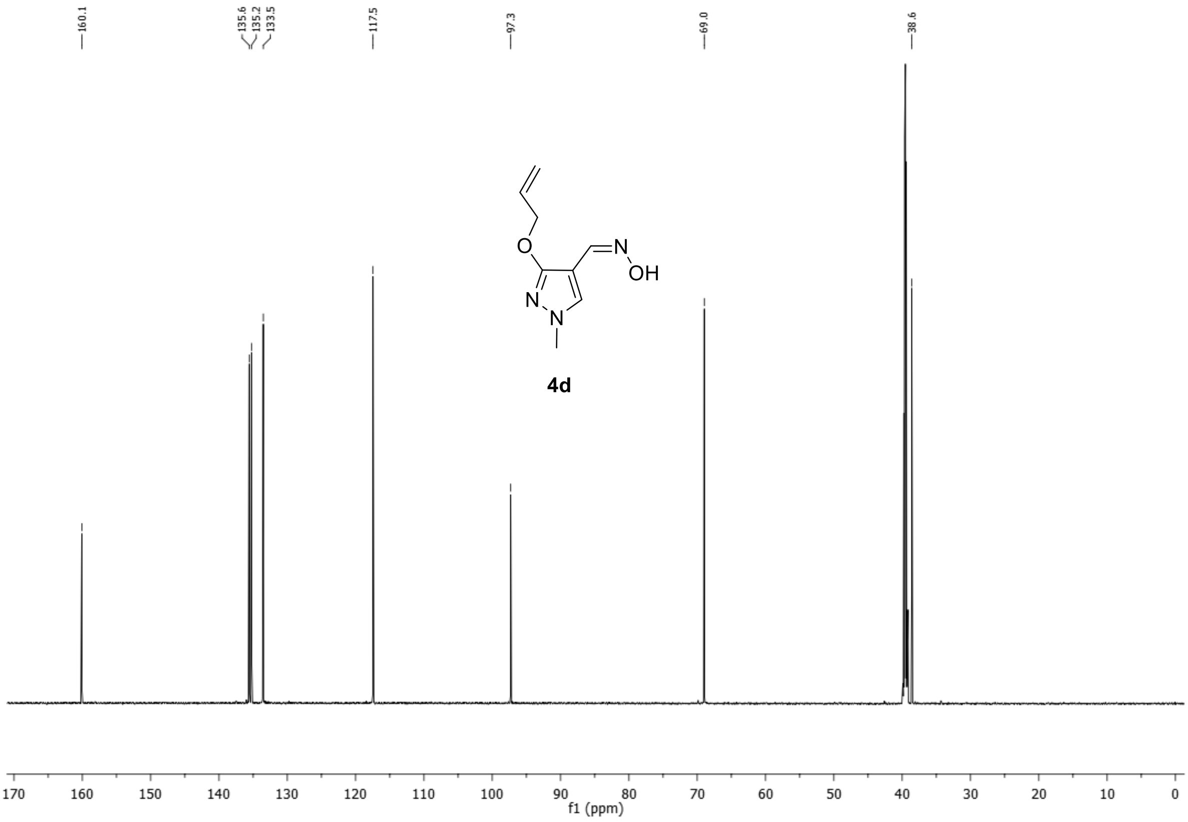

Figure S24. ***N*-[(*Z*)-{1-Methyl-3-[(prop-2-en-1-yl)oxy]-1*H*-pyrazol-4-yl}methylidene]hydroxylamine (4d). ^{13}C NMR spectrum (176 MHz, DMSO-*d*$_6$).**

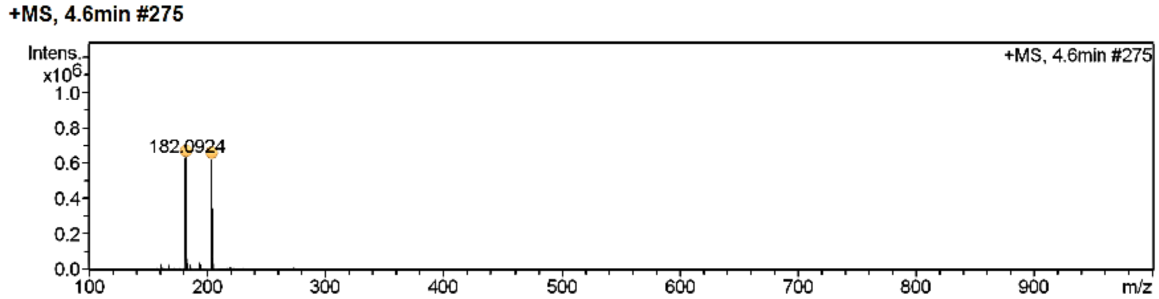

Meas. m/z	#	Ion Formula	m/z	err [ppm]	mSigma	# Sigma	Score	rdb	e⁻ Conf	N-Rule
182.0924	1	C8H12N3O2	182.0924	-0.1	2.7	1	100.00	4.5	even	ok
204.0743	1	C8H11N3NaO2	204.0743	0.1	3.2	1	100.00	4.5	even	ok

Figure S25. ***N*-[(*Z*)-{1-Methyl-3-[(prop-2-en-1-yl)oxy]-1*H*-pyrazol-4-yl}methylidene]hydroxylamine (4d). HRMS (ESI-TOF).**

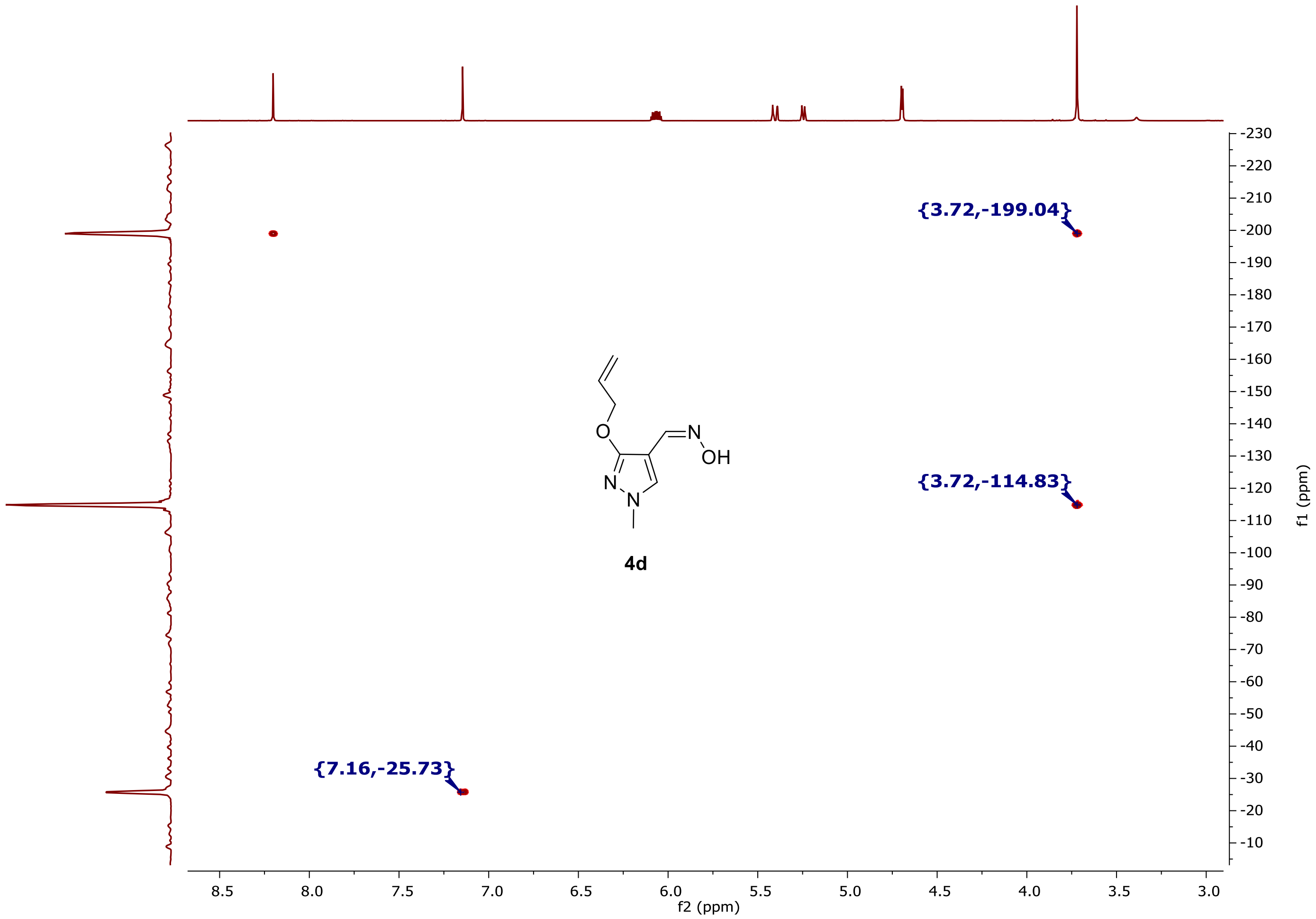

Figure S26. ***N*-[(*Z*)-{1-Methyl-3-[(prop-2-en-1-yl)oxy]-1*H*-pyrazol-4-yl}methylidene]hydroxylamine (4d). ^{1}H-^{15}N HMBC spectrum (71 MHz, DMSO-d_6).**

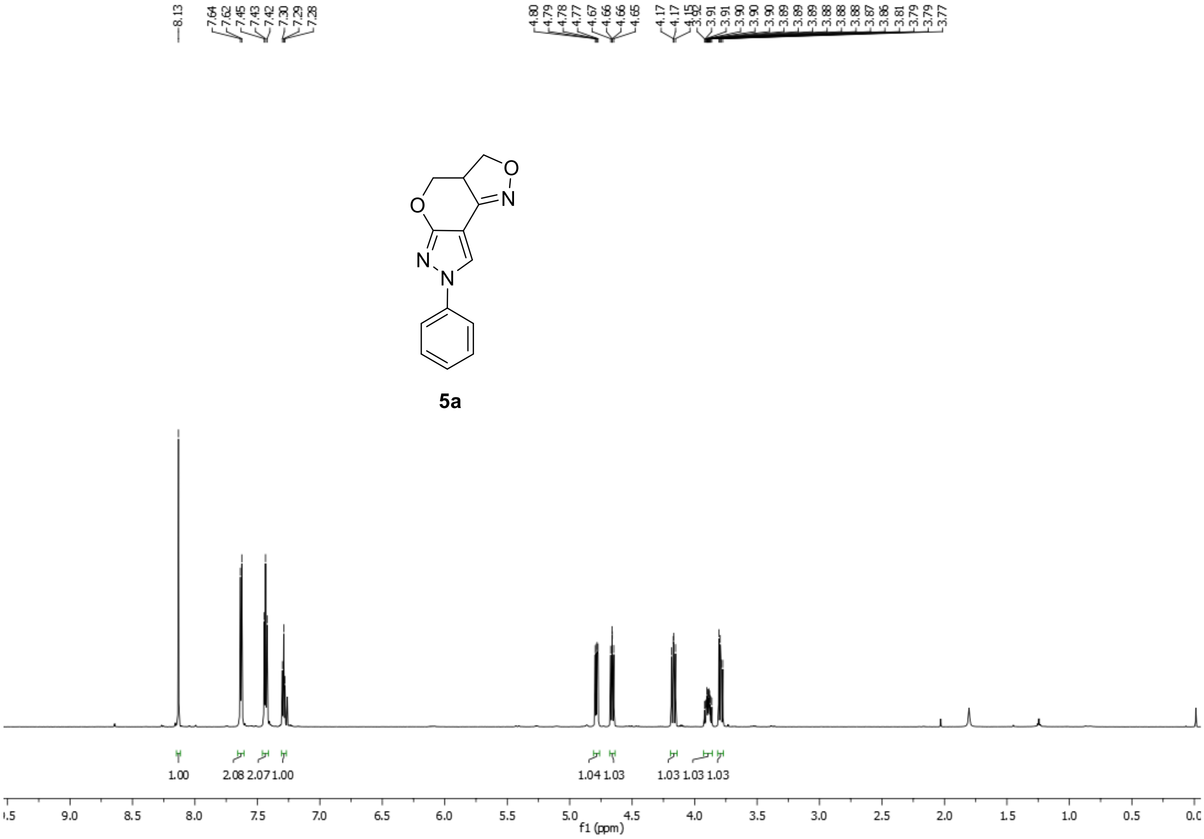

Figure S27. 7-Phenyl-3a,4-dihydro-3*H*,7*H*-pyrazolo[4′,3′:5,6]pyrano[4,3-*c*][1,2]oxazole (5a). ^{1}H NMR spectrum (700 MHz, $CDCl_3$).

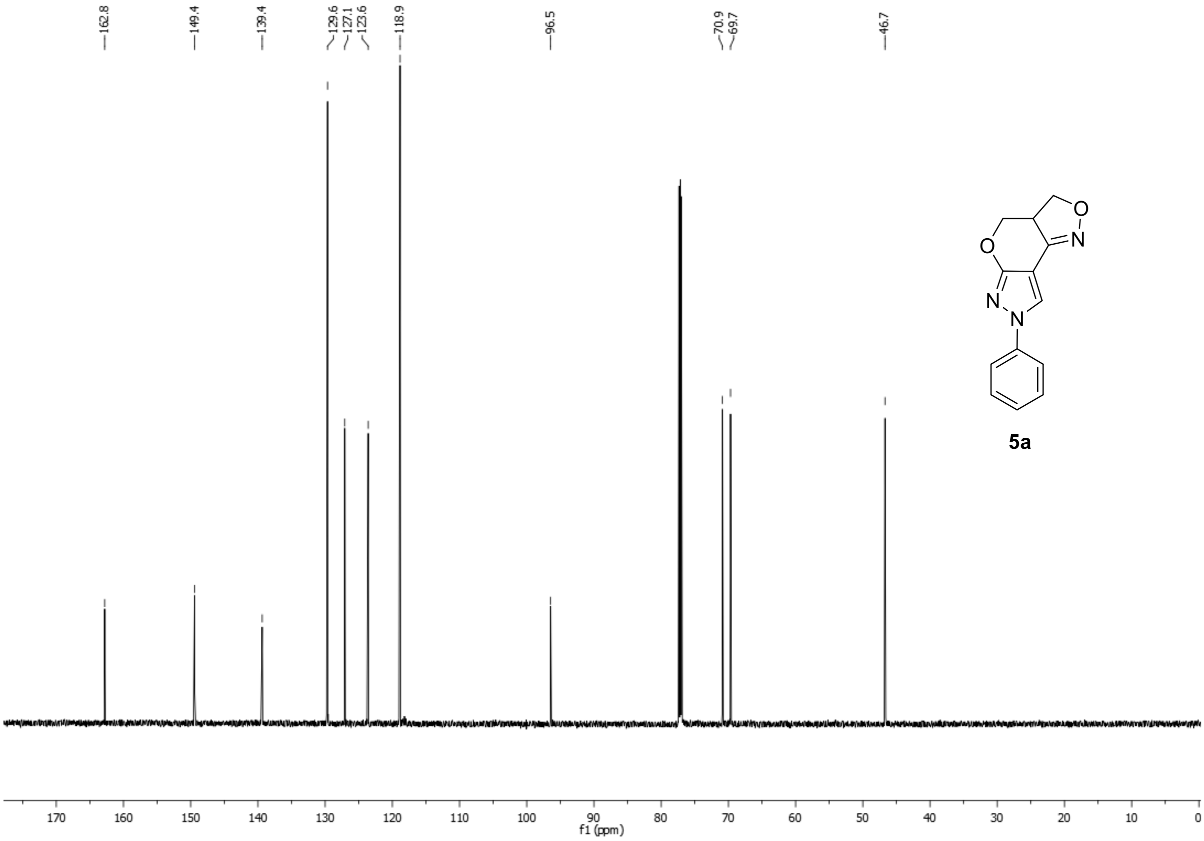

Figure S28. 7-Phenyl-3a,4-dihydro-3*H*,7*H*-pyrazolo[4′,3′:5,6]pyrano[4,3-*c*][1,2]oxazole (5a). ^{13}C NMR spectrum (176 MHz, $CDCl_3$).

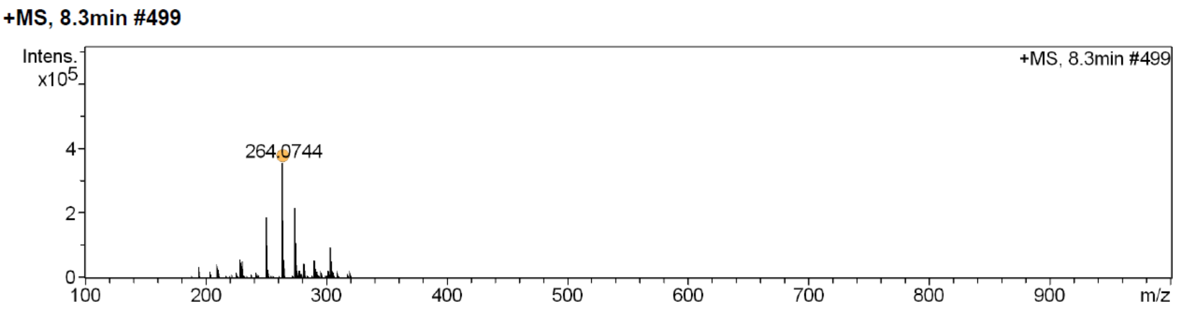

Meas. m/z	#	Ion Formula	m/z	err [ppm]	mSigma	# Sigma	Score	rdb	e¯ Conf	N-Rule
264.0744	1	C13H11N3NaO2	264.0743	-0.2	5.9	1	100.00	9.5	even	ok

Figure S29. 7-Phenyl-3a,4-dihydro-3*H*,7*H*-pyrazolo[4′,3′:5,6]pyrano[4,3-*c*][1,2]oxazole (5a). HRMS (ESI-TOF).

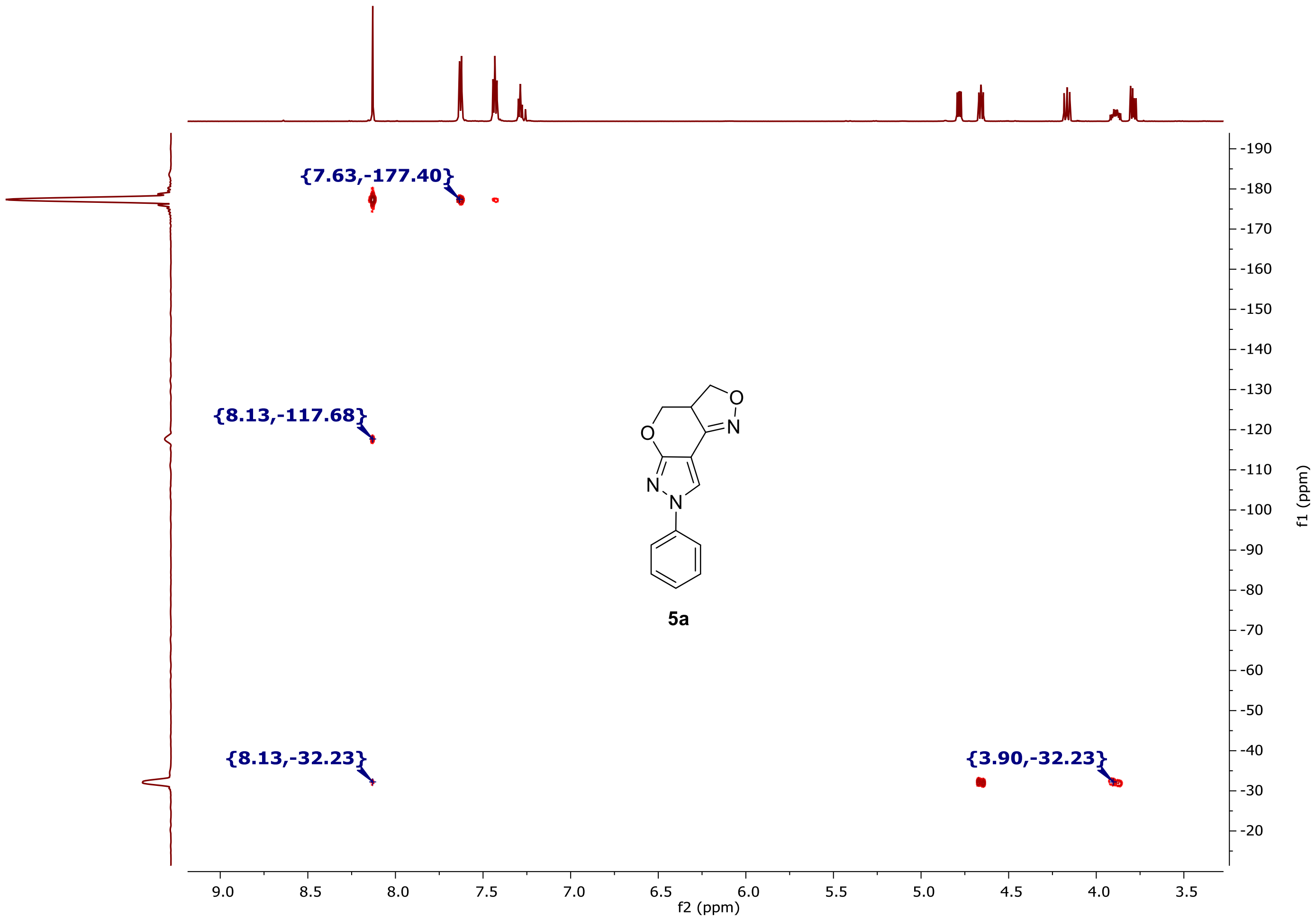

Figure S30. 7-Phenyl-3a,4-dihydro-3*H*,7*H*-pyrazolo[4′,3′:5,6]pyrano[4,3-*c*][1,2]oxazole (5a). ^{1}H-^{15}N HMBC spectrum (71 MHz, $CDCl_3$).

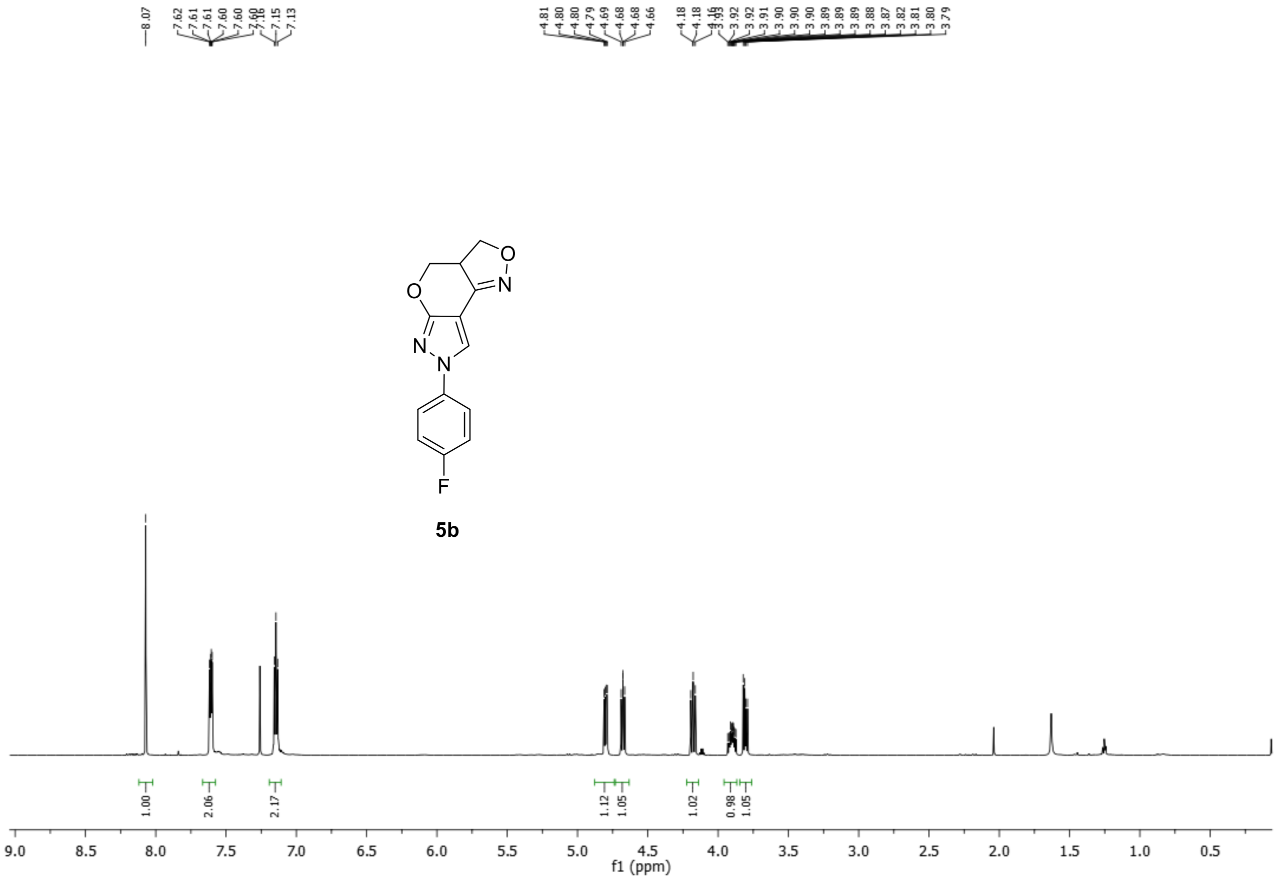

Figure S31. 7-(4-Fluorophenyl)-3a,4-dihydro-3*H*,7*H*-pyrazolo[4′,3′:5,6]pyrano[4,3-*c*][1,2]oxazole (5b). **^{1}H NMR spectrum (700 MHz, $CDCl_3$).**

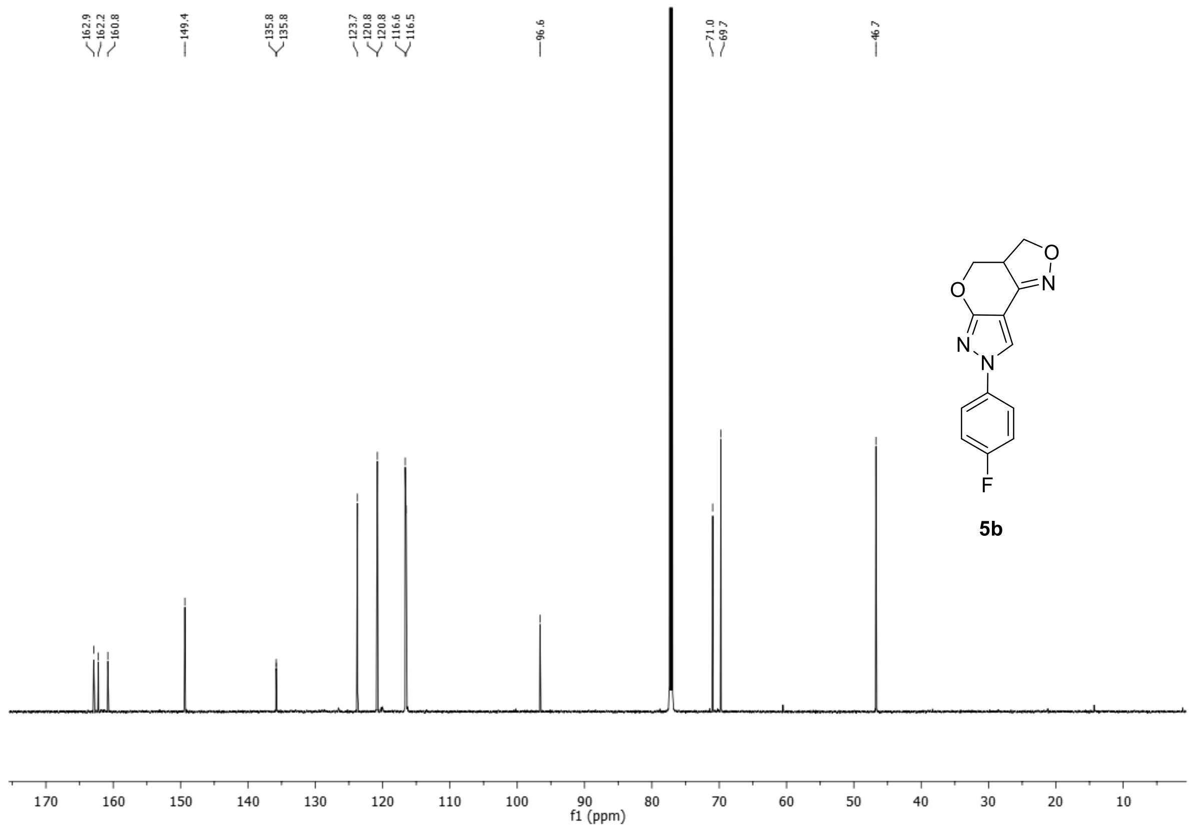

Figure S32. 7-(4-Fluorophenyl)-3a,4-dihydro-3*H*,7*H*-pyrazolo[4′,3′:5,6]pyrano[4,3-*c*][1,2]oxazole (5b). **^{13}C NMR spectrum (176 MHz, $CDCl_3$).**

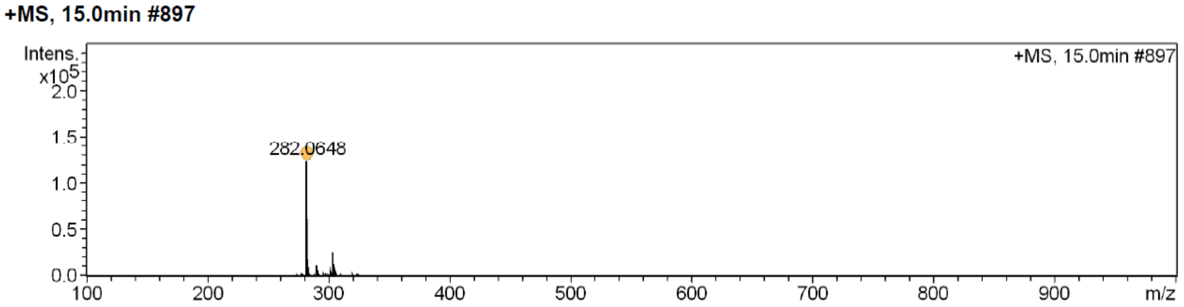

Meas. m/z	#	Ion Formula	m/z	err [ppm]	mSigma	# Sigma	Score	rdb	e⁻ Conf	N-Rule
282.0648	1	C13H10FN3NaO2	282.0649	-0.6	1.7	1	100.00	9.5	even	ok

Figure S34. **7-(4-Fluorophenyl)-3a,4-dihydro-3*H*,7*H*-pyrazolo[4′,3′:5,6]pyrano[4,3-*c*][1,2]oxazole (5b). HRMS (ESI-TOF).**

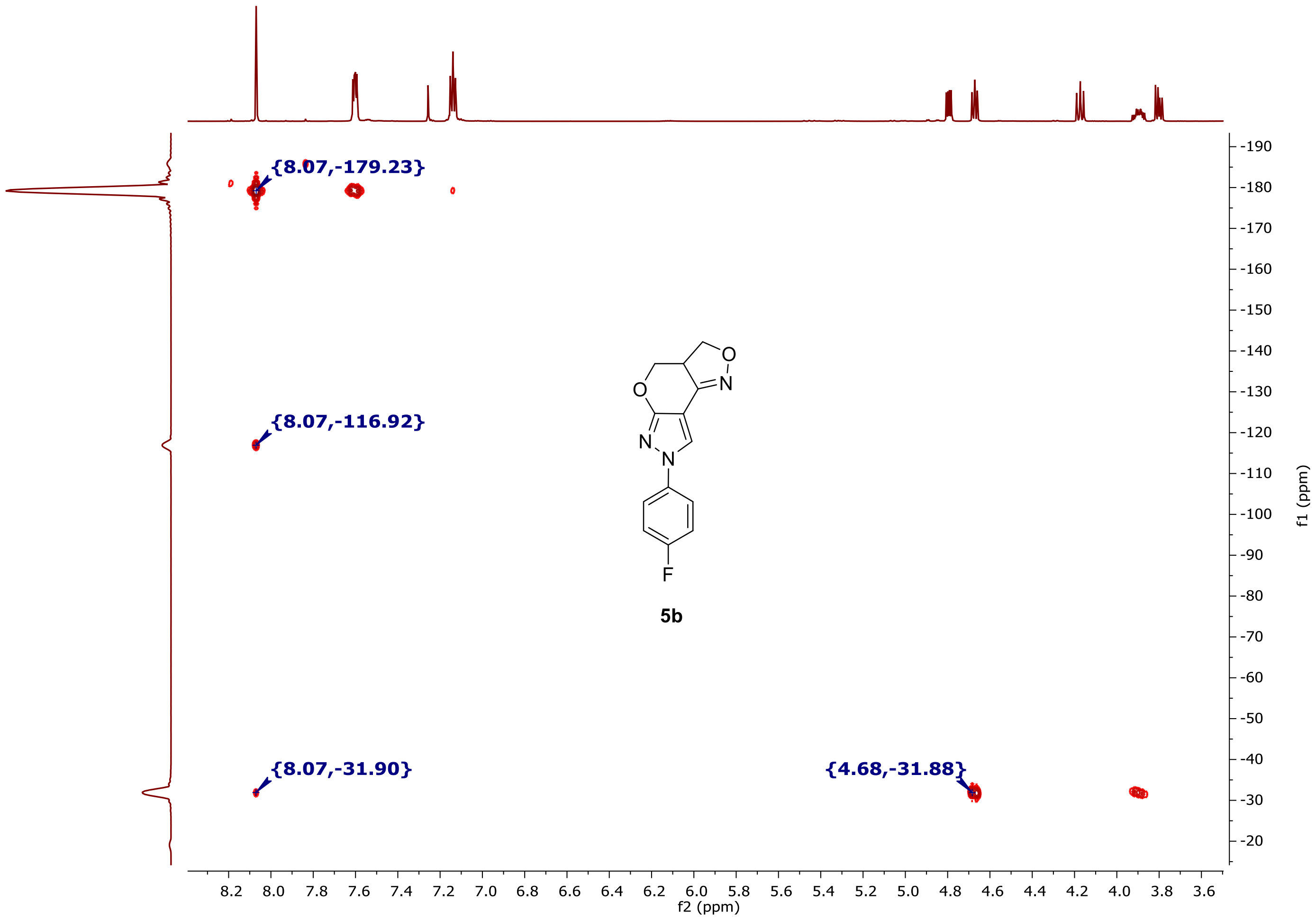

Figure S35. **7-(4-Fluorophenyl)-3a,4-dihydro-3*H*,7*H*-pyrazolo[4′,3′:5,6]pyrano[4,3-*c*][1,2]oxazole (5b). ^{1}H-^{15}N HMBC spectrum (71 MHz, $CDCl_3$).**

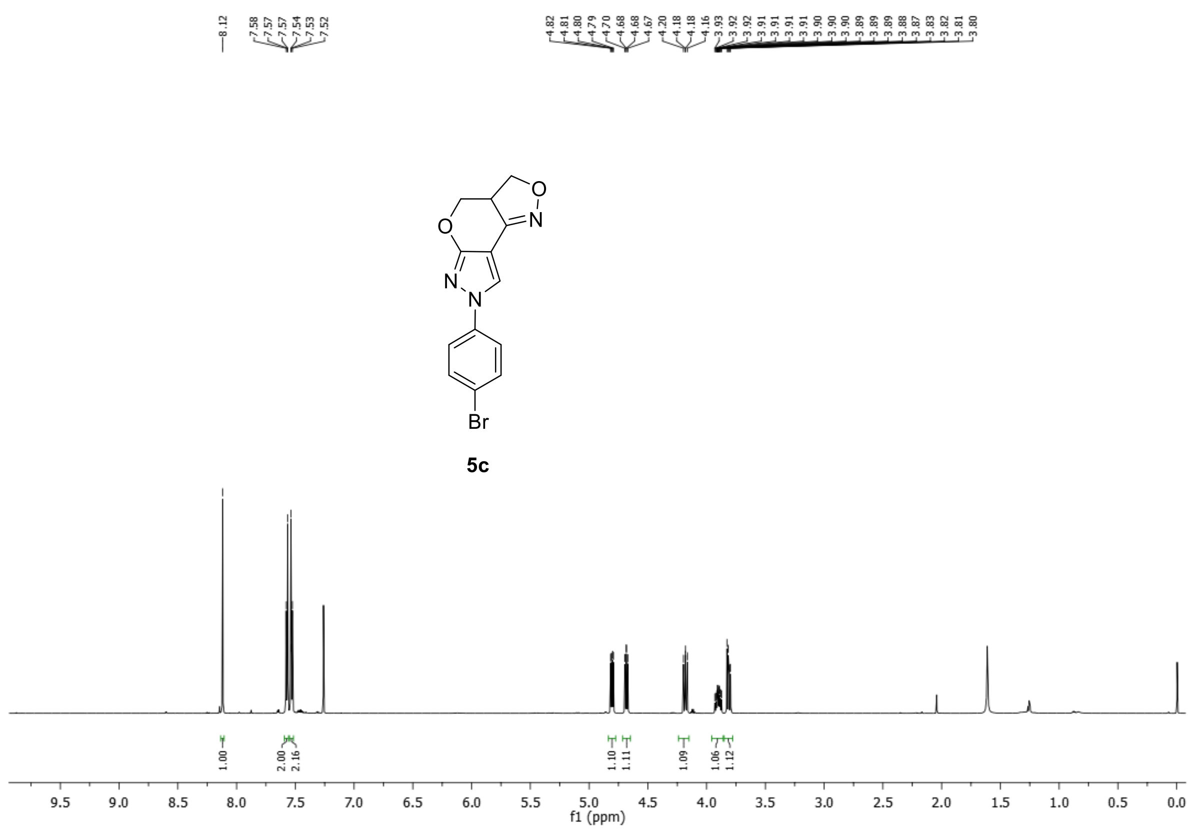

Figure S36. **7-(4-Bromophenyl)-3a,4-dihydro-3*H*,7*H*-pyrazolo[4′,3′:5,6]pyrano[4,3-*c*][1,2]oxazole (5c).** **^{1}H NMR spectrum (700 MHz, $CDCl_3$).**

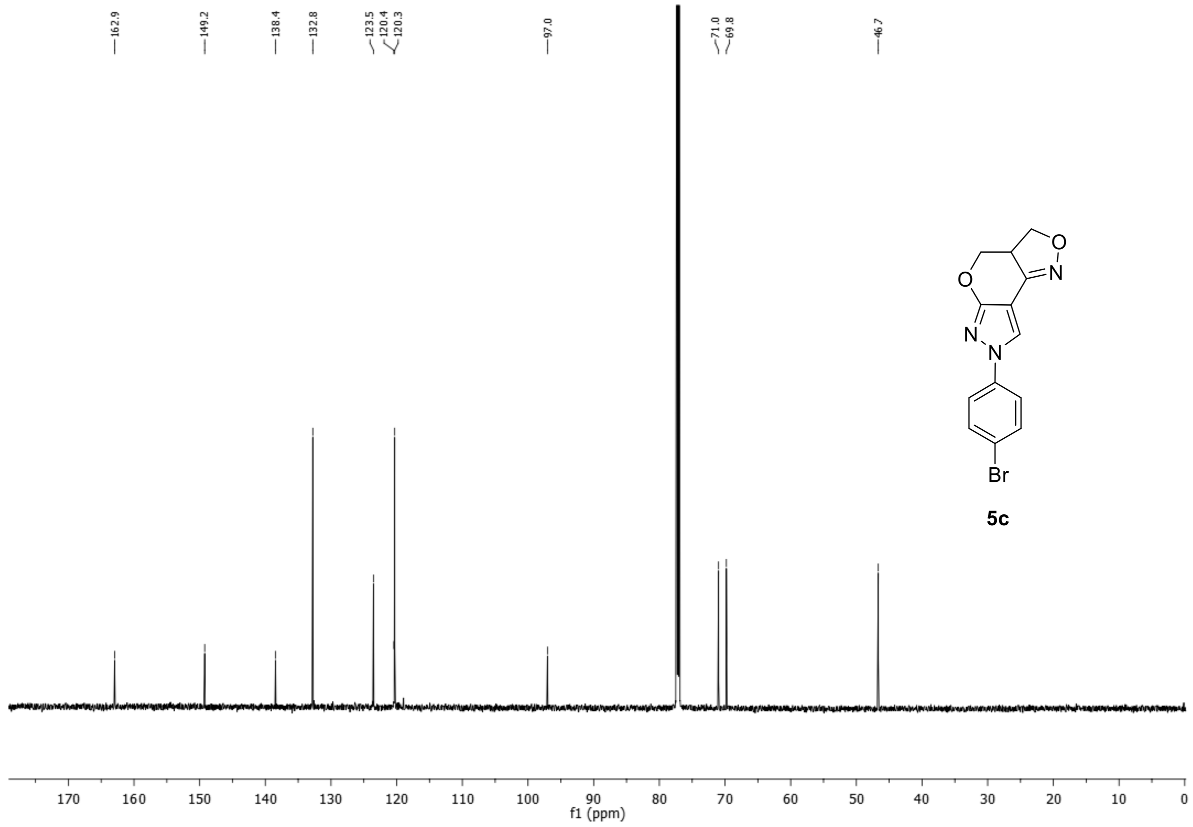

Figure S37. **7-(4-Bromophenyl)-3a,4-dihydro-3*H*,7*H*-pyrazolo[4′,3′:5,6]pyrano[4,3-*c*][1,2]oxazole (5c).** **^{13}C NMR spectrum (176 MHz, $CDCl_3$).**

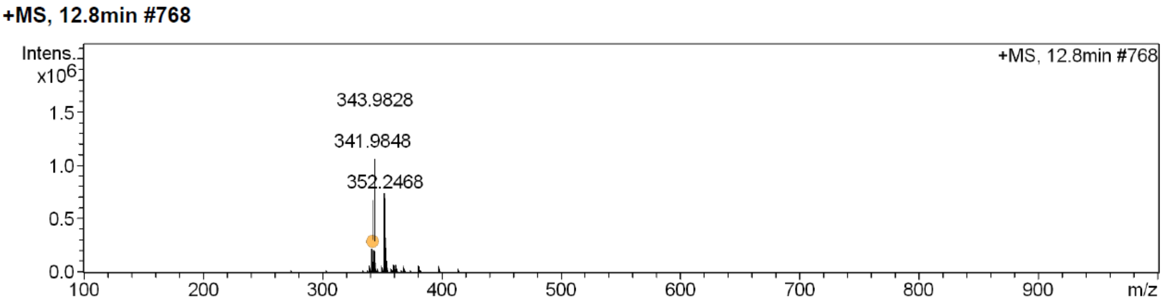

Meas. m/z	#	Ion Formula	m/z	err [ppm]	mSigma	# Sigma	Score	rdb	e⁻ Conf	N-Rule
341.9848	1	C13H10BrN3NaO2	341.9849	0.1	30.9	2	100.00	9.5	even	ok

Figure S38. 7-(4-Bromophenyl)-3a,4-dihydro-3*H*,7*H*-pyrazolo[4′,3′:5,6]pyrano[4,3-*c*][1,2]oxazole (5c). HRMS (ESI-TOF).

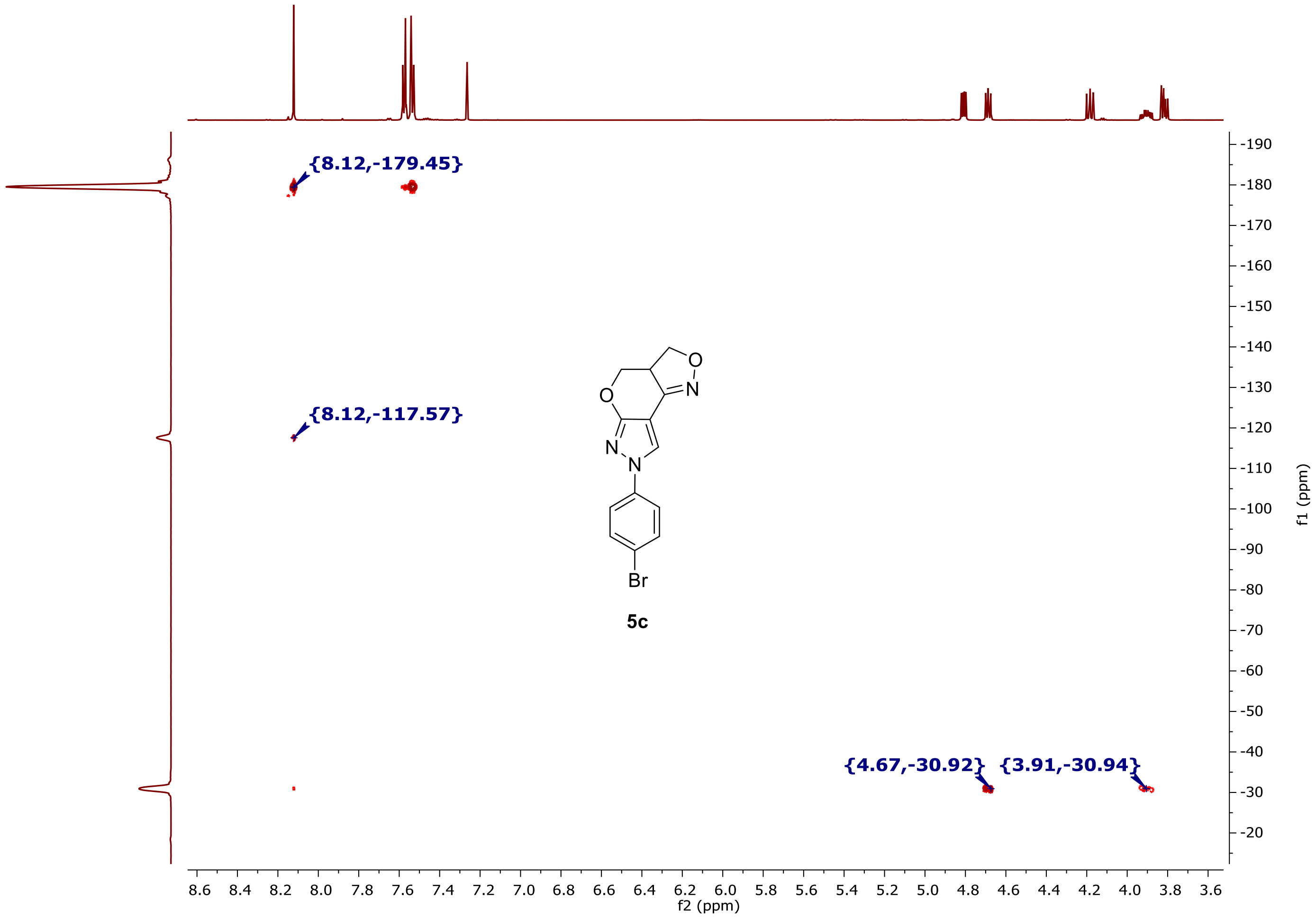

Figure S39. 7-(4-Bromophenyl)-3a,4-dihydro-3*H*,7*H*-pyrazolo[4′,3′:5,6]pyrano[4,3-*c*][1,2]oxazole (5c). ^{1}H-^{15}N HMBC spectrum (71 MHz, $CDCl_3$).

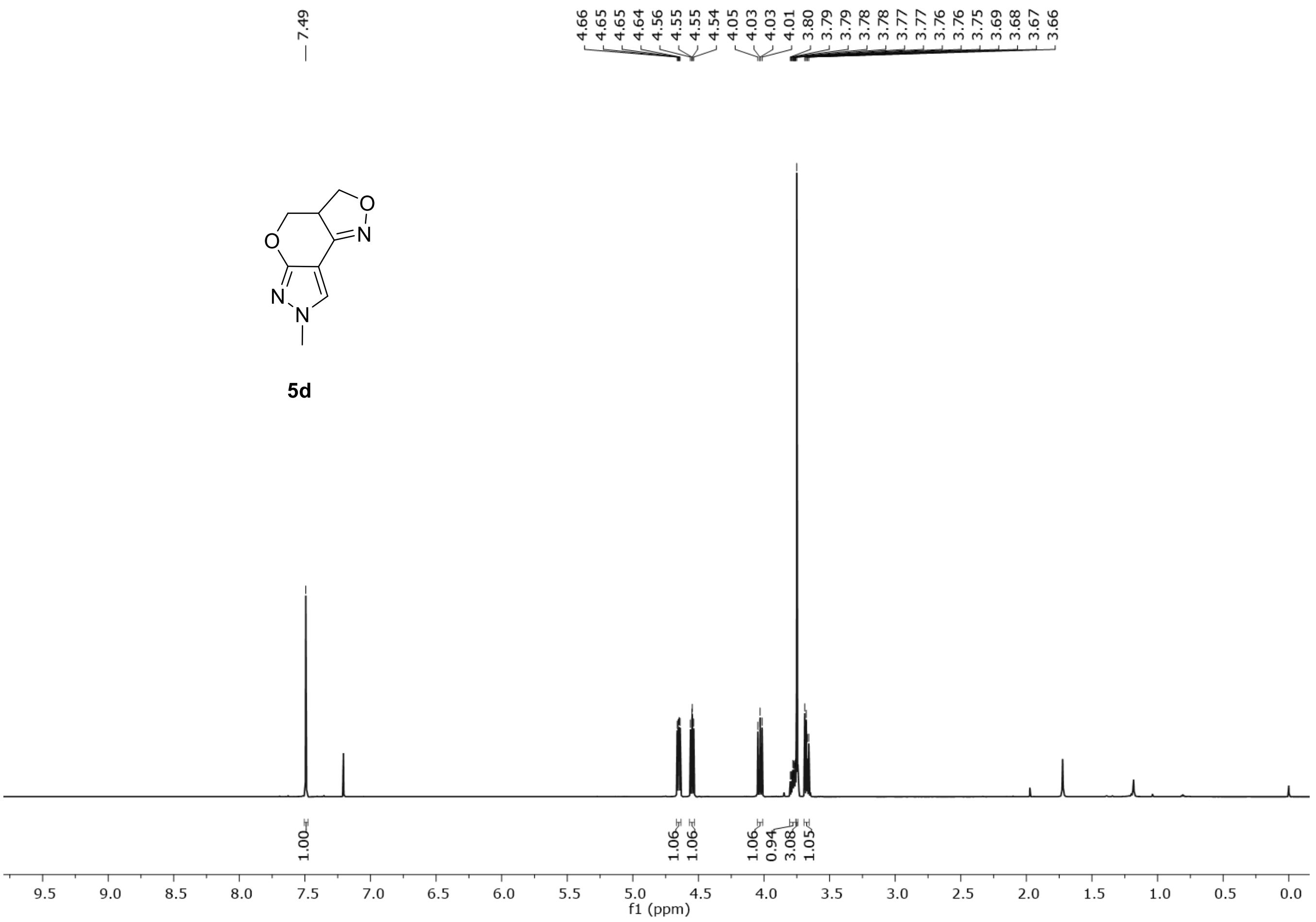

Figure S40. 7-Methyl-3a,4-dihydro-3*H*,7*H*-pyrazolo[4′,3′:5,6]pyrano[4,3-*c*][1,2]oxazole (5d). ^{1}H NMR spectrum (700 MHz, CDCl$_3$).

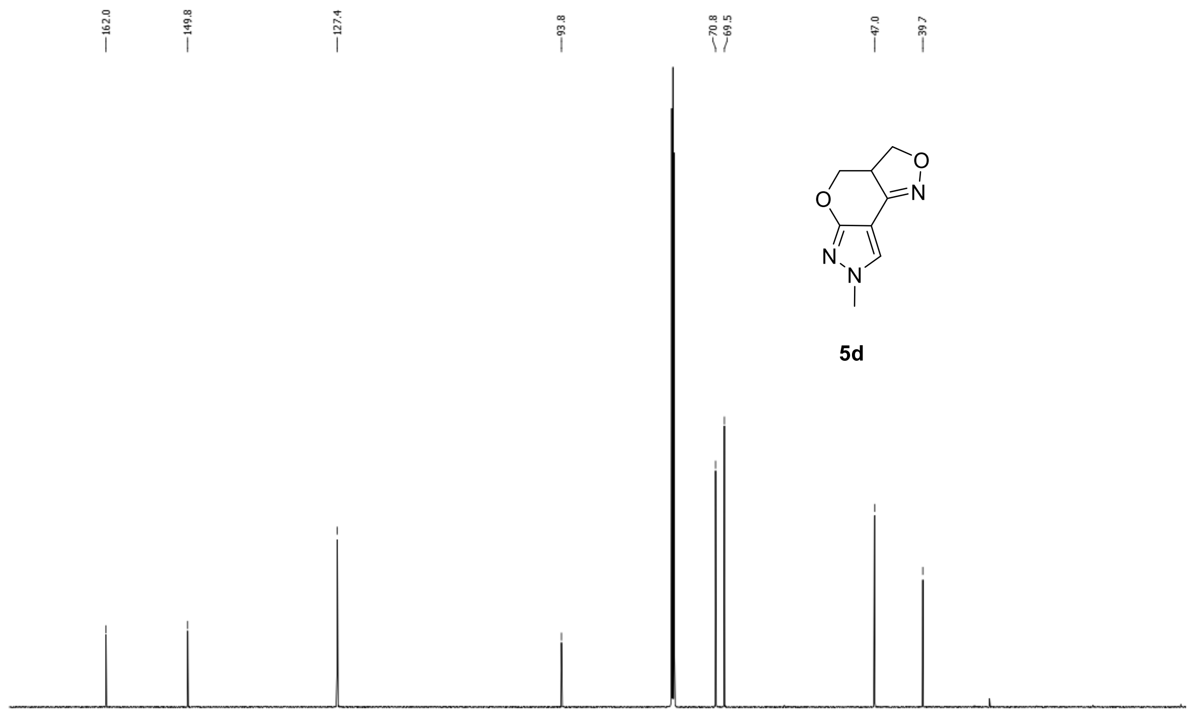

Figure S41. 7-Methyl-3a,4-dihydro-3*H*,7*H*-pyrazolo[4′,3′:5,6]pyrano[4,3-*c*][1,2]oxazole (5d). ^{13}C NMR spectrum (176 MHz, CDCl$_3$).

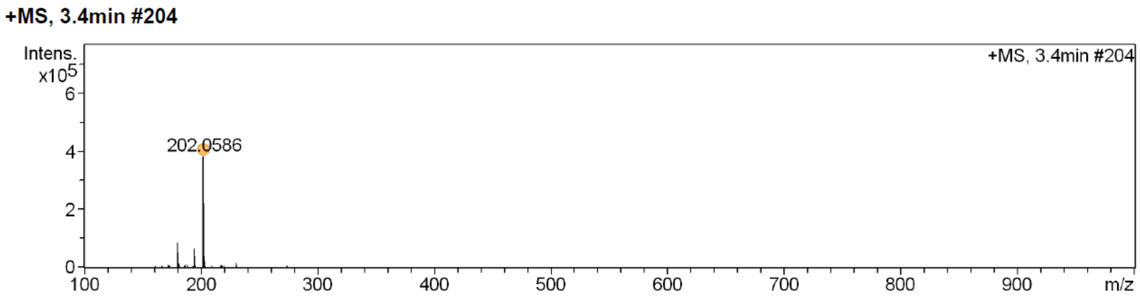

Meas. m/z	#	Ion Formula	m/z	err [ppm]	mSigma	# Sigma	Score	rdb	e⁻ Conf	N-Rule
202.0586	1	C8H9N3NaO2	202.0587	0.6	1.8	1	100.00	5.5	even	ok

Figure S42. 7-Methyl-3a,4-dihydro-3*H*,7*H*-pyrazolo[4′,3′:5,6]pyrano[4,3-*c*][1,2]oxazole (5d). HRMS (ESI-TOF).

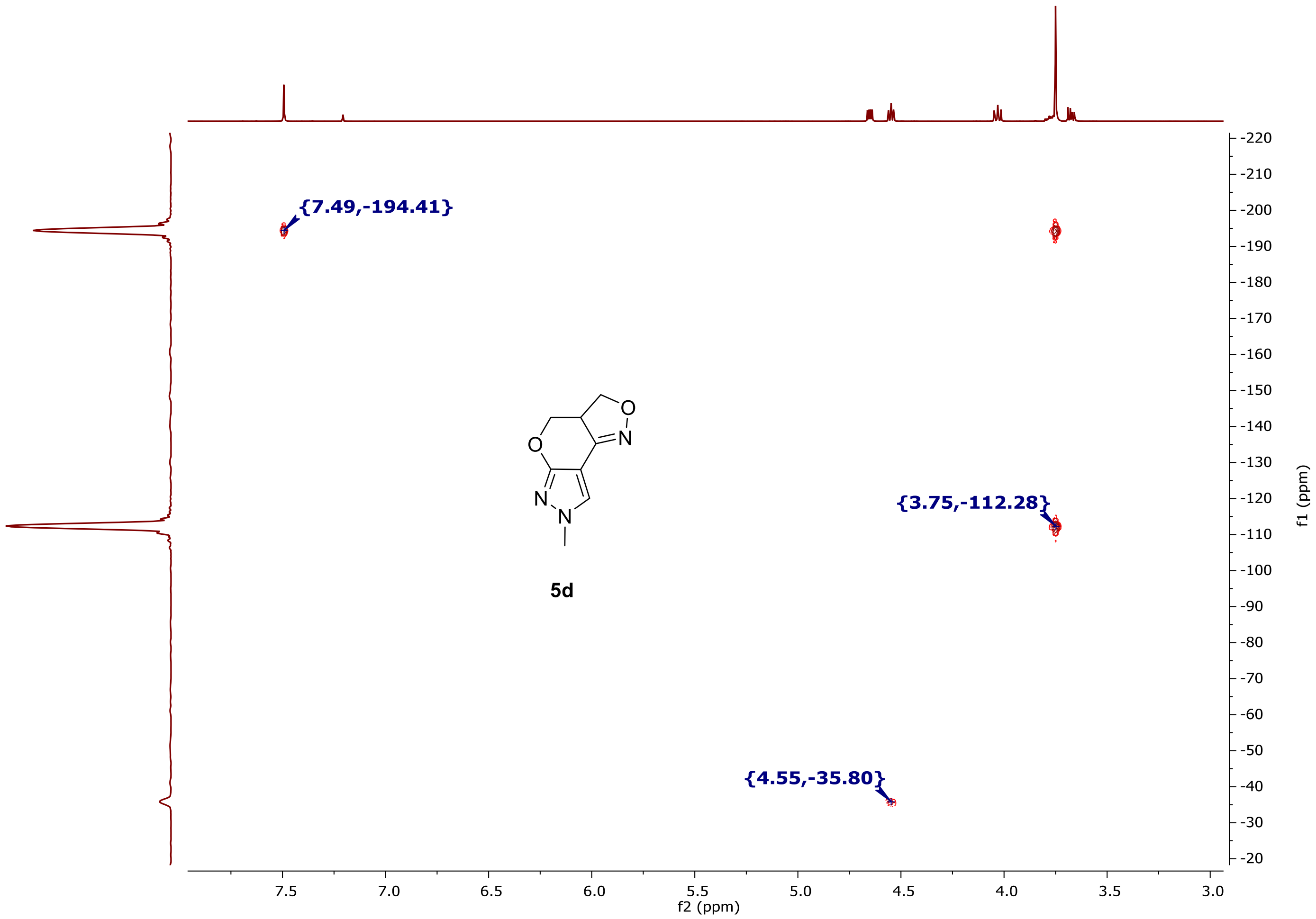

Figure S43. 7-Methyl-3a,4-dihydro-3*H*,7*H*-pyrazolo[4′,3′:5,6]pyrano[4,3-*c*][1,2]oxazole (5d). ^{1}H-^{15}N HMBC spectrum (71 MHz, $CDCl_3$).

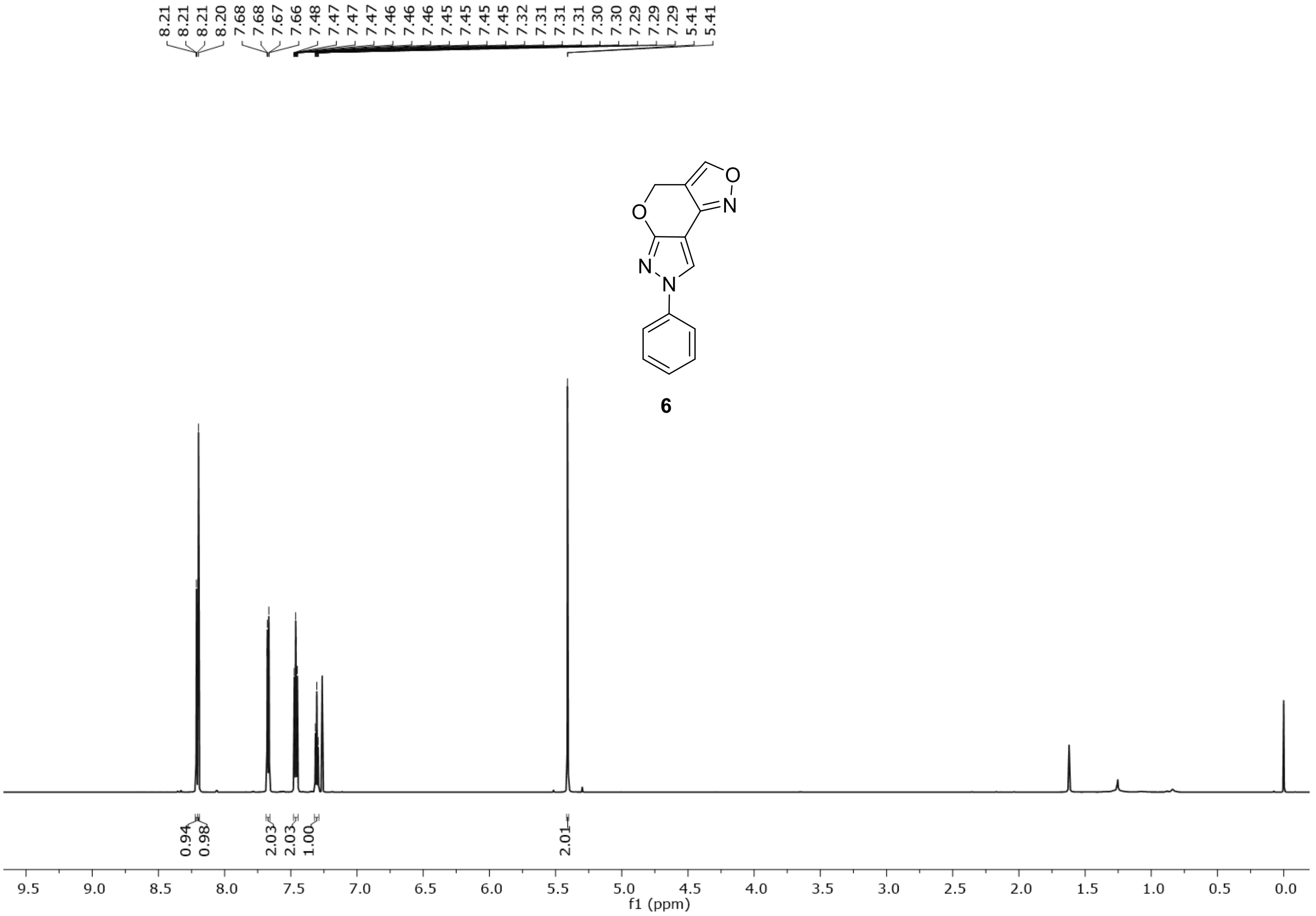

Figure S44. 7-phenyl-4,7-dihydropyrazolo[4',3':5,6]pyrano[4,3-*c*]oxazole (6).· ^{1}H NMR spectrum (700 MHz, $CDCl_3$).

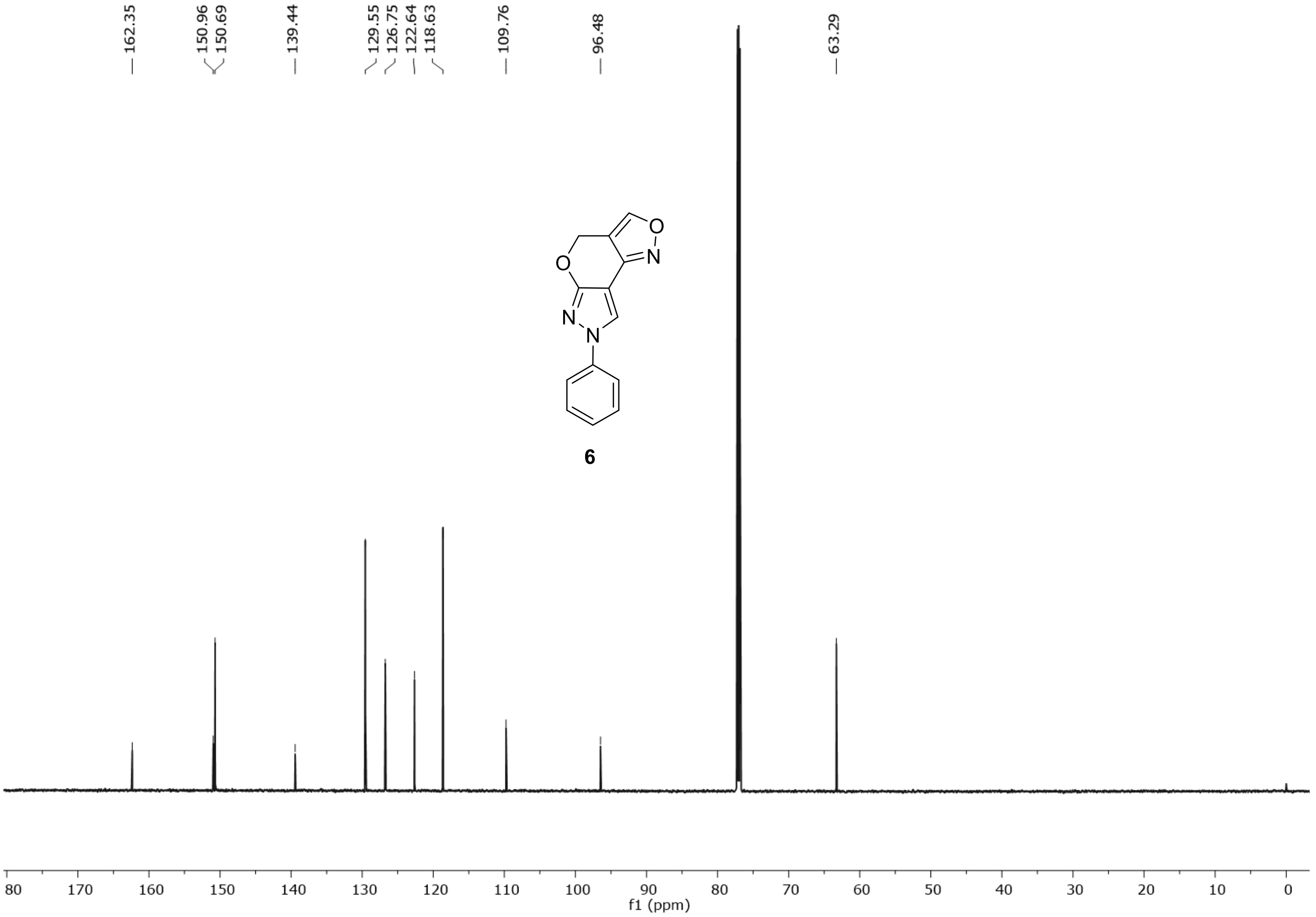

Figure S45. 7-phenyl-4,7-dihydropyrazolo[4',3':5,6]pyrano[4,3-*c*]oxazole (6).· ^{13}C NMR spectrum (176 MHz, $CDCl_3$).

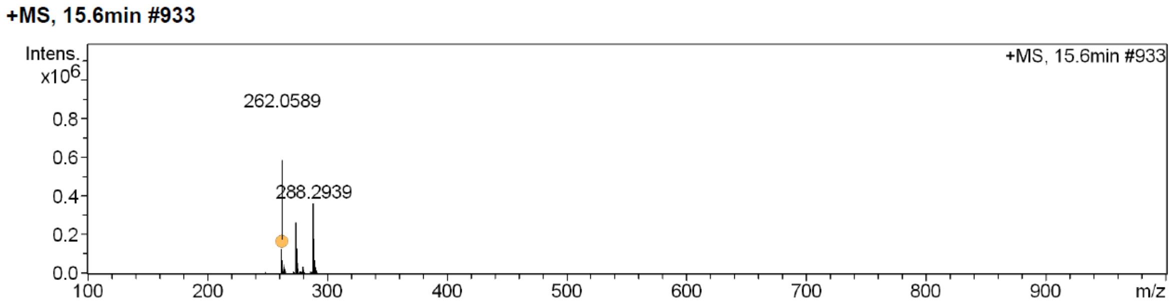

Meas. m/z	#	Ion Formula	m/z	err [ppm]	mSigma	# Sigma	Score	rdb	e⁻ Conf	N-Rule
262.0589	1	C13H9N3NaO2	262.0587	-0.8	10.2	1	100.00	10.5	even	ok

Figure S46. 7-Phenyl-4,7-dihydropyrazolo[4',3':5,6]pyrano[4,3-*c*]oxazole (6).·HRMS (ESI-TOF).

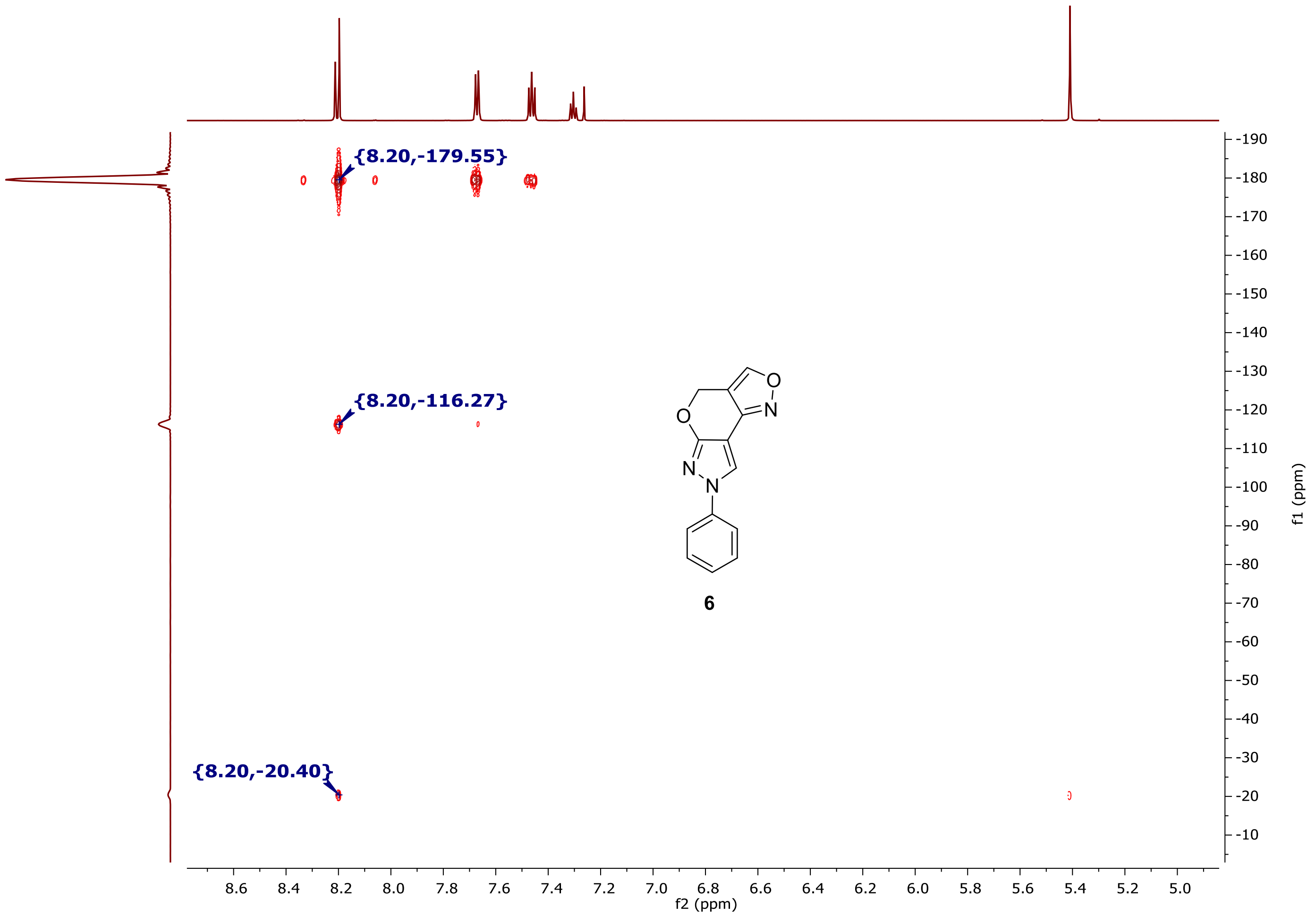

Figure S47. 7-Phenyl-4,7-dihydropyrazolo[4',3':5,6]pyrano[4,3-*c*]oxazole (6).· ^{1}H-^{15}N HMBC spectrum (71 MHz, $CDCl_3$).

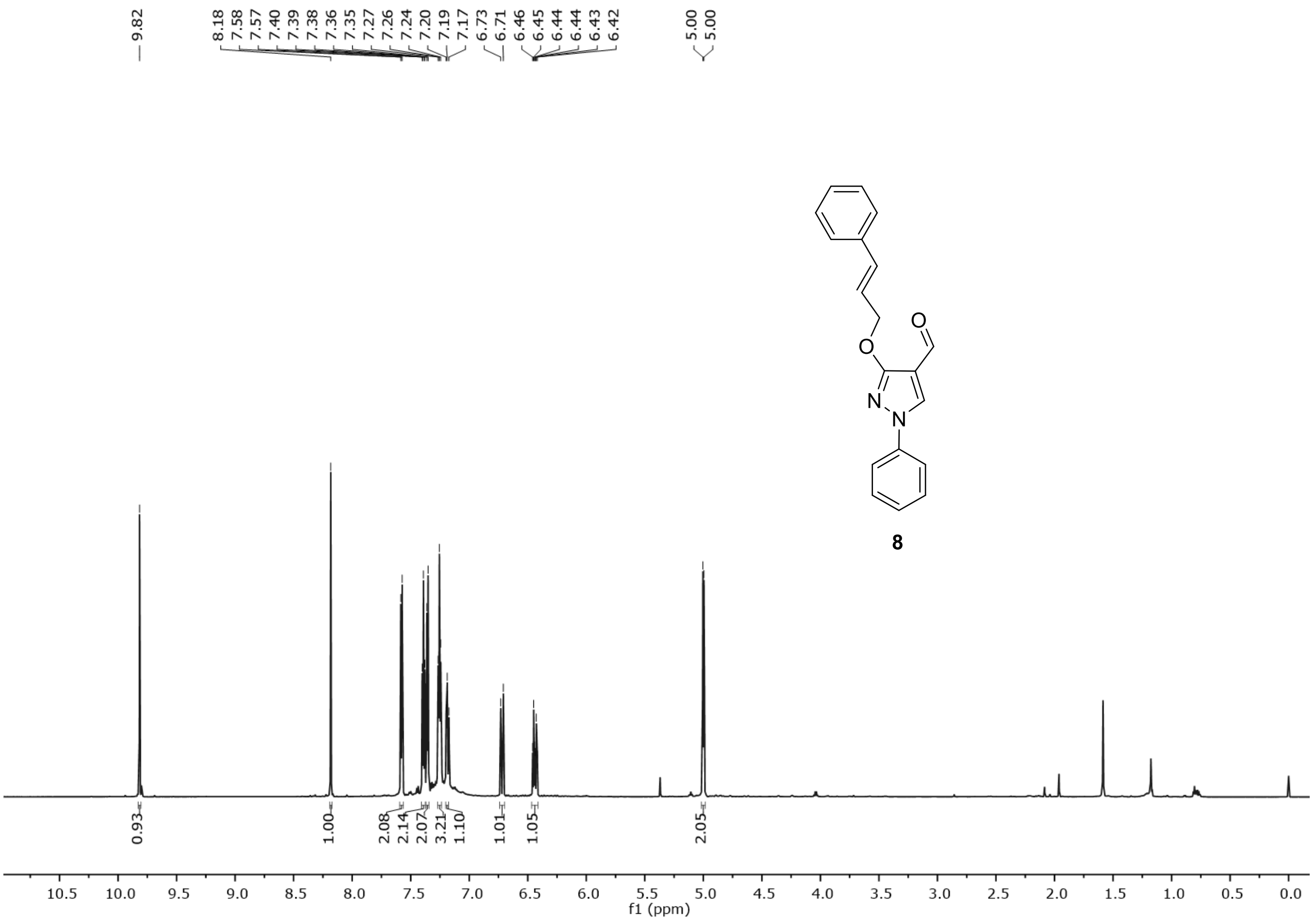

Figure S48. 1-Phenyl-3-{[(2*Z*)-3-phenylprop-2-en-1-yl]oxy}-1*H*-pyrazole-4-carbaldehyde (8).^{1}H NMR spectrum (700 MHz, $CDCl_3$).

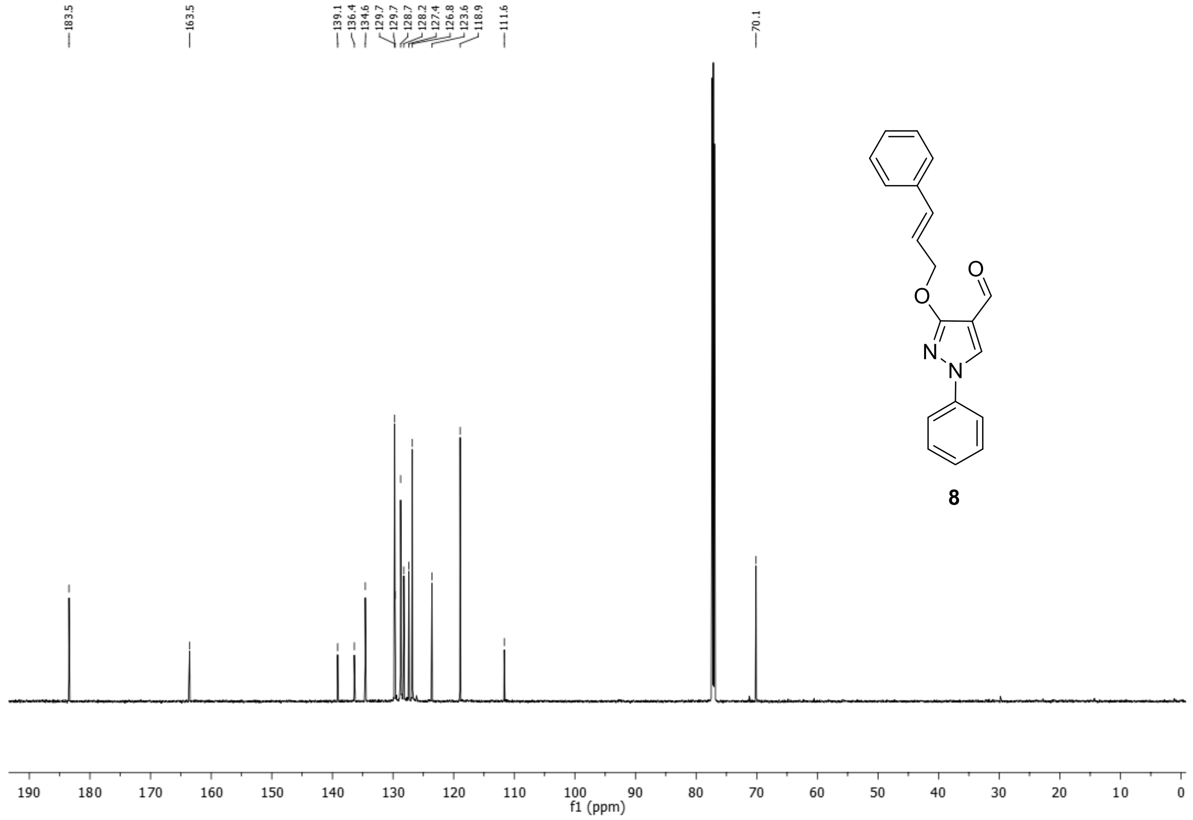

Figure S49. 1-Phenyl-3-{[(2*Z*)-3-phenylprop-2-en-1-yl]oxy}-1*H*-pyrazole-4-carbaldehyde (8).^{13}C NMR spectrum (176 MHz, $CDCl_3$).

+MS, 5.5min #329

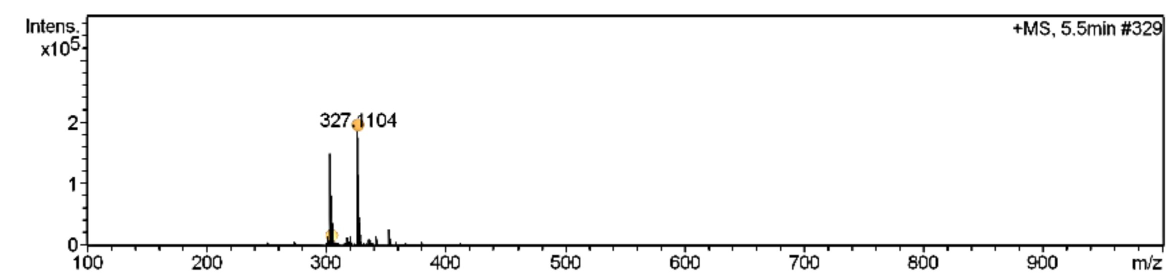

Meas. m/z	#	Ion Formula	m/z	err [ppm]	mSigma	# Sigma	Score	rdb	e⁻ Conf	N-Rule
305.1285	1	C19H17N2O2	305.1285	-0.2	206.9	1	100.00	12.5	even	ok
327.1104	1	C19H16N2NaO2	327.1104	0.1	3.6	1	100.00	12.5	even	ok

Figure S50. 1-Phenyl-3-{[(2*Z*)-3-phenylprop-2-en-1-yl]oxy}-1*H*-pyrazole-4-carbaldehyde (8). HRMS (ESI-TOF).

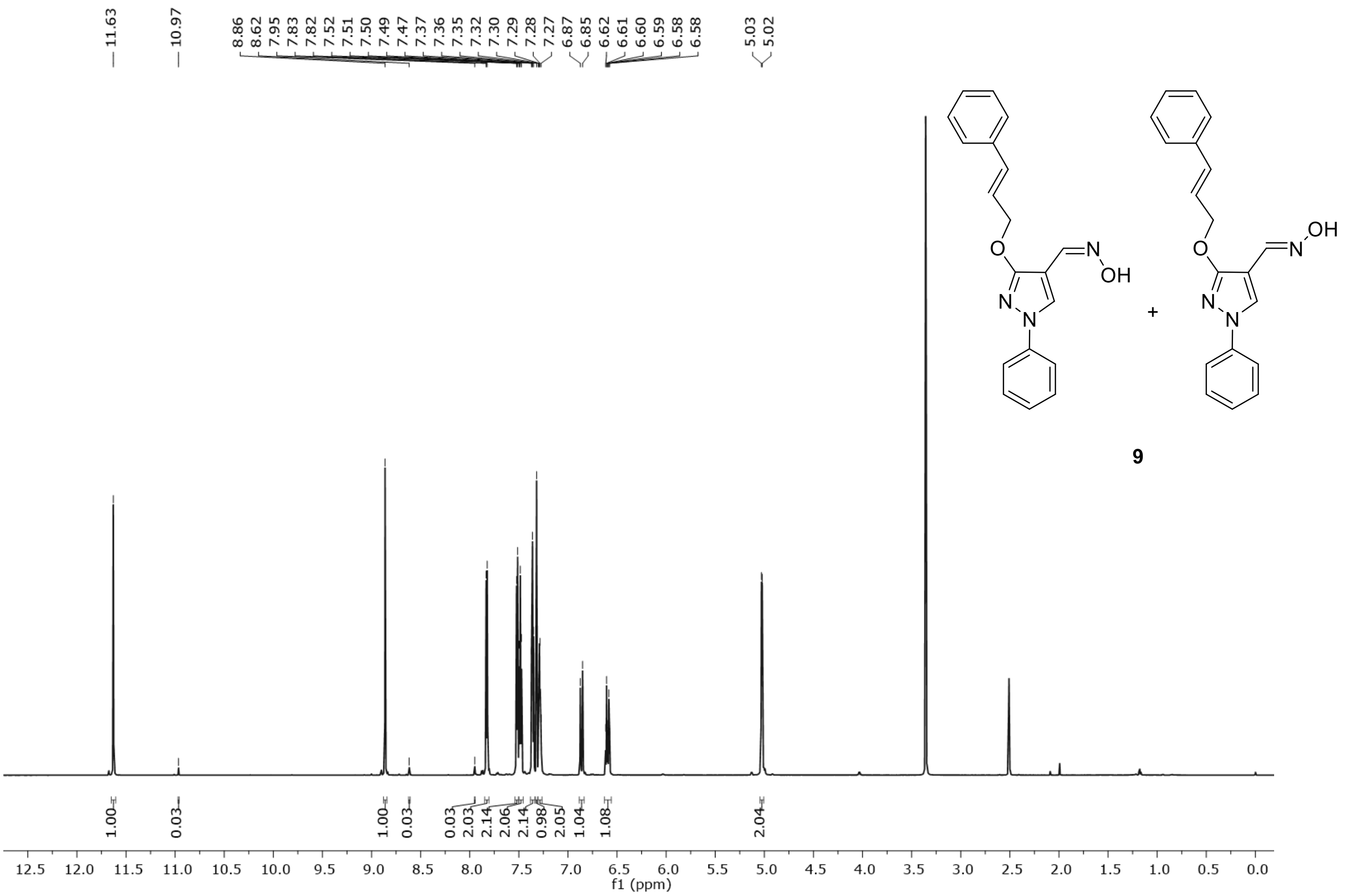

Figure S51. *N*-[(Z/*E*)-{1-Phenyl-3-{[(2*Z*)-3-phenylprop-2-en-1-yl)oxy]-1*H*-pyrazol-4-yl}methylidene]hydroxylamine (9). ^{1}H NMR spectrum (700 MHz, DMSO-d_6).

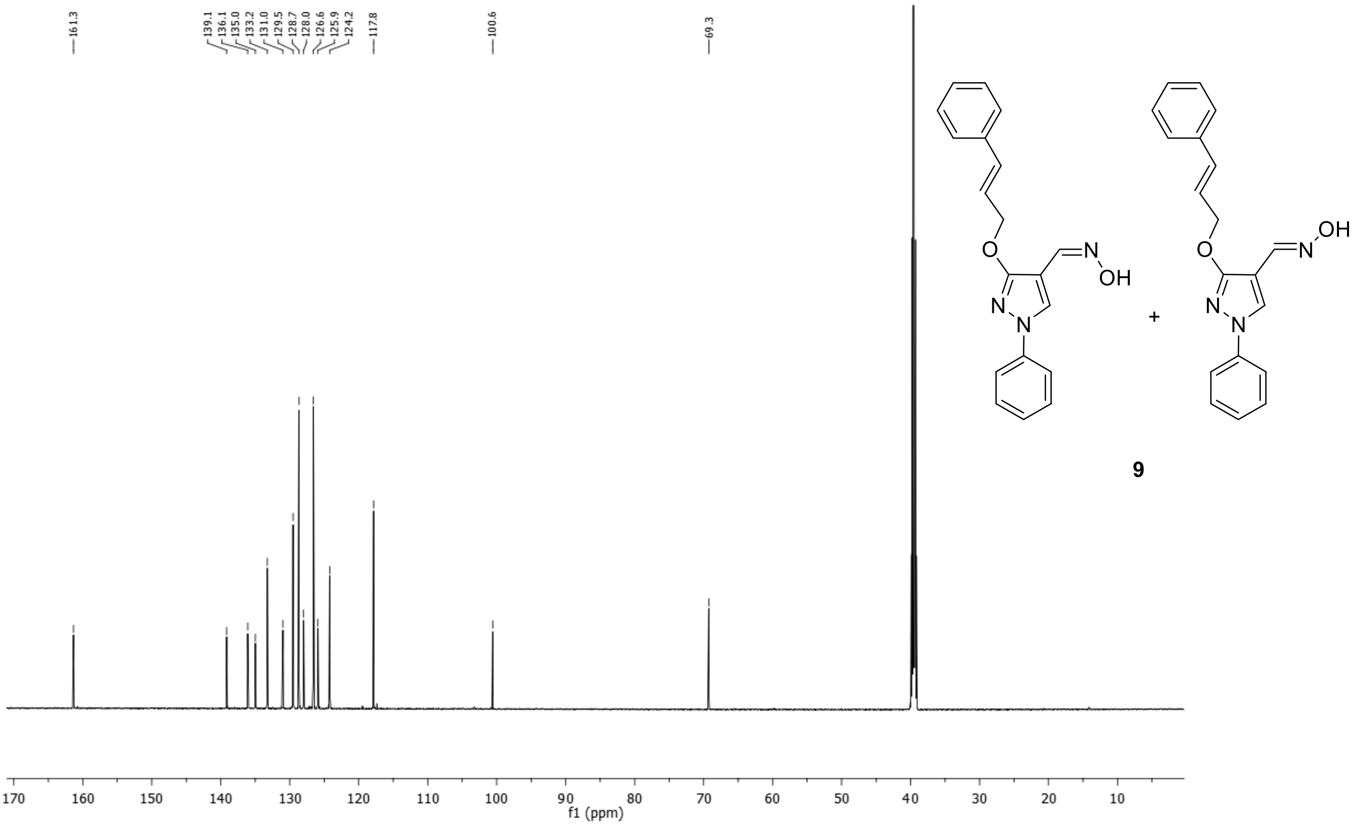

Figure S52. ***N*-[(Z/*E*)-{1-Phenyl-3-{[(2*Z*)-3-phenylprop-2-en-1-yl)oxy]-1*H*-pyrazol-4-yl}methylidene]hydroxylamine (9).** ^{13}**C NMR spectrum (176 MHz, DMSO-*d*$_6$).**

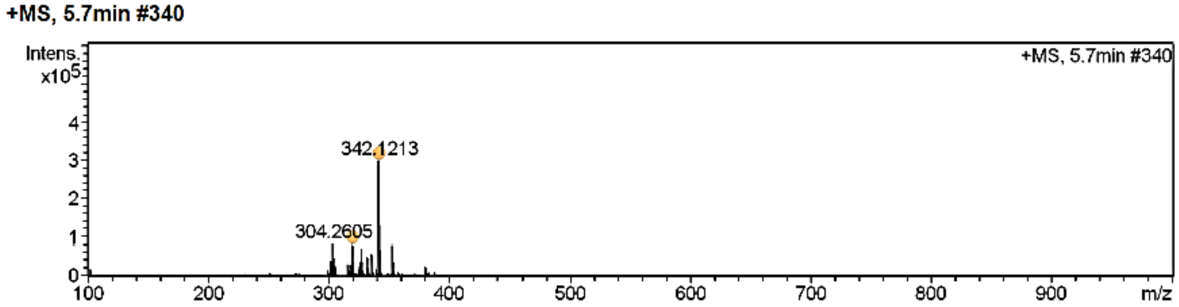

Meas. m/z	#	Ion Formula	m/z	err [ppm]	mSigma	# Sigma	Score	rdb	e⁻ Conf	N-Rule
320.1389	1	C19H18N3O2	320.1394	-1.4	13.5	1	100.00	12.5	even	ok
342.1213	1	C19H17N3NaO2	342.1213	-0.1	13.5	1	100.00	12.5	even	ok

Figure S53. ***N*-[(Z/*E*)-{1-Phenyl-3-{[(2*Z*)-3-phenylprop-2-en-1-yl)oxy]-1*H*-pyrazol-4-yl}methylidene]hydroxylamine (9). HRMS (ESI-TOF).**

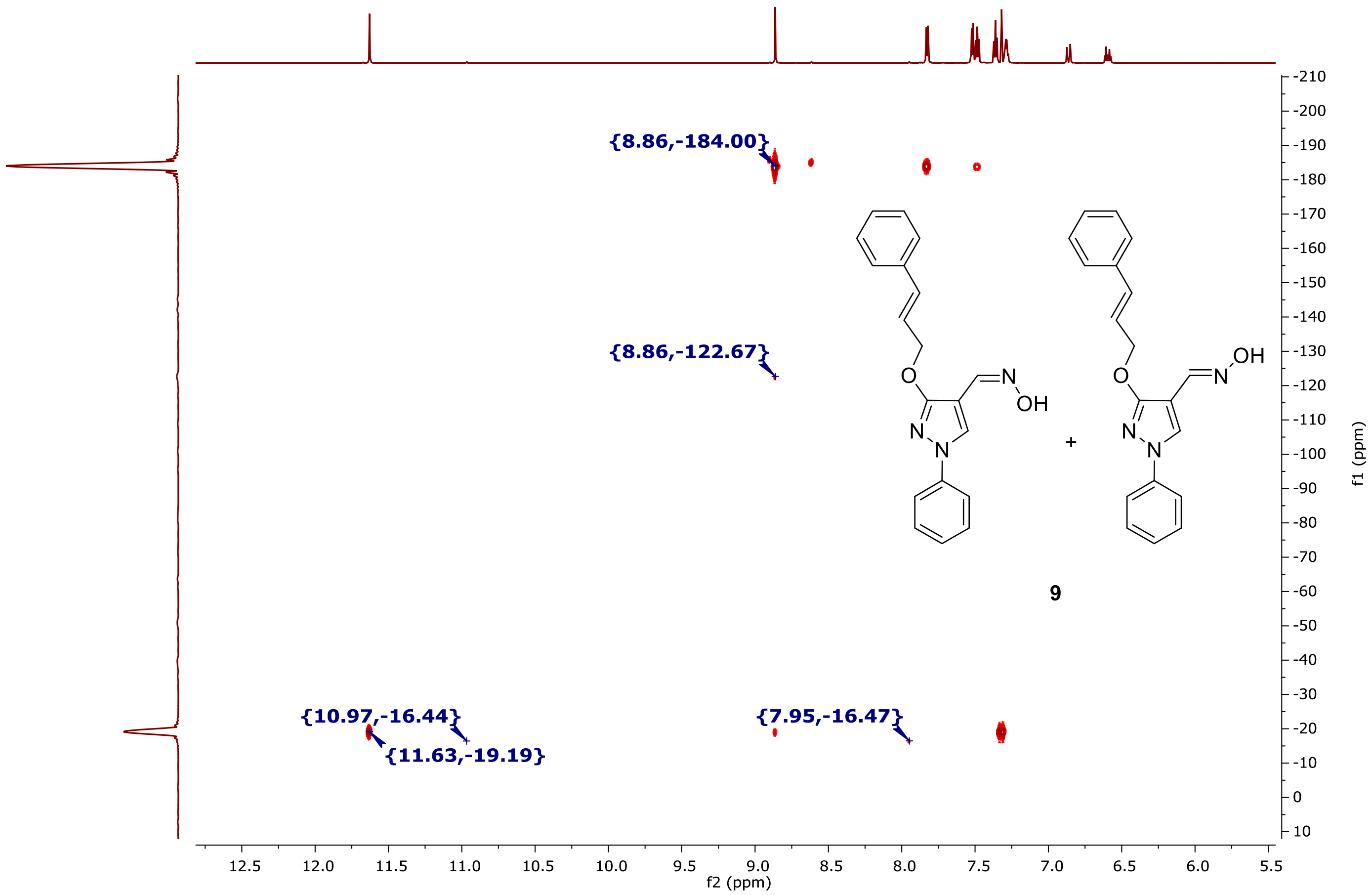

Figure S54. ***N*-[(Z/*E*)-{1-Phenyl-3-{[(2*Z*)-3-phenylprop-2-en-1-yl)oxy]-1*H*-pyrazol-4-yl}methylidene]hydroxylamine (9). ^{1}H-^{15}N HMBC spectrum (700 MHz, DMSO-*d*$_6$).**

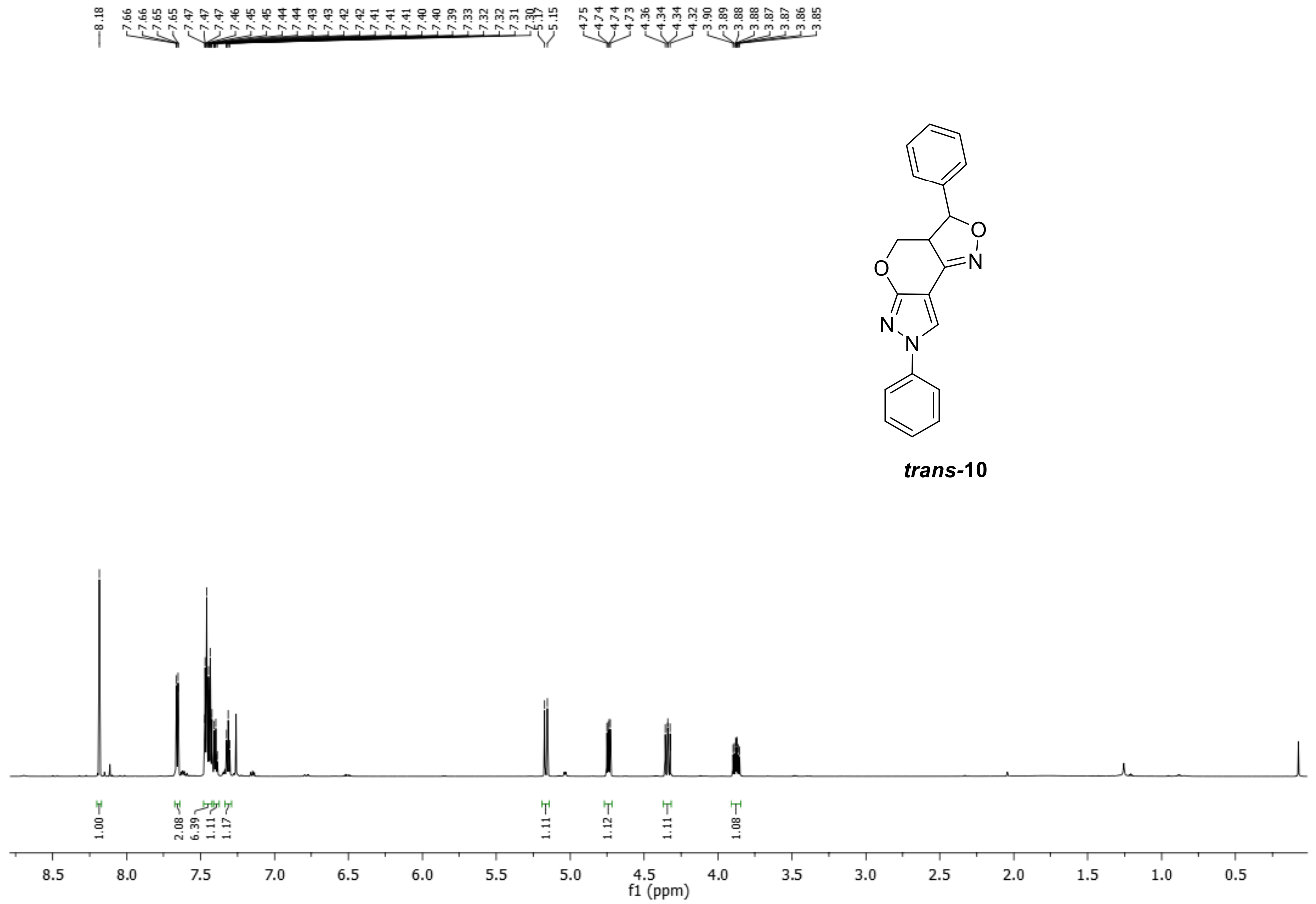

Figure S55. 3,7-Diphenyl-3a,4-dihydro-3*H*,7*H*-pyrazolo[4′,3′:5,6]pyrano[4,3-*c*][1,2]oxazole (*trans*-10).^{1}H NMR spectrum (700 MHz, CDCl$_3$).

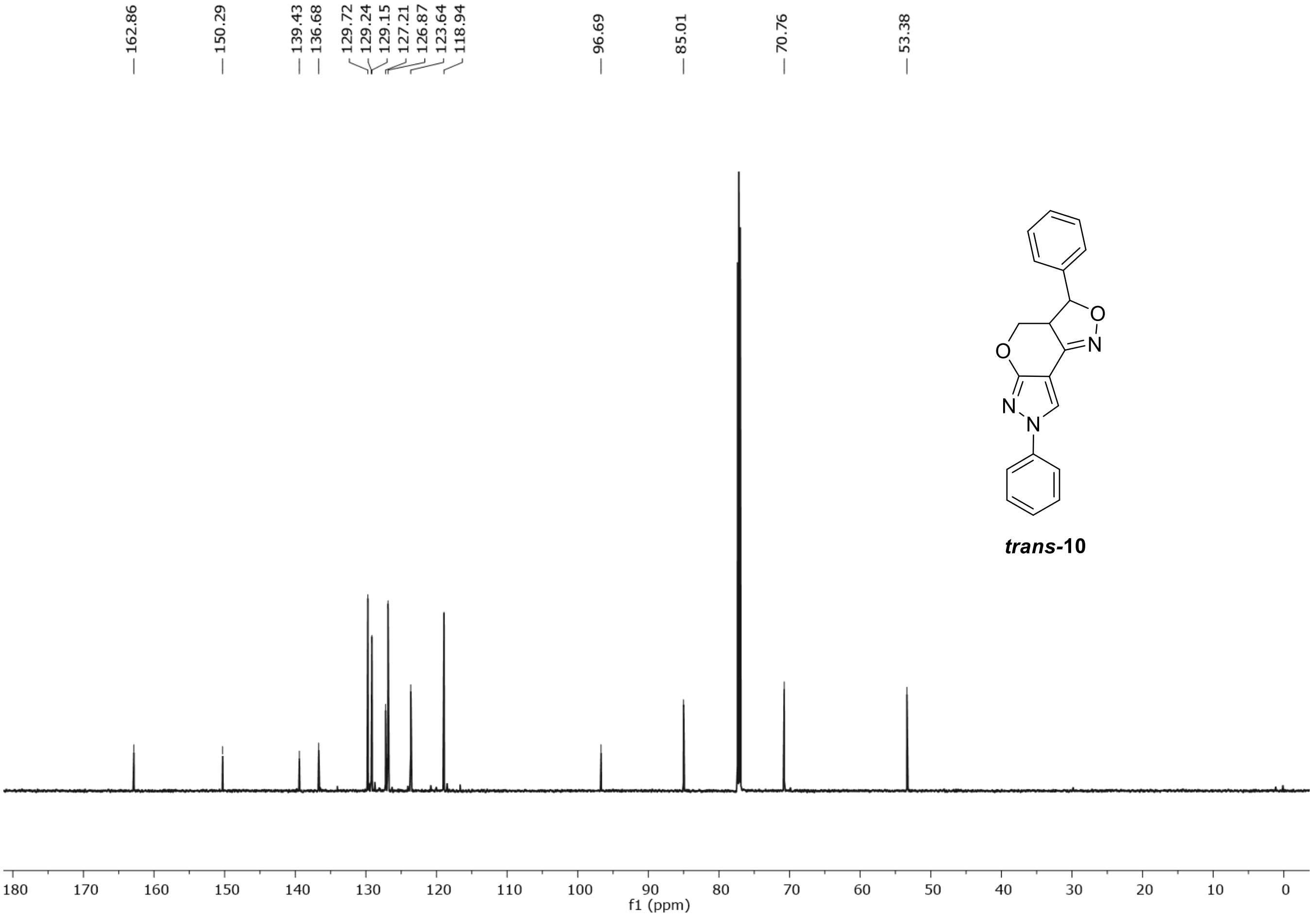

Figure S56. 3,7-Diphenyl-3a,4-dihydro-3*H*,7*H*-pyrazolo[4′,3′:5,6]pyrano[4,3-*c*][1,2]oxazole (*trans*-10).^{13}C NMR spectrum (176 MHz, CDCl$_3$).

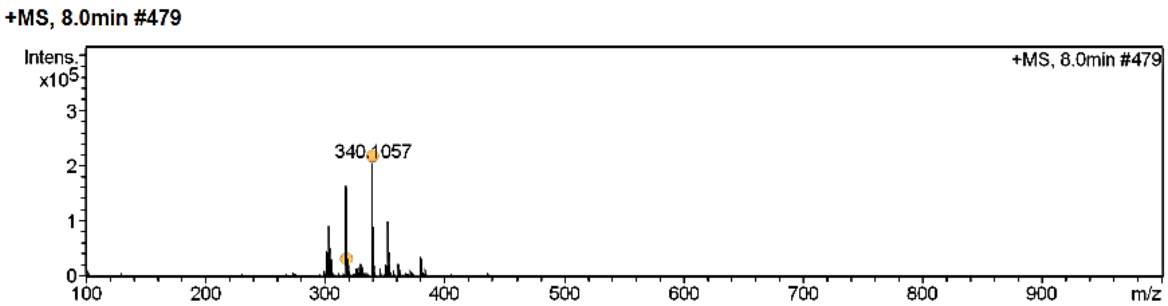

Meas. m/z	#	Ion Formula	m/z	err [ppm]	mSigma	# Sigma	Score	rdb	e¯ Conf	N-Rule
318.1232	1	C19H16N3O2	318.1237	1.6	42.6	1	100.00	13.5	even	ok
340.1057	1	C19H15N3NaO2	340.1056	-0.2	4.5	1	100.00	13.5	even	ok

Figure S57. **3,7-Diphenyl-3a,4-dihydro-3*H*,7*H*-pyrazolo[4′,3′:5,6]pyrano[4,3-*c*][1,2]oxazole (*trans*-10).·HRMS (ESI-TOF).**

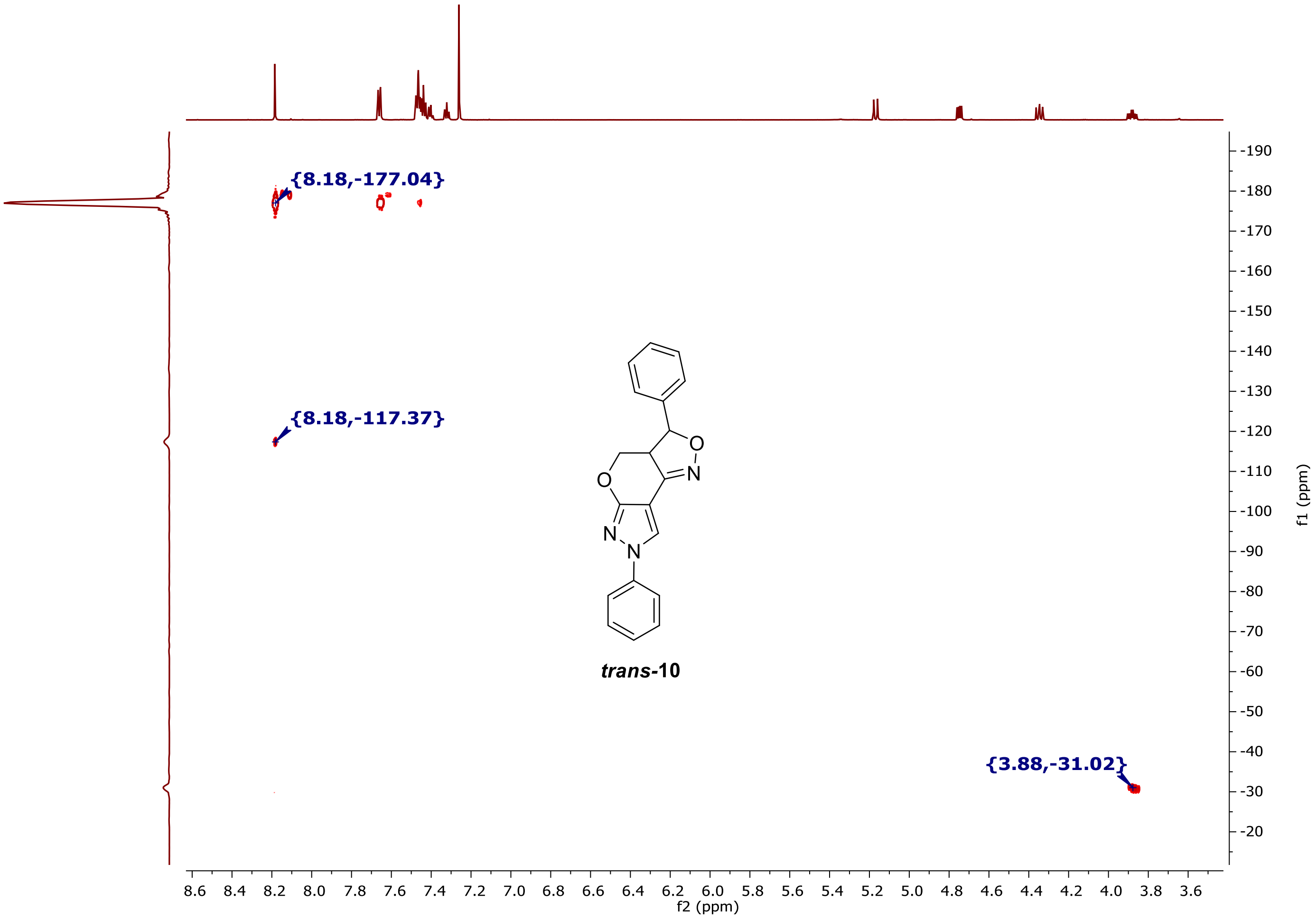

Figure S58. **3,7-Diphenyl-3a,4-dihydro-3*H*,7*H*-pyrazolo[4′,3′:5,6]pyrano[4,3-*c*][1,2]oxazole (*trans*-10).·** **^{1}H-^{15}N HMBC spectrum (700 MHz, $CDCl_3$).**

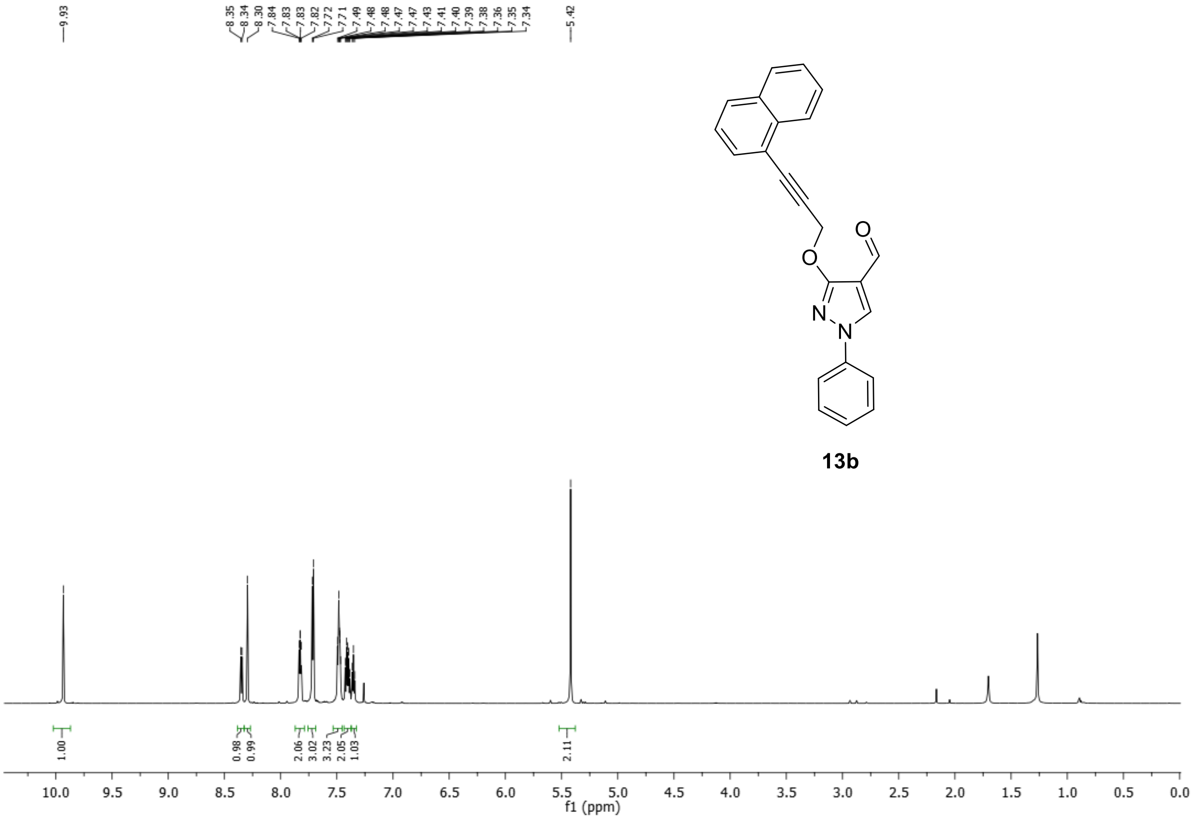

Figure S59. 3-{[3-(Naphthalen-1-yl)prop-2-yn-1-yl]oxy}-1-phenyl-1*H*-pyrazole-4-carbaldehyde (13b).^{1}**H NMR spectrum (700 MHz, CDCl$_3$).**

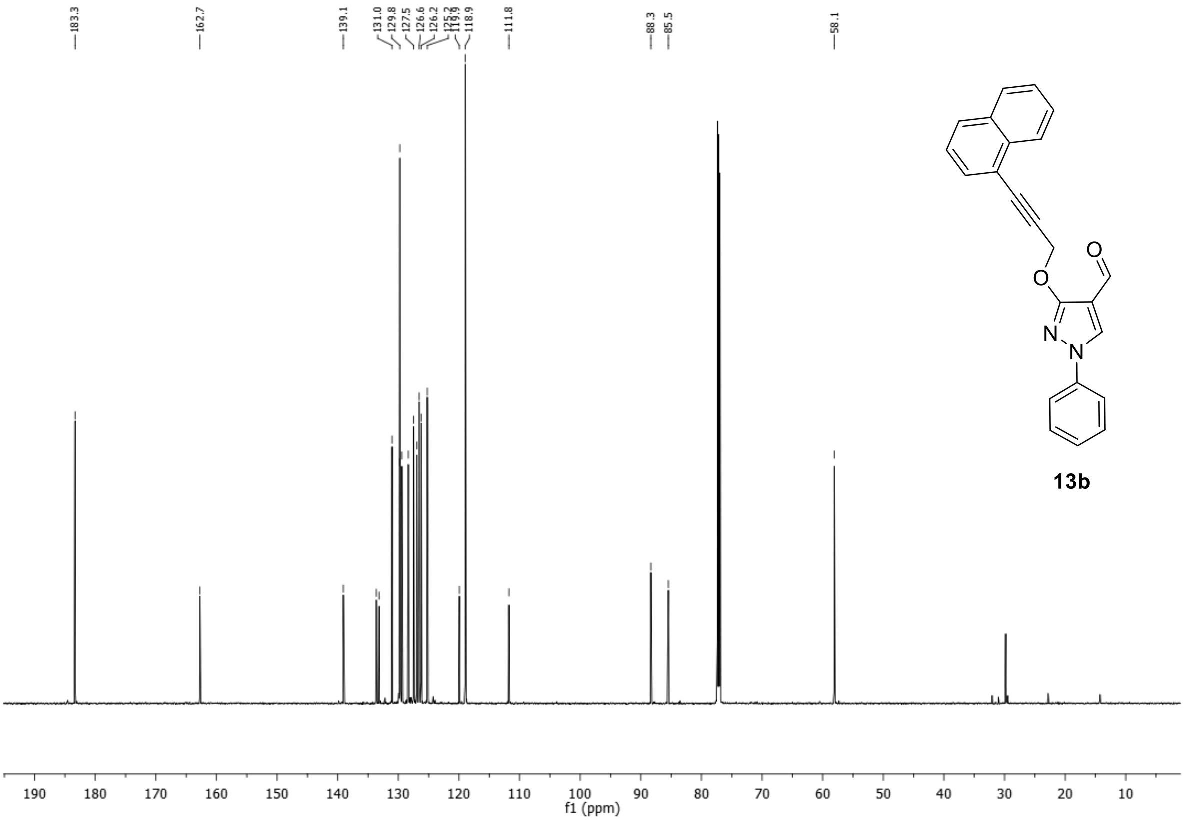

Figure S60. 3-{[3-(Naphthalen-1-yl)prop-2-yn-1-yl]oxy}-1-phenyl-1*H*-pyrazole-4-carbaldehyde (13b).^{13}**C NMR spectrum (176 MHz, CDCl$_3$).**

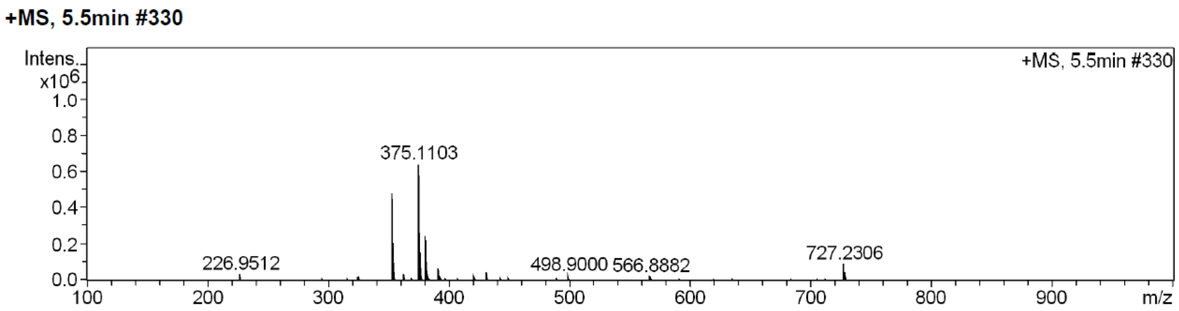

Meas. m/z	#	Ion Formula	m/z	err [ppm]	mSigma	# Sigma	Score	rdb	e⁻ Conf	N-Rule
375.1103	1	C23H16N2NaO2	375.1104	-0.4	4.4	1	100.00	16.5	even	ok

Figure S61. 3-{[3-(Naphthalen-1-yl)prop-2-yn-1-yl]oxy}-1-phenyl-1*H*-pyrazole-4-carbaldehyde (13b).·HRMS (ESI-TOF).

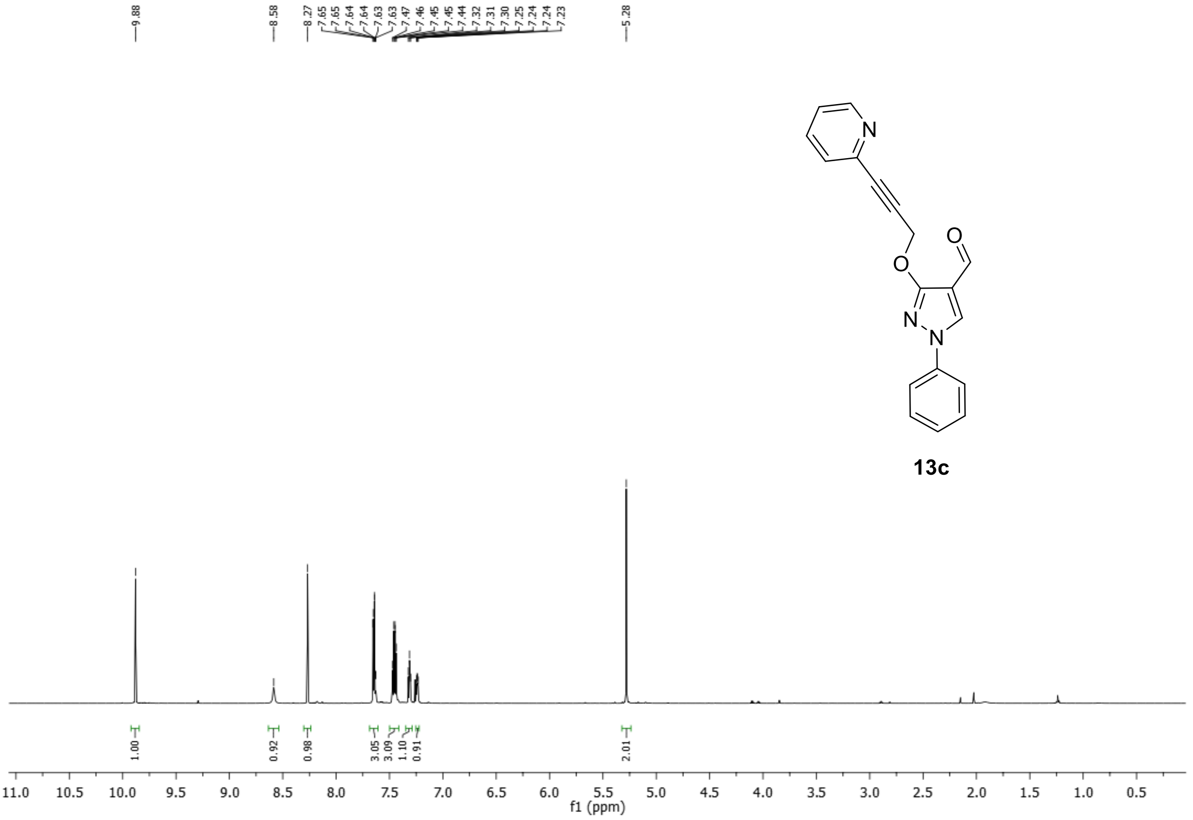

Figure S62. 1-Phenyl-3-{[3-(pyridin-2-yl)prop-2-yn-1-yl]oxy}-1*H*-pyrazole-4-carbaldehyde (13c).·^{1}H NMR spectrum (700 MHz, $CDCl_3$).

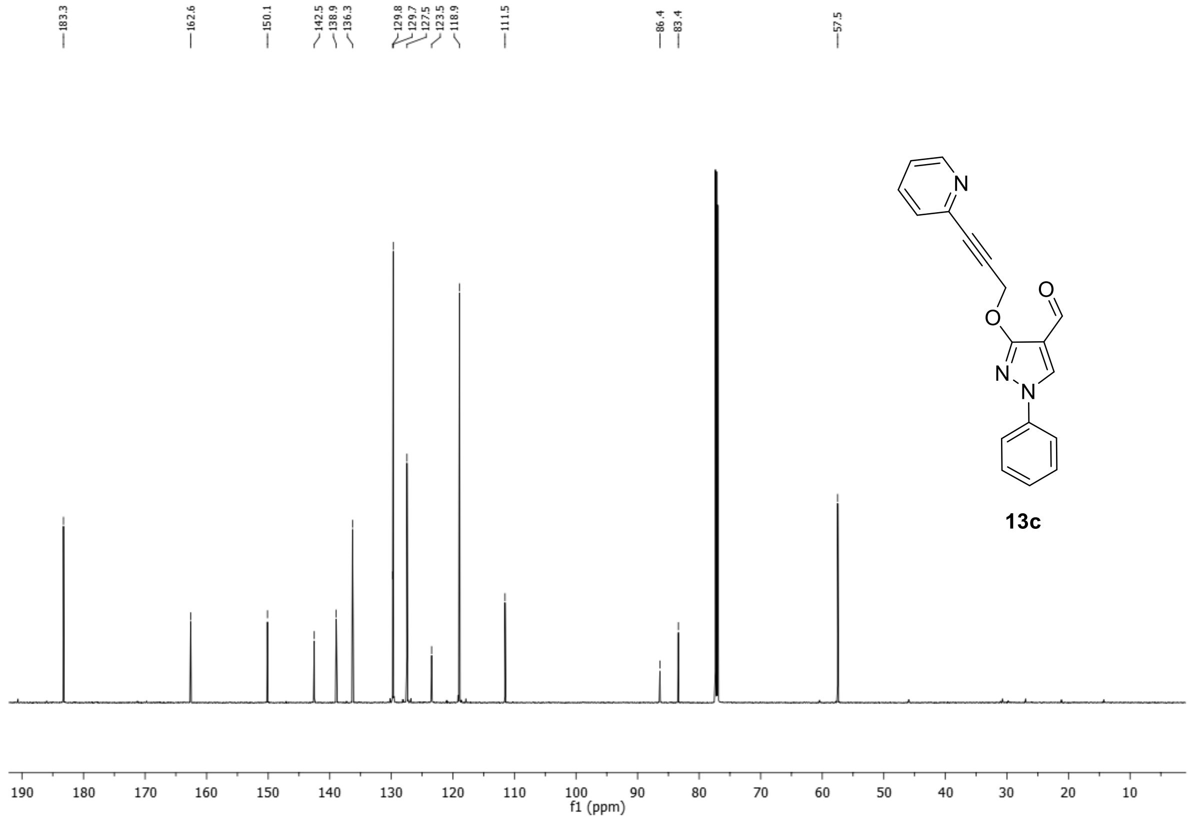

Figure S63. 1-Phenyl-3-{[3-(pyridin-2-yl)prop-2-yn-1-yl]oxy}-1*H*-pyrazole-4-carbaldehyde (13c).·^{13}C NMR spectrum (176 MHz, $CDCl_3$).

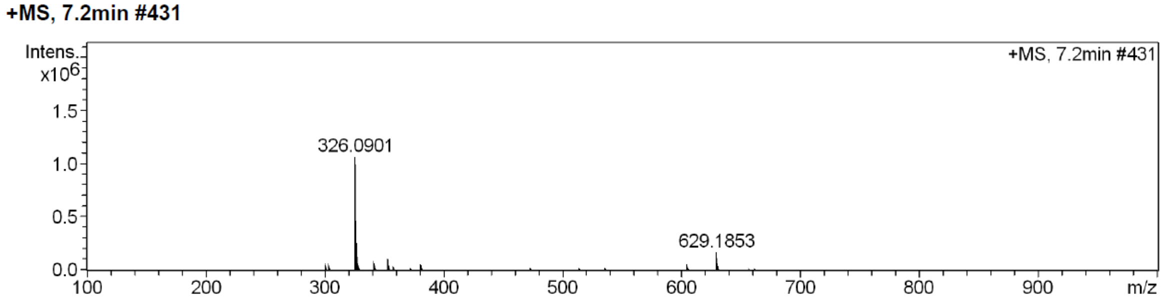

Meas. m/z	#	Ion Formula	m/z	err [ppm]	mSigma	# Sigma	Score	rdb	e⁻ Conf	N-Rule
326.0901	1	C18H13N3NaO2	326.0900	0.4	32.9	2	100.00	13.5	even	ok

Figure S64. 1-Phenyl-3-{[3-(pyridin-2-yl)prop-2-yn-1-yl]oxy}-1*H*-pyrazole-4-carbaldehyde (13c).·HRMS (ESI-TOF).

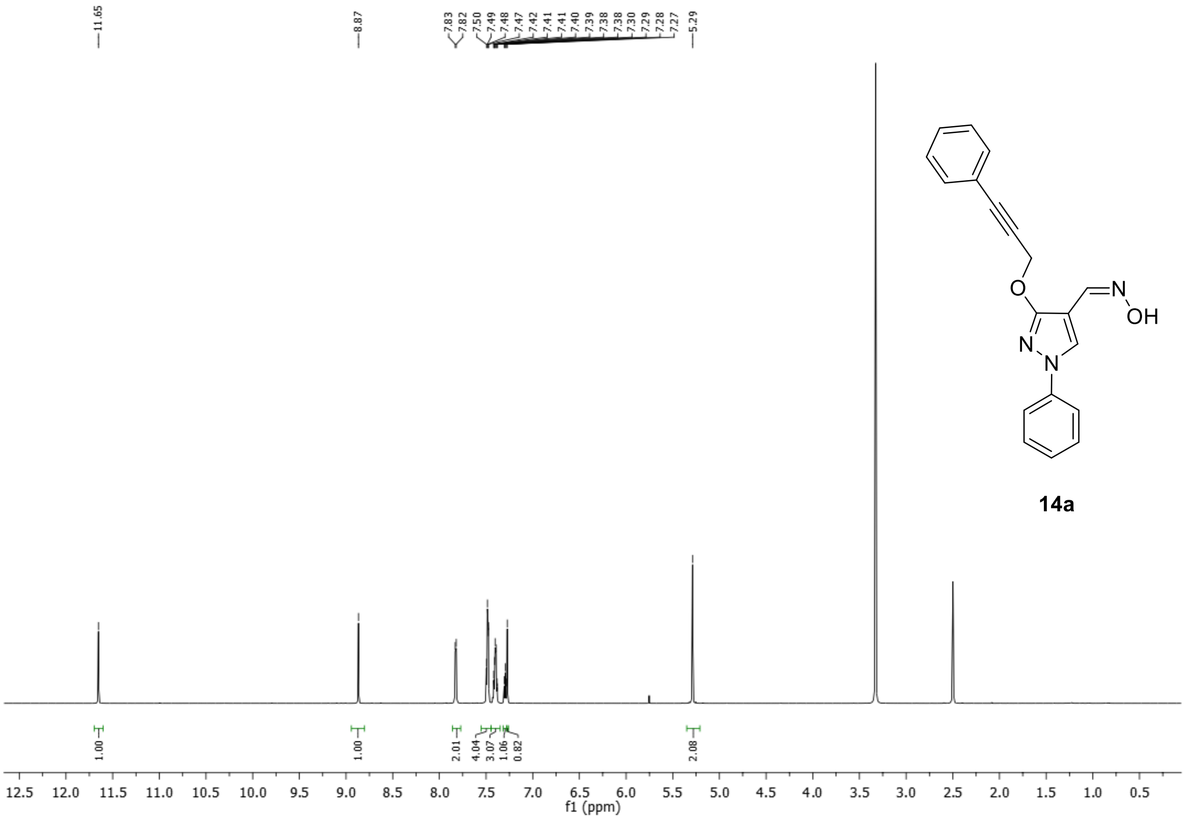

Figure S65. ***N*-[(*Z*)-{1-Phenyl-3-[(3-phenylprop-2-yn-1-yl)oxy]-1*H*-pyrazol-4-yl}methylidene]hydroxylamine (14a).^{1}H NMR spectrum (700 MHz, DMSO-d_6).**

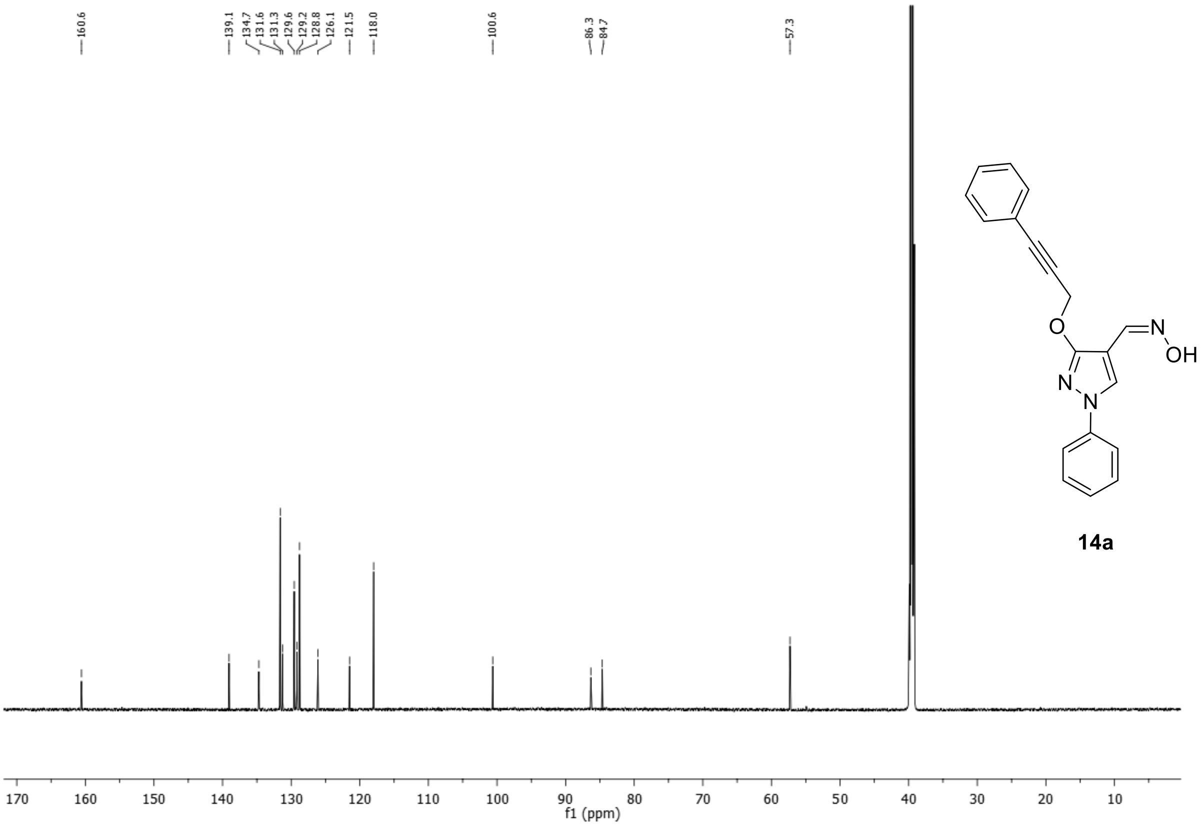

Figure S66. ***N*-[(*Z*)-{1-Phenyl-3-[(3-phenylprop-2-yn-1-yl)oxy]-1*H*-pyrazol-4-yl}methylidene]hydroxylamine (14a).^{13}C NMR spectrum (176 MHz, DMSO-d_6).**

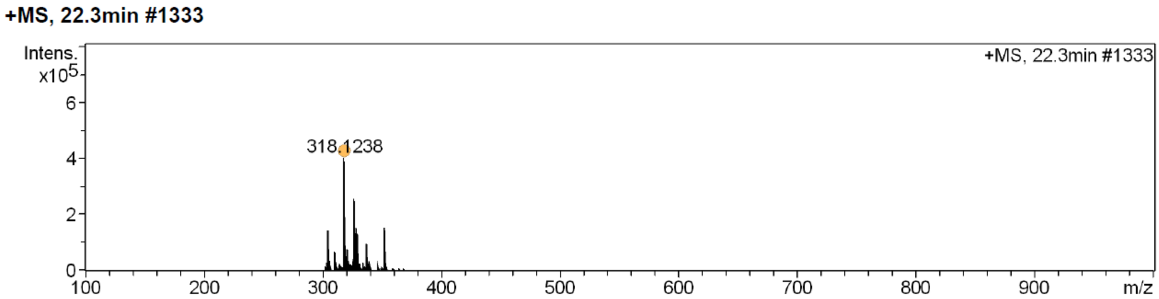

Meas. m/z	#	Ion Formula	m/z	err [ppm]	mSigma	# Sigma	Score	rdb	e⁻ Conf	N-Rule
318.1238	1	C19H16N3O2	318.1237	0.3	10.9	1	100.00	13.5	even	ok

Figure S67. ***N*-[(*Z*)-{1-Phenyl-3-[(3-phenylprop-2-yn-1-yl)oxy]-1*H*-pyrazol-4-yl}methylidene]hydroxylamine (14a).·HRMS (ESI-TOF).**

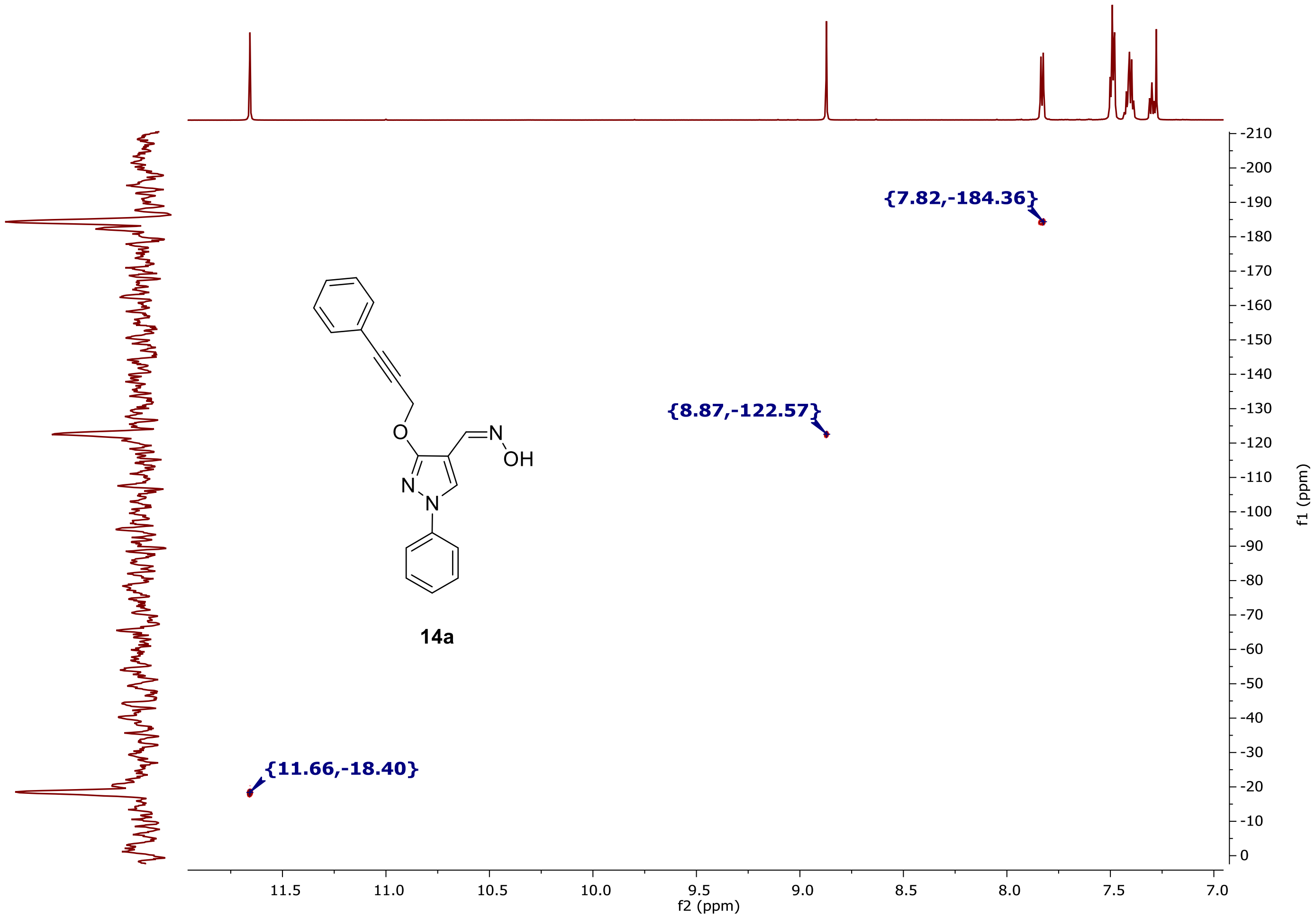

Figure S68. ***N*-[(*Z*)-{1-Phenyl-3-[(3-phenylprop-2-yn-1-yl)oxy]-1*H*-pyrazol-4-yl}methylidene] hydroxylamine (14a).· ^{1}H-^{15}N LR-HSQMBC spectrum (176 MHz, DMSO-*d*$_6$).**

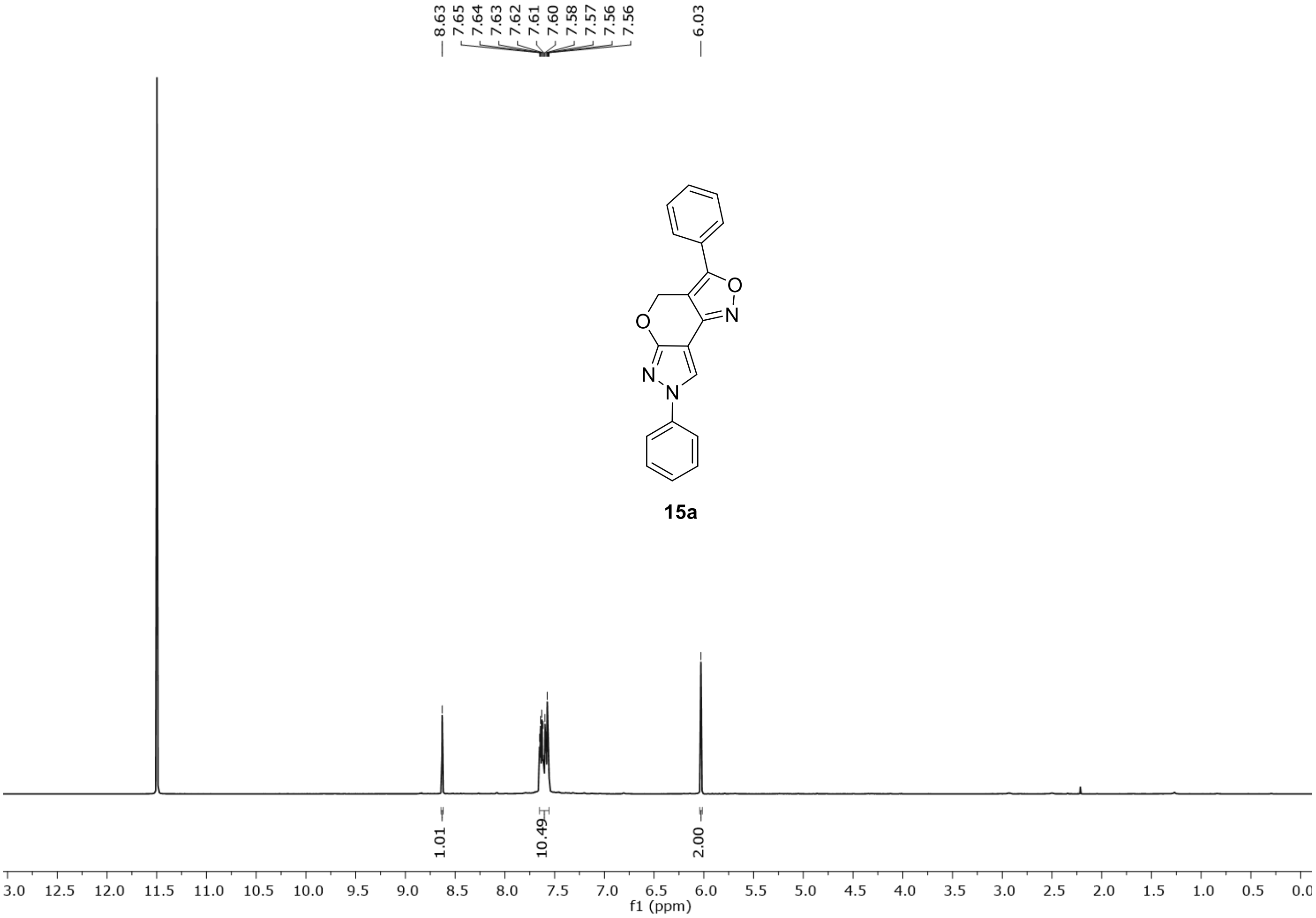

Figure S69. 3,7-Diphenyl-4*H*,7*H*-pyrazolo[4',3':5,6]pyrano[4,3-*c*]oxazole (15a).$^{.1}$**H NMR spectrum (700 MHz, TFA-*d*).**

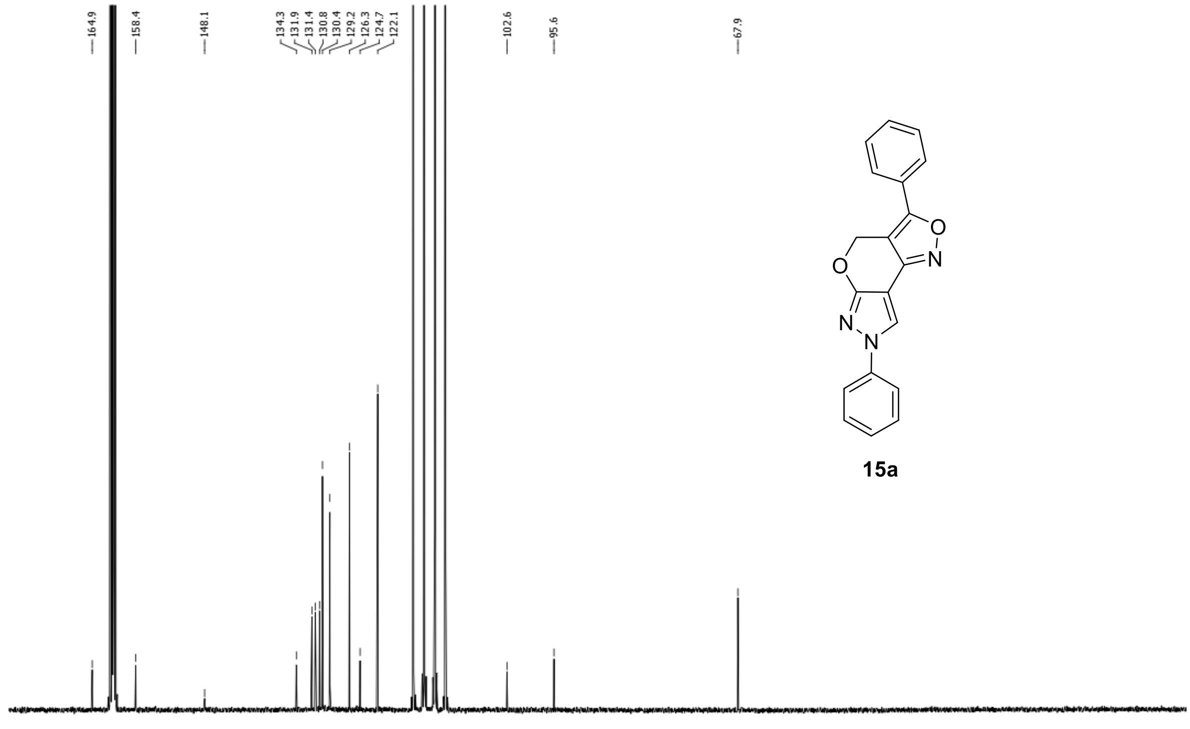

Figure S70. 3,7-Diphenyl-4*H*,7*H*-pyrazolo[4',3':5,6]pyrano[4,3-*c*]oxazole (15a).$^{.13}$**C NMR spectrum (176 MHz, TFA-*d*).**

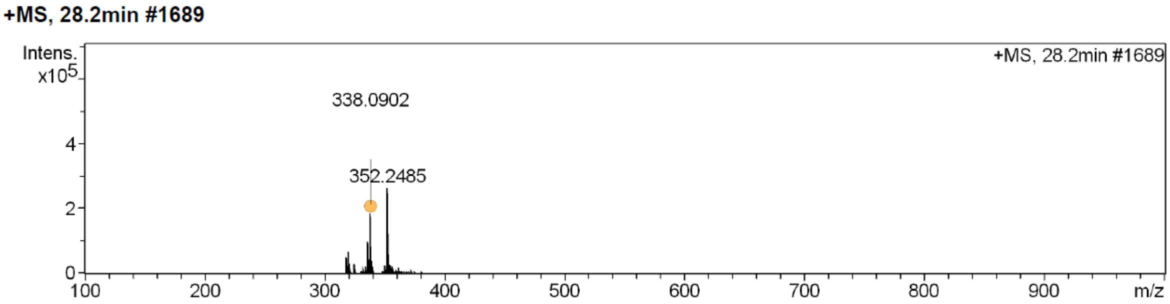

Meas. m/z	#	Ion Formula	m/z	err [ppm]	mSigma	# Sigma	Score	rdb	e⁻ Conf	N-Rule
338.0902	1	C19H13N3NaO2	338.0900	-0.7	15.8	1	100.00	14.5	even	ok

Figure S71. 3,7-Diphenyl-4*H*,7*H*-pyrazolo[4',3':5,6]pyrano[4,3-*c*]oxazole (15a).·HRMS (ESI-TOF).

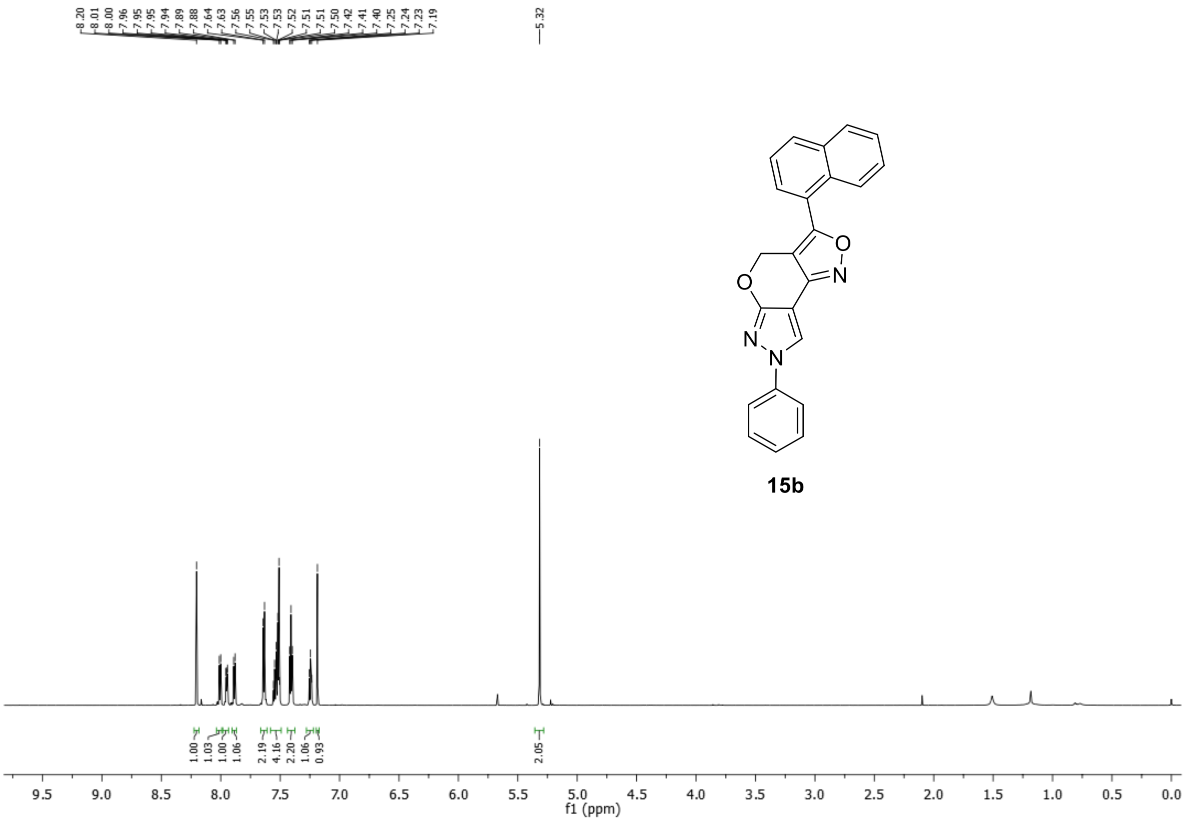

Figure S72. 3-(Naphthalen-1-yl)-7-phenyl-4,7-dihydropyrazolo[4',3':5,6]pyrano[4,3-*c*]oxazole (15b).·^{1}H NMR spectrum (700 MHz,).

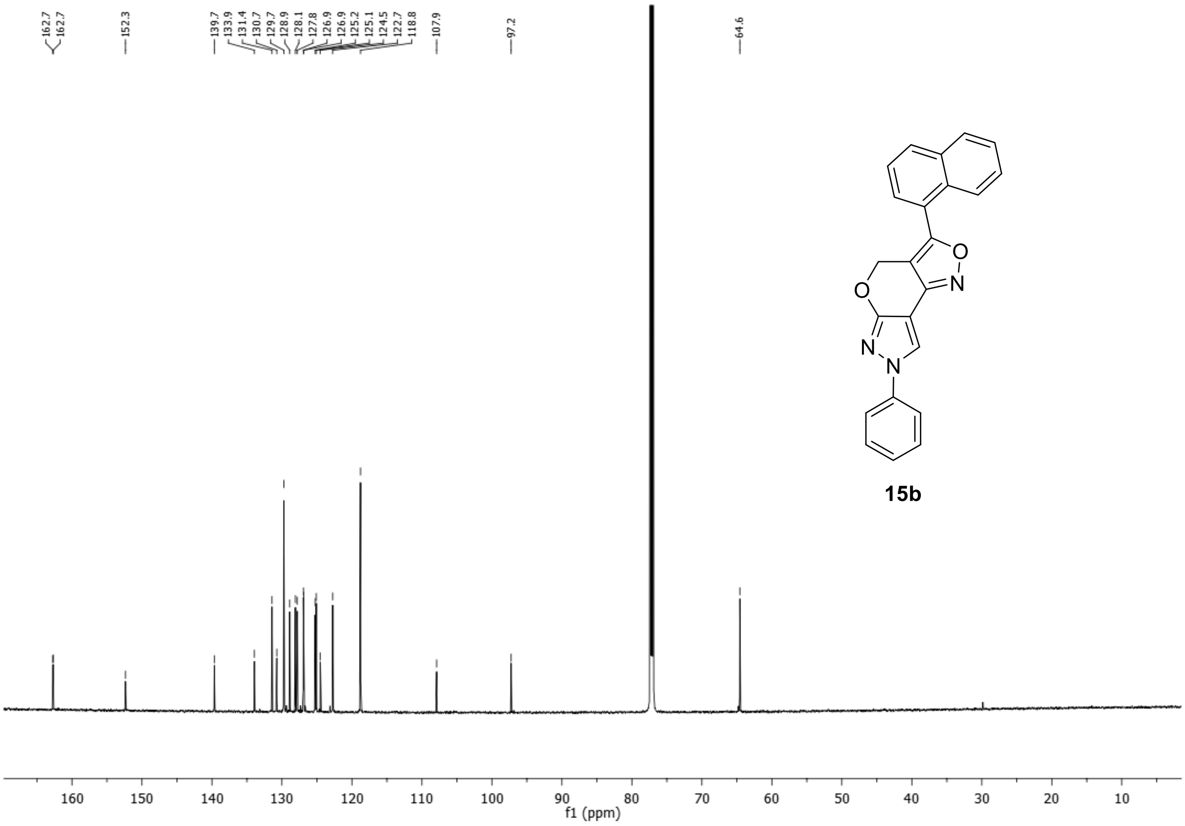

Figure S73. 3-(Naphthalen-1-yl)-7-phenyl-4,7-dihydropyrazolo[4',3':5,6]pyrano[4,3-*c*]oxazole (15b).^{13}C NMR spectrum (176 MHz,).

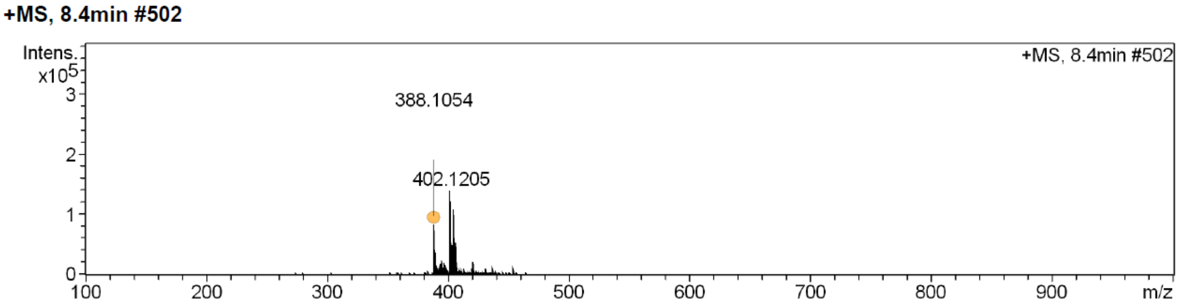

Meas. m/z	#	Ion Formula	m/z	err [ppm]	mSigma	# Sigma	Score	rdb	e⁻ Conf	N-Rule
388.1054	1	C23H15N3NaO2	388.1056	-0.7	137.0	1	100.00	17.5	even	ok

Figure S74. 3-(Naphthalen-1-yl)-7-phenyl-4,7-dihydropyrazolo[4',3':5,6]pyrano[4,3-*c*]oxazole (15b). HRMS (ESI-TOF).

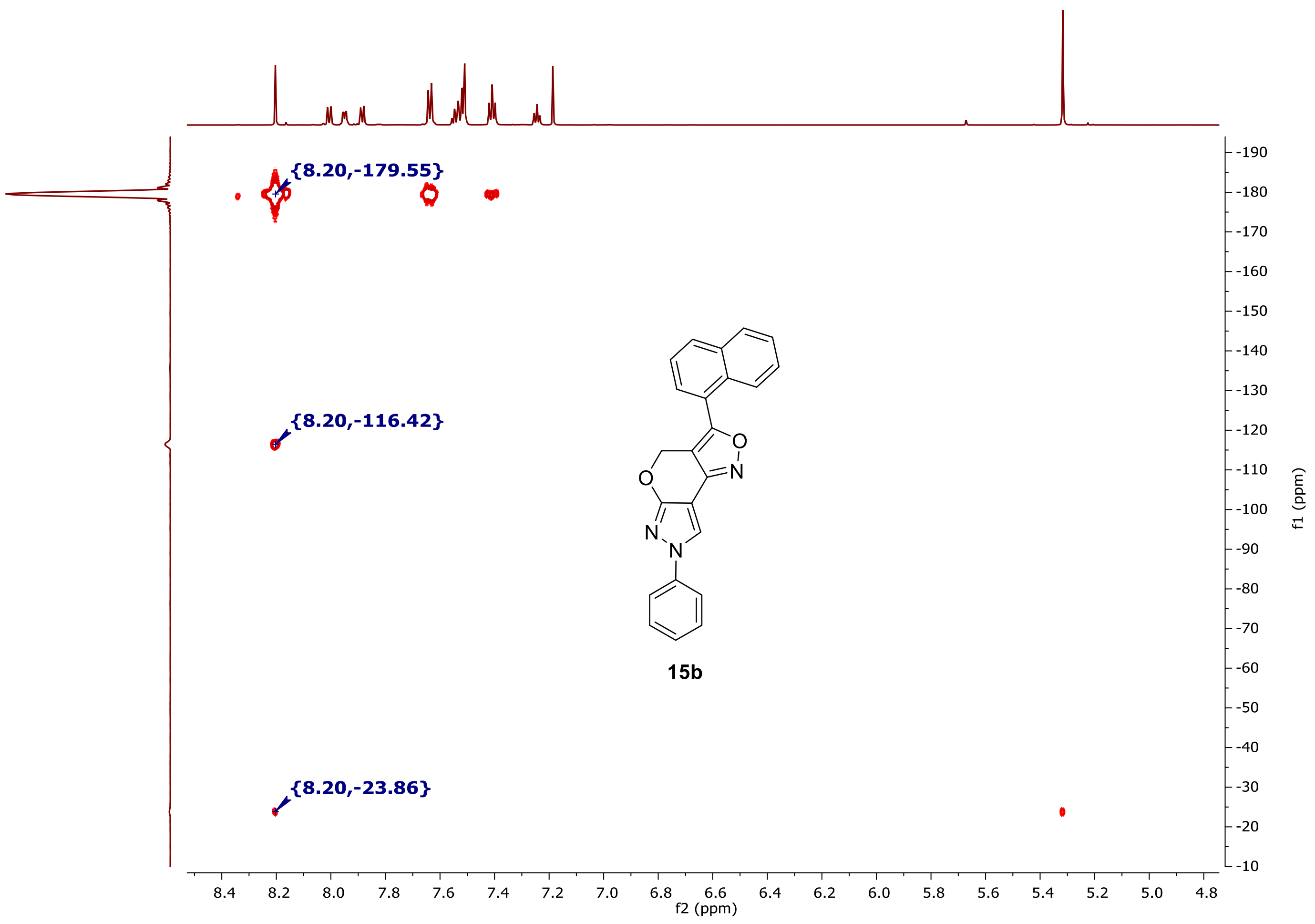

Figure S75. 3-(Naphthalen-1-yl)-7-phenyl-4,7-dihydropyrazolo[4',3':5,6]pyrano[4,3-*c*]oxazole (15b). ^{1}H-^{15}N HMBC spectrum (700 MHz, CDCl$_3$).

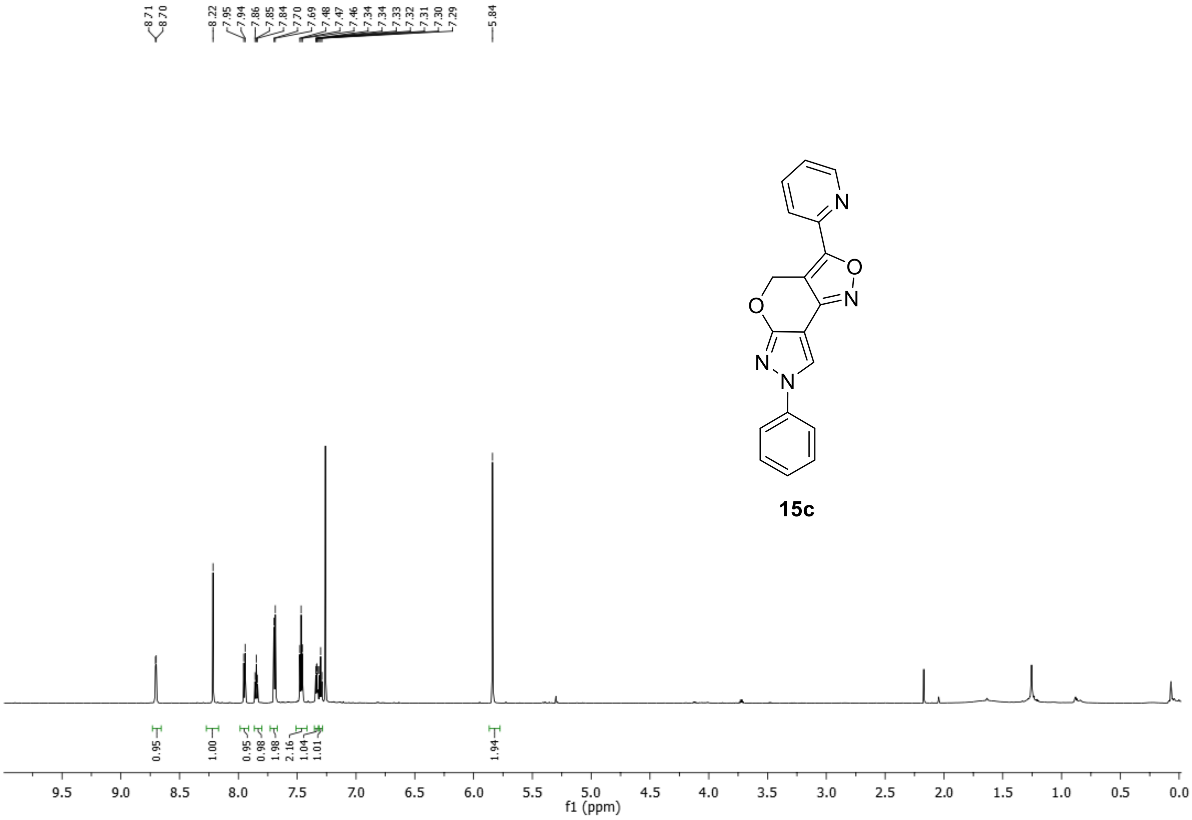

Figure S76. 7-Phenyl-3-(pyridin-2-yl)-4,7-dihydropyrazolo[4',3':5,6]pyrano[4,3-*c*]oxazole (15c).$^{\cdot 1}$**H NMR spectrum (700 MHz, $CDCl_3$).**

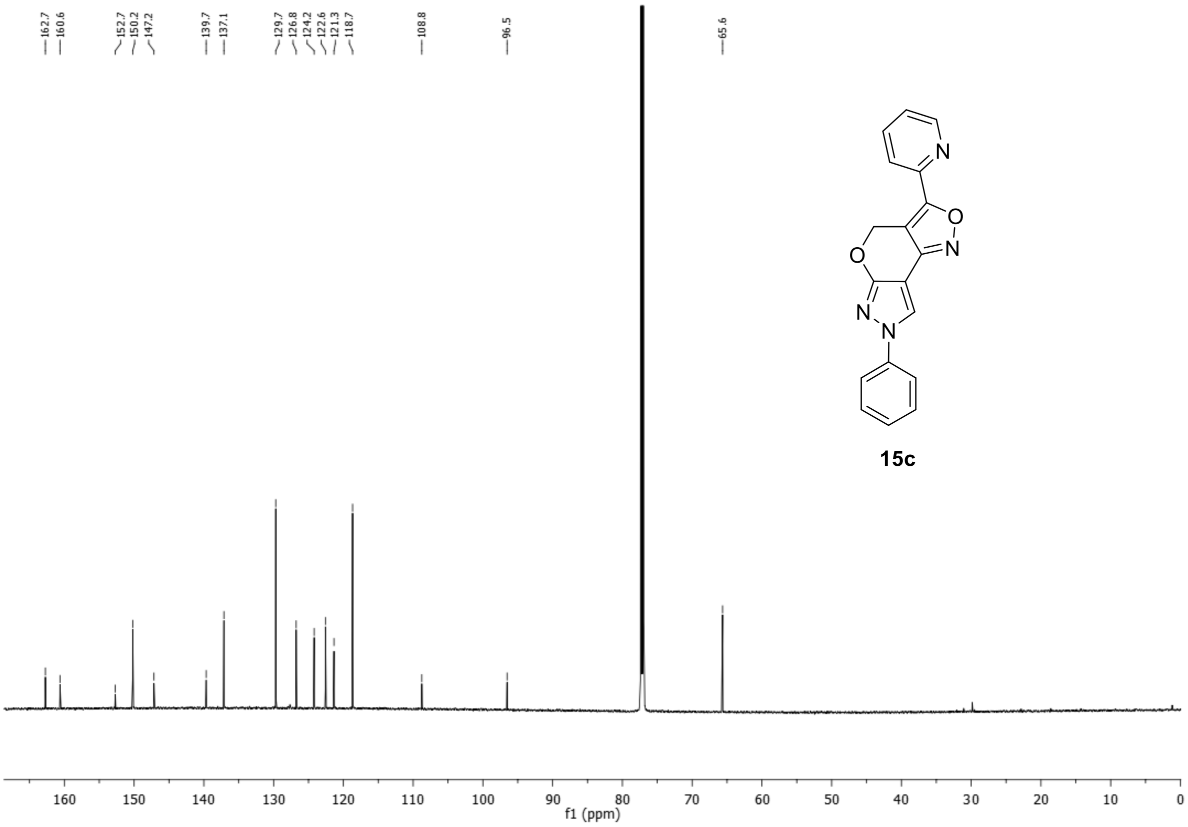

Figure S77. 7-Phenyl-3-(pyridin-2-yl)-4,7-dihydropyrazolo[4',3':5,6]pyrano[4,3-*c*]oxazole (15c).$^{\cdot 13}$**C NMR spectrum (176 MHz, $CDCl_3$).**

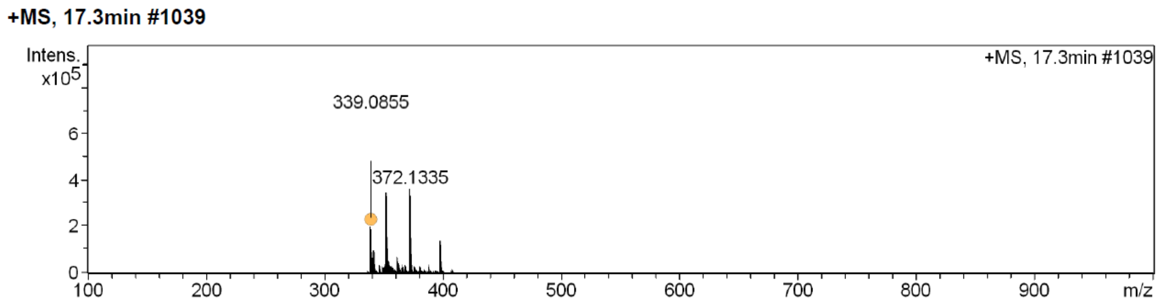

Meas. m/z	#	Ion Formula	m/z	err [ppm]	mSigma	# Sigma	Score	rdb	e⁻ Conf	N-Rule
339.0855	1	C18H12N4NaO2	339.0852	-0.7	163.5	2	100.00	14.5	even	ok

Figure S78. 7-Phenyl-3-(pyridin-2-yl)-4,7-dihydropyrazolo[4',3':5,6]pyrano[4,3-*c*]oxazole (15c). HRMS (ESI-TOF).

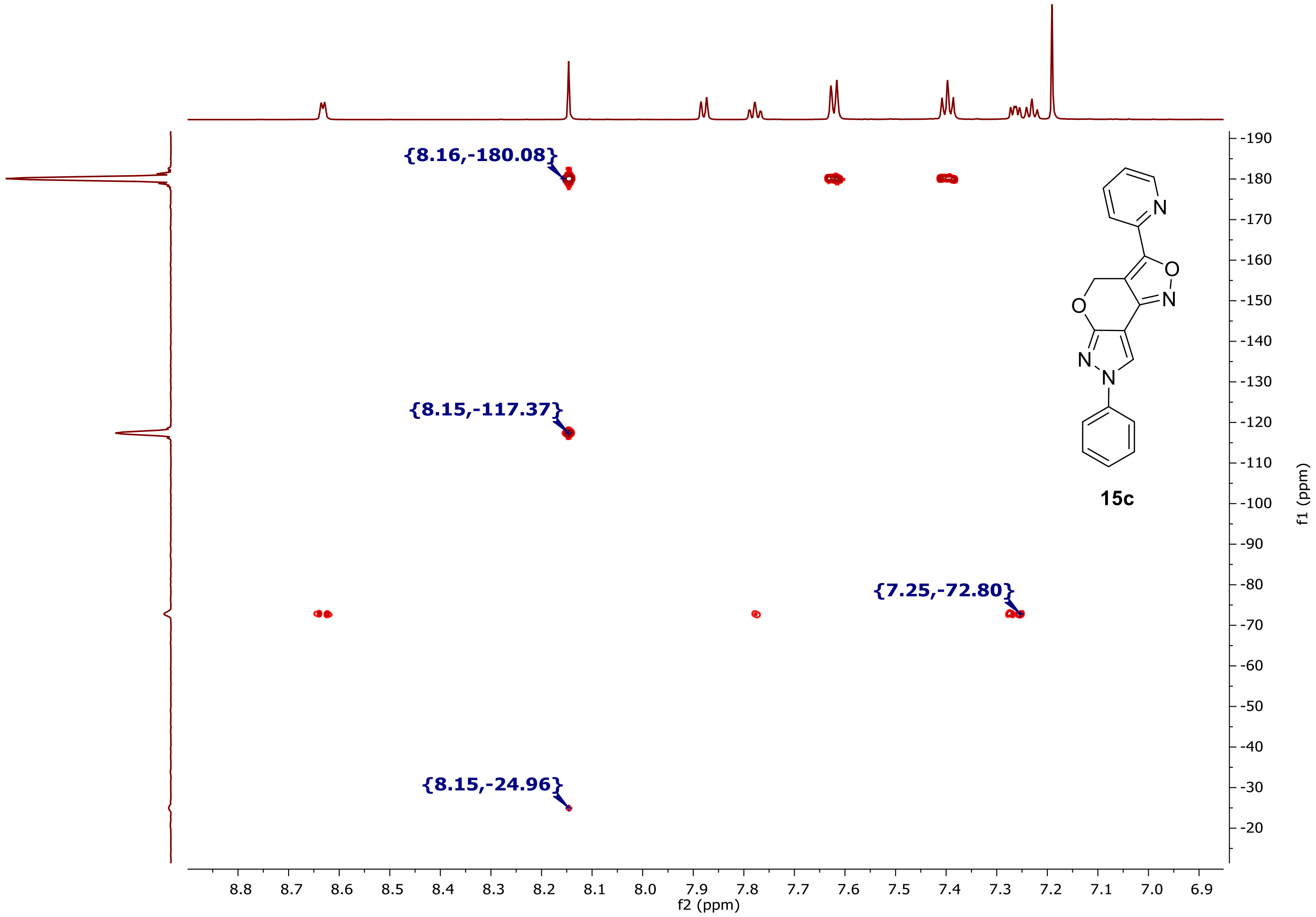

Figure S79. 7-Phenyl-3-(pyridin-2-yl)-4,7-dihydropyrazolo[4',3':5,6]pyrano[4,3-*c*]oxazole (15c). ^{1}**H-**^{15}**N HMBC spectrum (700 MHz, CDCl**$_3$**).**

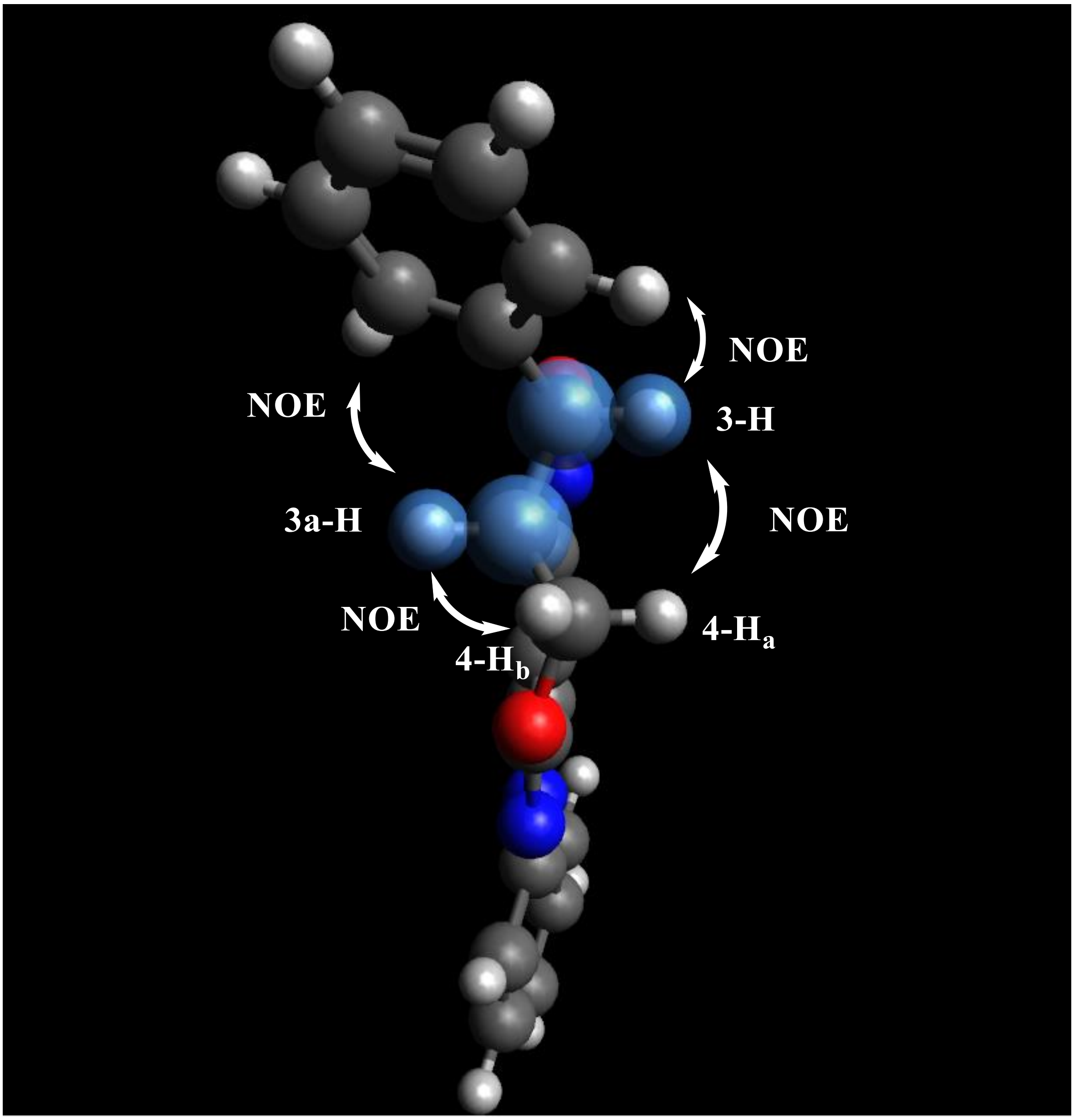

Figure S80. 3,7-Diphenyl-3a,4-dihydro-3*H*,7*H*-pyrazolo[4′,3′:5,6]pyrano[4,3-*c*][1,2]oxazole (*trans*-10). B3LYP/def2-TZVP optimized structure of *trans*-10.

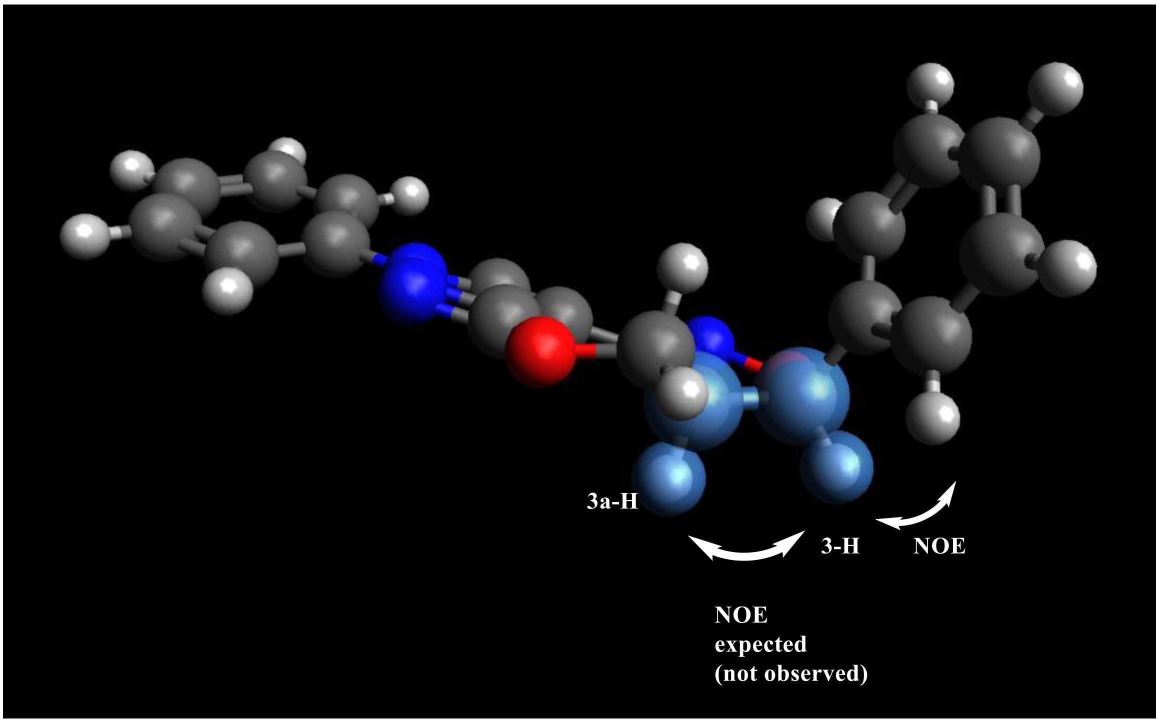

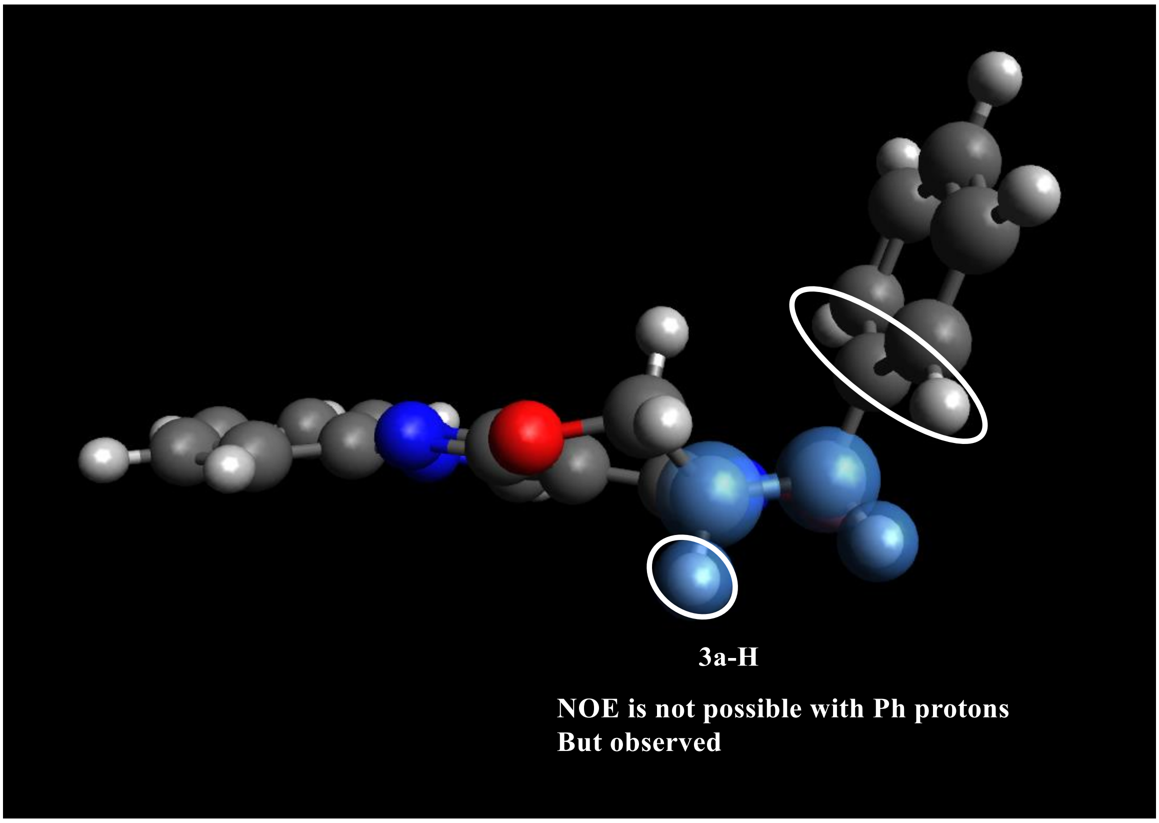

Figure S81. **3,7-Diphenyl-3a,4-dihydro-3*H*,7*H*-pyrazolo[4′,3′:5,6]pyrano[4,3-*c*][1,2]oxazole (10). B3LYP/def2-TZVP optimized structure of *cis*-10.**
